# CDCA8/SNAI2 Complex Activates CD44 to Promote Proliferation and Invasion of Pancreatic Ductal Adenocarcinoma

**DOI:** 10.3390/cancers14215434

**Published:** 2022-11-04

**Authors:** Jichun Gu, Yujie Guo, Jiali Du, Lei Kong, Junyuan Deng, Baian Tao, Hengchao Li, Chen Jin, Deliang Fu, Ji Li

**Affiliations:** 1 Department of Pancreatic Surgery, Huashan Hospital, Fudan University, Shanghai 200040, China; 2 Pancreatic Disease Institute, Fudan University, Shanghai 200040, China

**Keywords:** CDCA8, CD44, SNAI2, PDAC, proliferation, invasion

## Abstract

**Simple Summary:**

There is an urgent need to find an effective therapeutic target for pancreatic cancer owing to late diagnosis, tumor metastasis, and current ineffective targeted drugs. We aimed to identified potential targets for the treatment of pancreatic cancer. In this study, the specific mechanism by which the CDCA8 contributes to pancreatic cancer progression via the activation of CD44 was clarified, and CDCA8 knockdown inhibited the proliferation and metastasis of pancreatic cancer. This finding may provide a promising target for future targeted therapies of pancreatic cancer.

**Abstract:**

(1) Background: Recently, cell division cycle associated 8 (CDCA8) was found to be overexpressed in pancreatic ductal adenocarcinoma (PDAC). Here, we aimed to explore the specific mechanism of action of CDCA8 in PDAC progression. (2) Methods: All human PDAC samples and clinical data were collected from Huashan Hospital, Fudan University. All experimental studies were carried out using many in vitro and in vivo assays, including lentiviral transfection, real-time quantitative polymerase chain reaction (qPCR), western blotting, co-immunoprecipitation (Co-IP), chromatin IP (ChIP)-qPCR, dual-luciferase reporter, and in vivo imaging assays. (3) Results: Clinical data analysis of human PDAC samples revealed that CDCA8 overexpression were positively and negatively associated with tumor grade (*p* = 0.007) and overall survival (*p* = 0.045), respectively. CDCA8 knockdown inhibited PDAC proliferation and invasion in in vitro and in vivo assays. CD44 was also up-regulated by CDCA8 during PDAC progression. CDCA8 could be combined with SNAI2 to form a CDCA8/SNAI2 complex to integrate with the CD44 promoter as indicated through ChIP-qPCR and dual-luciferase reporter assays. (4) Conclusion: We showed that CDCA8-CD44 axis plays a key role in the proliferation and invasion of PDAC, which provides a potential target for treatment.

## 1. Introduction

Pancreatic ductal adenocarcinoma (PDAC) is one of the deadliest malignant solid tumors worldwide. The estimated mortality of pancreatic cancer ranks fourth among all tumor types in China and is expected to increase in the future [1]. Although a number of studies have been dedicated to PDAC during the past decades, the overall 5-year survival rate among patients with PDAC is still <8% [2]. Surgical resection, chemotherapy, and radiotherapy are the most common treatments for PDAC. However, these treatments have limited effects in prolonging the survival of patients because of the high recurrence rate [3,4]. Recently, targeted therapy has become a promising treatment for PDAC. There are some common therapeutic targets, such as epidermal growth factor receptor (EGFR), vascular endothelial growth factor (VEGF), and mammalian target of rapamycin (mTOR) [5]. These targeted drugs have limited efficacy [6,7]. Thus, there is an urgent need for new therapeutic targets for patients with PDAC.

Cell division cycle associated 8 (CDCA8), also known as borelin, is a member of the chromosomal passenger complex (CPC), which is a group of highly conserved complexes that regulate cell division [8,9]. CDCA8 mainly exists in human embryonic stem cells (hESCs) and tumor cells and is rarely expressed in normal tissues [10]. Recent studies have shown that CDCA8 is overexpressed in various cancer cells, such as bladder cancer, breast cancer, and cutaneous melanoma. Its activation is essential for the proliferation and invasion of tumor cells [11,12].

CD44 is a non-kinase transmembrane receptor that regulates cell growth and differentiation and is divided into standard (CD44s) and variant (CD44v) isoforms [13]. CD44 plays an important role in regulating hyaluronic acid metabolism, releasing cytokines, and activating lymphocytes in normal tissues [14]. Aberrant levels of CD44 have been observed in colorectal and prostate cancers [15,16]. Even in PDAC, it has been found that the overexpression of CD44 could promote metastasis of tumor cells and reduce response to chemotherapy [17]. 

In the present study, we explored the vital role of CDCA8 in promoting the proliferation and progression of PDAC and further described the specific signalling pathway of the CDCA8-CD44 axis to enhance the proliferation and invasion of PDAC.

## 2. Materials and Methods

### 2.1. Cell Culture and Transfection

Human PDAC cell lines PANC-1, SW-1990, AsPC-1, and BxPC-3, and human pancreatic ductal epithelial cell line HPDE6-C7 were obtained from BioSCI Res Co., Ltd. (Shanghai, China) and cultured in 6-well plates in Dulbecco’s modified Eagle medium (Corning Inc., One Riverfront Plaza Corning, New York, NY, USA) containing 10% Ausbian fetal bovine serum (FBS) (Beijing Diyi Biology Science and Technology Co., Ltd., Beijing, China). All the cell lines were incubated at 37 °C in a 5% CO_2_ incubator.

To generate CD44 knockdown, CDCA8 knockdown stable cell lines, PANC-1 and SW-1990 cells, were transfected with lentivirus (LV)-shCD44, LV-shCDCA8, or viral particles (BioSCI Res Co., Ltd., Shanghai, China), all of which were marked with green fluorescent protein (GFP) and selected via fluorescence (fluorescence efficiency > 80%).

To generate stable CDCA8 overexpression and CD44 knockdown + CDCA8 overexpressed cell lines, PANC-1 and SW-1990 cells were transfected with LV-CDCA8, LV-shCD44+LV-CDCA8, or viral particles (BioSCI Res Co., Ltd., Shanghai, China) and selected via fluorescence (fluorescence efficiency > 80%). 

To avoid off-target effect, we performed in vitro assays using two different RNAi sequences of CDCA8 (RNAi-10540 [shCDCA8-2], RNAi-10541 [shCDCA8-3], Figures S4 and S5)

### 2.2. Human PDAC Tissues

All human PDAC samples and clinical data in this study were collected from Huashan Hospital, Fudan University. Immunohistochemical (IHC) assays were conducted on the tumor and adjacent tissues. CDCA8 is located in both the nucleus and cytoplasm of PDAC tumor tissues. The expression level of CDCA8 was classified into low and high groups according to the staining index (scores, 0–12; staining index = staining intensity × percentage of positive cells). According to staining shades of cytoplasm, membrane, or nucleus, the stain intensity was divided into four grades: 0, negative; 1, low; 2, medium; 3, high. Based on the proportion of stained cells, the percentage of positive cells was also divided into five grades: 0, 0% stained cells; 1, 0% < stained cells < 25%; 2, 25% ≤ stained cells < 50%; 3, 50% ≤ stained cells < 75%; 4, 75% ≤ stained cells. IHC data for each sample were acquired by an experienced pathologist within four visual fields.

### 2.3. Immunohistochemistry (IHC) Assay

All paraffin sections were dewaxed and rehydrated using dimethylbenzene and 100% alcohol (Sinopharm Co., Ltd., Beijing, China). Antigen retrieval was performed using citrate buffer (Maixin Biotechnology Development Co., Ltd., Fuzhou, China) and 3% hydrogen peroxide solution (Hengyuan Biotechnology Co., Ltd., Shanghai, China). Subsequently, sections were blocked with 5% goat serum for 30 min. All sections were incubated with primary antibodies at 37 °C for 1 h, washed, incubated with peroxidase-conjugated secondary antibodies at 37 °C for 1 h, and then washed. Diaminobenzidine (DAB) chromogenic solution was used to stain the sections, and haematoxylin (BaSO Co., Ltd., Zhuhai, China) was used for counterstaining. All the sections were dehydrated and sealed with dimethylbenzene and gradient alcohol (Sinopharm Co., Ltd., Beijing, China).

### 2.4. Real-Time Quantitative Polymerase Chain Reaction Assay (qPCR)

Total RNA was extracted from PANC-1, SW-1990, AsPC-1, BxPC-3, and HPDE6-C7 cells using TRIzol reagent (Sigma-Aldrich, Merck KGaA, Darmstadt, Germany) according to the manufacturer’s instructions. Complementary DNA was synthesized using Hiscript QRT Supermix for qPCR (+gDNA WIPER) (Vazyme Biotech Co., Ltd., Nanjing, China). Transcription was quantified using AceQ qPCR SYBR Green Master Mix (Vazyme Biotech Co., Ltd., Nanjing, China) on a VII7 machine (Applied Biosystems CN, Thermo Fisher Scientific, Shanghai, China). All data (relative mRNA levels of CDCA8 and CD44) were normalized to the reference gene of glyceraldehyde-3-phosphate dehydrogenase (GAPDH). The primer sequences are listed in Table S2.

### 2.5. Western Blotting Assay

Cell lysates from PANC-1 and SW-1990 cells were quantified using the BCA Protein Assay Kit (HyClone Co., Logan, Utah, USA) and then separated by 12% sodium dodecyl sulfate-polyacrylamide gel electrophoresis (SDS-PAGE) (BioSCI Res Co., Ltd., Shanghai, China). Subsequently, all the proteins were transferred onto polyvinylidene difluoride (PVDF) membranes (BioSCI Res Co., Ltd., Shanghai, China). Tris-HCl Tween (TBWT) buffer containing 5% skimmed milk (BioSCI Res Co., Ltd., Shanghai, China) was utilized to block the membranes at 4 °C for 1 h. The membranes were then incubated with CDCA8 and CD44 primary antibodies (Abcam plc., Cambridge, UK and Cell Signaling Technology Inc., Danvers, MA, USA) at a 1:1000 dilution (4 °C, overnight). Finally, the membranes were incubated with peroxidase-conjugated secondary antibodies at a 1:1000 dilution (4 °C, 1 h), and chemiluminescence was developed using Immobilon Western Chemiluminescent HRP Substrote (Millipore, Merck KGaA, Darmstadt, Germany). Relative protein level was normalized to that of GAPDH. The original Western Blotting can be found in the supplementary Figure S6.

### 2.6. MTT Assay

PANC-1 and SW-1990 cells were plated in 96-well plates at a density of 2 × 10^3^ cells/well and cultured. Cells in each well were treated with 3-(4,5-Dimethyl-2-Thiazolyl)-2,5-diphenyl tetrazolium bromide (MTT) (Gen-view Scientific Inc., El Monte, CA, USA) for 4 h and washed. Then, dimethyl sulfoxide (DMSO) (Shanghai Shiyi Chemical Reagent Co., Ltd., Shanghai, China) was added to each well, and the cells were extracted. The absorbance at 490 nm was measured using a Techan Infinite M2009PR enzyme-labelled instrument (Tecan Trading AG, Männedorf, Switzerland).

### 2.7. Cell Counting Kit-8 (CCK-8) Assay

PANC-1 and SW-1990 cells were plated in 96-well plates at a density of 2 × 10^3^ cells/well and cultured. Cells in each well were treated with CCK-8 (Gen-view Scientific Inc., El Monte, CA, USA) for 4 h and the absorbance at 450 nm was measured using a Techan Infinite M2009PR enzyme-labelled instrument (Tecan Trading AG, Männedorf, Switzerland).

### 2.8. Cell Cycle and Apoptosis Assay

PANC-1 and SW-1990 cells were fixed with 70% ethanol at 4 °C overnight and suspended in phosphate-buffered saline (PBS) (BioSCI Res Co., Ltd., Shanghai, China). All cells were washed with propidium iodide (PI, 2 mg/mL) (Sigma-Aldrich, Merck KGaA, Darmstadt, Germany), RNase A (10 mg/mL) (Takara Bio Inc., Kusatsu, Shiga, Japan), and PBS. All cells were analyzed with a flow cytometer (Guava easyCyte HT, Millipore, Merck KGaA, Darmstadt, Germany), and the percentage of cells in the G1, S, and G2 phases was calculated. Apoptosis was detected using an Annexin V Apoptosis Detection Kit the manufacturer indicated (eBioscience, Thermo Fisher Scientific, Shanghai, China).

### 2.9. Wound-Healing and Transwell Assay

For the wound healing assay, PANC-1 and SW-1990 cells were plated in 96-well plates, and scratches were made using a 96-wounding replicator (VP scientific, San Diego, CA, USA). All wells were gently washed with serum-free medium. DMEM (Corning Inc., One Riverfront Plaza Corning, New York, NY, USA) containing 0.5% Ausbian FBS (Beijing Diyi Biology Science and Technology Co., Ltd., Beijing, China) was added to the wells, and images were taken using a fluorescence microscope (Olympus Co., Tokyo, Japan) at different time points. The migration rate of tumor cells was calculated according to the width of the scratches.

Transwell assays were performed using the Corning Transwell kit (Corning Inc., One Riverfront Plaza Corning, New York, NY, USA). PANC-1 and SW-1990 cells were incubated in the upper chamber of a 24-well plate with 100 μL serum-free medium at 37 ˚C, 5% CO_2_ incubator for 1–2 h. Subsequently, 600 μL DMEM (Corning Inc., One Riverfront Plaza Corning, New York, NY, USA) containing 30% Ausbian FBS (Beijing Diyi Biology Science and Technology Co., Ltd., Beijing, China) was added to the bottom chamber. Then, all cells were incubated for 24 h, and tumor cells without metastasis were eliminated using a cotton swab. Cells that invaded through the membrane were fixed and stained, and photographs were taken using a fluorescence microscope (Olympus Co., Tokyo, Japan). The migration ability of the tumor cells was analyzed.

### 2.10. Co-Immunoprecipitation (Co-IP) Assay

Cell lysates from SW-1990 cells were quantified using the BCA Protein Assay kit (HyClone Co., Logan, UT, USA), and 1.0 mg total protein was incubated with anti-immunoglobulin G (IgG), anti-SNAI2, and anti-CDCA8 at 4 °C overnight. Then, 20 μL agarose beads were added to the cell lysates, which were incubated at 4 °C for 2 h. Subsequently, the cell lysates were incubated with IP cell lysis buffer and 5× loading buffer at 100 °C for 5 min. Finally, western blotting analysis was performed to analyze the protein samples.

### 2.11. Chromatin IP (ChIP)-qPCR and Dual-Luciferase Reporter Assay

ChIP-qPCR was carried out with SimpleChIP^®^ Enzymatic Chromatin IP Kit (Agarose Beads) #9002 (Cell signaling technology Inc., Danvers, MA, USA) according to the manufacturer’s instructions. SW-1990 cells were cross-linked, resuspended, and lysed. The DNA was then sheared into a range of 150–900 bp via ultrasonic waves. Then, chromatin fraction was immunoprecipitated using anti-SNAI2 or IgG antibodies. Subsequently, the complex was enriched with protein G agarose beads, which were isolated and washed. Finally, DNA was purified and subjected to qPCR analysis, as described in the qPCR assay section.

For the dual-luciferase reporter assay, a GL002 carrier (Promega Co., Commerce Park Dr, Madison, WI, USA) was used to establish a CD44 promoter dual-luciferase carrier. SW-1990 cells were cultured and co-transfected with pGL-CD44 wild type (WT) or pGL-CD44 mutant type (MUT) and LV-CDCA8, LV-SNAI2, LV-CDCA8+LV-SNAI2, or LV-Ctrl plasmids. All cells were washed, and the luciferase activities of the cell lysates were detected using a dual luciferase reporter assay kit (Vazyme Biotech Co., Ltd., Nanjing, China).

### 2.12. In Vivo Assay

All laboratory mice were 4-week-old female BALB/c nude mice (Beijing Vitalriver Experimental Animal Technology Co., Ltd., Beijing, China). SW-1990 cells, stably infected with shCtrl or shCDCA8, were subcutaneously injected into the right flank of mice with 0.2 mL (4 × 10^6^ cells/mice), and there were eight mice in each group (shCtrl and shCDCA8). After 2 weeks, the length (L) and width (W) of the tumors were recorded and tumor volume was calculated as π/6 × L × W2. All tumor parameters were collected and calculated 2–3 times weekly. The IVIS Spectrum Imaging System (PerkinElmer Chemagen Technology Inc., Baesweiler, Germany) was used to observe fluorescence images when all mice were sacrificed 35 days after the tumor cells were injected. Ultimately, Ki-67 immunostaining was carried out for tumor tissues from three mice in each group when they were executed. There were 3 slides in each tumor tissue, and four visual fields in each slide were scored for quantitative histological data (the staining index was described in “Human PDAC tissues” part). 

### 2.13. Statistical Analysis

All data are presented as frequencies for categorical variables and as mean ± standard deviation (S.D.) for continuous variables. Pearson 𝜒2 test or Fisher exact test was used to categorical variables with SPSS 22.0 (IBM Co., San Diego, CA, USA), and Student’s t-test or one way ANOVA was performed to compare the difference between two or multiple groups of continuous variables with GraphPad Prism 8.0 (GraphPad Software Inc., San Diego, CA, USA). The association between the expression level of CDCA8 and PDAC patient’s overall survival (OS) was investigated by the Kaplan–Meier analysis using SPSS 22.0 (IBM Co., San Diego, CA, USA). We carried out bioinformatics analysis, utilizing Spearman or Pearson test to analyse the correlation between two continuous variables via the R software package. Statistical significance was set at *p* < 0.05, and all tests were two-tailed.

## 3. Results

### 3.1. Relationship between the CDCA8 Expression and Demographic Characteristics of Patients with PDAC

**Figure 1:**
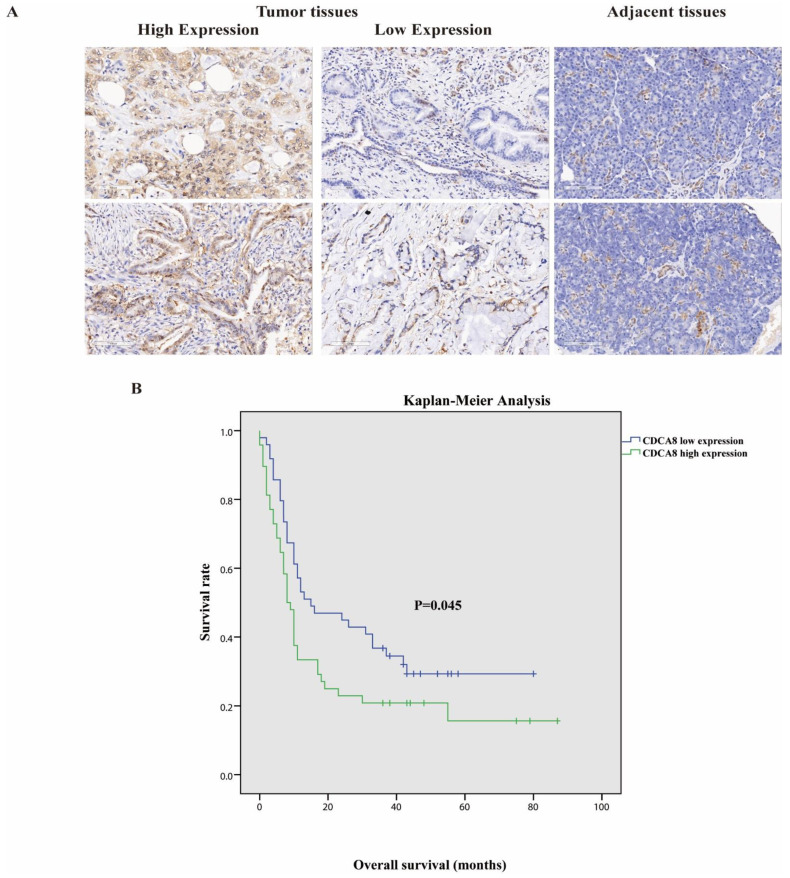
(**A**) Representative images of high and low CDCA8 expression in PDAC and adjacent tissues detected by immunohistochemistry (images with ×200 magnification, scale bar = 100 μm). (**B**) Overall survival had a significant difference between high and low CDCA8 expression of patients with PDAC. PDAC, pancreatic ductal adenocarcinoma.

Tumor tissues and corresponding adjacent tissues were collected from 97 patients with surgically treated PDAC. The expression of CDCA8 was significantly higher in tumor tissues, as detected by the IHC assay (Table 1, Figure 1A). They were then divided into two groups according to the staining intensity of CDCA8 in the tumor tissues: CDCA8 low- and high-expression groups (50.5% vs. 49.5%). The median cut-off value of the IHC score was six points. The expression of CDCA8 in PDAC was obviously correlated with tumor grade (*p* = 0.007) and history of diabetes (*p* = 0.030), whereas there was no significant difference between the two groups in other clinical characteristics, such as age, sex, and tumor stage (Table S1). Survival analysis of patients with PDAC was performed using the Kaplan–Meier analysis, showing that patients with low CDCA8 expression had a longer OS (CDCA8 low expression vs. high expression: 8 vs. 15 months, *p* = 0.045, Figure 1B).

### 3.2. Knockdown of CDCA8 Inhibited the Proliferation and Migration of PDAC In Vitro

**Figure 2:**
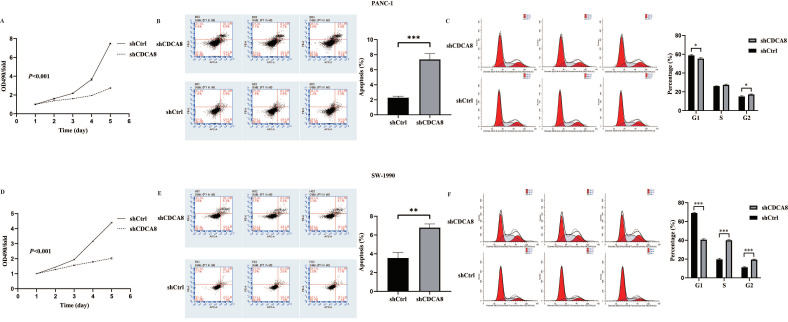
CDCA8 promoted the proliferation of PDAC in vitro. (**A**,**D**) The MTT assay showed proliferation rate of PANC-1 and SW-1990 cells transfected with shCtrl and shCDCA8 plasmids. (**B**,**E**) The cell apoptosis rate was detected via Annexin V-APC/PI staining flow in PANC-1 and SW-1990 cells infected with shCtrl and shCDCA8 plasmids. (**C**,**F**) Cell cycles were assessed via flow cytometry assays in PANC-1 and SW-1990 cells infected with shCtrl and shCDCA8 plasmids. Histograms are presented as mean ± SEM. PDAC, pancreatic ductal adenocarcinoma; MTT, 3-(4,5-Dimethylthiazol-2-yl) -2,5-diphenyltetrazolium bromide; PI, phosphatidylserine; shRNA, short hairpin RNA; SEM, standard error of the mean. * *p* < 0.05, ** *p* < 0.01, *** *p* < 0.001.

Based on the close relationship between the high CDCA8 expression and poor prognosis of patients with PDAC, and a previous study suggesting that KIF18B promotes the progression of PDAC by upregulating CDCA8 [18], we hypothesized that CDCA8 could promote PDAC progression. To confirm this hypothesis, the expression of CDCA8 was knocked down using LV-shCDCA8 in the PANC-1 and SW-1990 cell lines. The results suggested that the expression level of CDCA8 was significantly decreased in the shCDCA8 group of PANC-1 and SW-1990 cells via qPCR and western blotting (Figure S1D–G). To test the effects of CDCA8 on PDAC proliferation, an MTT assay was performed in PANC-1 and SW-1990 cells. The shCDCA8 group showed a significantly lower proliferation rate (Figure 2A,D). 

Differences in cell cycle and apoptosis of tumor cells between the shCDCA8 and control groups were observed using flow cytometry in both PANC-1 and SW-1990 cell lines. The results demonstrated that CDCA8 knockdown significantly increased the apoptotic percentage of both PANC-1 and SW-1990 cells (Figure 2B,E). However, in the shCDCA8 group, the percentage of cells decreased in the G1 phase and increased in the G2 phase (Figure 2C,F). 

**Figure 3:**
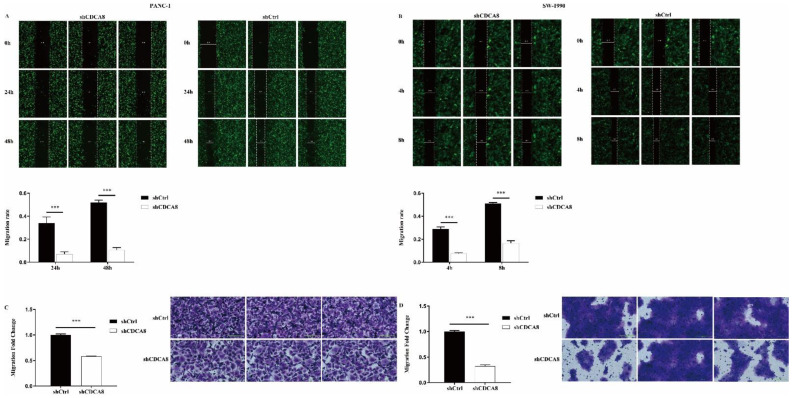
CDCA8 promoted the invasion of PDAC *in vitro*. (**A**,**B**) Wound-healing assays were performed in PANC-1 and SW-1990 cells infected with shCtrl and shCDCA8 plasmids. Results exhibited the migration rate of PANC-1 cells in 0 h, 24 h, and 48 h and of SW-1990 cells in 0 h, 4 h, and 8 h. (**C**,**D**) Transwell assays were carried out in PANC-1 and SW-1990 cells infected with shCtrl and shCDCA8 plasmids and representative images are shown (images with ×200 magnification, scale bar = 200 μm). Histograms are presented as mean ± SEM. PDAC, pancreatic ductal adenocarcinoma; shRNA, short hairpin RNA; SEM, standard error of the mean. *** *p* < 0.001.

We also compared the difference in the migration ability and rate of tumor cells between the shCDCA8 and control groups in both PANC-1 and SW-1990 cells via wound healing and transwell assays. Data showed that CDCA8 knockdown significantly inhibited the wound healing rate (Figure 3A,B) and migration ability (Figure 3C,D) of both PANC-1 and SW-1990 cells.

In summary, CDCA8 knockdown inhibits the proliferation and migration of PDAC cells.

### 3.3. CDCA8 Silencing Reduced the Tumour Growth in Xenograft Models

**Figure 4:**
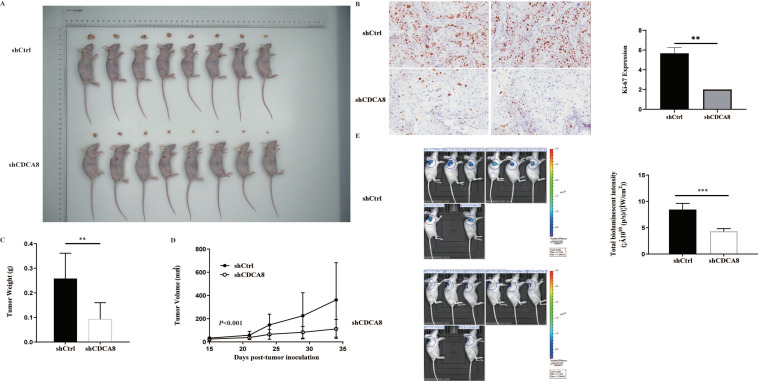
CDCA8 promoted the progression of PDAC in vivo. (**A**) Representative photographs of tumors in nude mice formed by SW-1990 cells infected with shCtrl or shCDCA8 plasmids (*n* = 8 in each group). (**B**) Representative images of tumors in nude mice detected by IHC staining were shown (images with ×200 magnification, scale bar = 100 μm). Ki-67 was measured and compared between the shCtrl and shCDCA8 groups. (**C**) Tumor weight was measured when nude mice were euthanized. (**D**) Tumor volume was measured and growth curves were drawn. (**E**) Tumors in nude mice were evaluated via an in vivo imaging system and intensity of total bioluminescent was measured. Histogram and line chart are presented as mean ± SEM. IHC, immunohistochemistry; PDAC, pancreatic ductal adenocarcinoma; shRNA, short hairpin RNA; SEM, standard error of the mean. ** *p* < 0.01, *** *p* < 0.001.

In vitro experiments confirmed that CDCA8 plays a critical role in the proliferation and migration of PDAC cells. To further explore whether CDCA8 contributed to PDAC growth in vivo, an in vivo experiment was conducted. SW-1990 cells infected with shCDCA8 or shCtrl were injected into the nude mice. After 14 days, the tumors were removed from the nude mice. The volume and weight of the tumors were measured weekly. Eight tumors of mice from the control and shCDCA8 groups were photographed and shown in Figure 4A. The differences of tumor weight and volume between the two groups are shown in Figure 4C,D, indicating that tumors in the shCDCA8 group were smaller than those in the control group.

To further confirm these results, we examined Ki-67 expression using IHC staining. Tumors in the shCDCA8 group had a lower proliferation rate. We then performed in vivo imaging in both groups, with representative images shown (Figure 4B). Compared to the control group, the total bioluminescent intensity of the shCDCA8 group was significantly lower, indicating that tumors in the shCDCA8 group had lower activity (Figure 4E). 

In conclusion, we discovered that CDCA8 silencing inhibited the proliferation of pancreatic cancer cells in vivo.

### 3.4. CDCA8/CD44 Axis Promoted Cancer Cell Proliferation and Migration

A previous study demonstrated that CD44, a major hyaluronan receptor, plays an important role in metastatic PDAC [13]. Given that CDCA8 promoted the proliferation and migration of pancreatic cancer cells in vitro and in vivo, we next sought to explore the underlying mechanisms by focusing on the relationship between CDCA8 and CD44 expression. A correlation between CDCA8 and CD44 was found through ingenuity pathway analysis (Figure S2A). We analyze the correlation between CDCA8 and CD44 using RNA-seq data and the corresponding clinical information of 179 patients with PDAC from The Cancer Genome Atlas (TCGA) dataset (https://portal.gdc.com (accessed on 7 July 2022)). A significant positive correlation was observed between CDCA8 and CD44 (*p* < 0.001, Figure S2B). To verify the accuracy of the above results, we combined GSE16515, GSE2873, and GSE62452 datasets from the Gene Expression Omnibus (GEO) data-set and removed the batch effect to obtain 189 tumor samples. We determined that CDAC8 was positively correlated with CD44 (*p* < 0.001, Figure S2C).

Subsequently, we performed qPCR and western blotting to detect the expression levels of CDCA8 and CD44 in PANC-1 cells, which was consistent with our hypothesis that the knockdown of CDCA8 could significantly decrease the expression of CD44 (Figure S2D,E). We detected the expression of CD44 in HPDE6-C7, SW-1990, PANC-1, and BXPC-3 cell lines. Because of the higher expression levels of CD44, SW-1990, and PANC-1 cell lines were selected for subsequent experiments. (Figure S3A)

**Figure 5:**
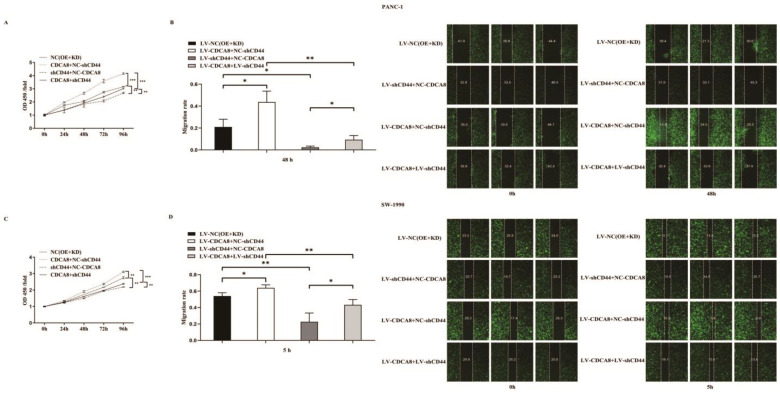
CDCA8-CD44 axis promoted the proliferation and invasion of PDAC in vitro. (**A**,**C**) CCK-8 was performed in SW-1990 cells infected with LV-NC, LV-CDCA8+NC-shCtrl, NC-CDCA8+shCD44, or LV-CDCA8+shCD44 plasmids, which showed that the inhibition of tumor cells proliferation resulted from CD44 knockdown could be rescued by CDCA8 overexpression. (**B**,**D**) Wound-healing assays were performed in PANC-1 and SW-1990 cells infected with LV-NC, LV-CDCA8+NC-shCtrl, NC-CDCA8+shCD44, or LV-CDCA8+shCD44 plasmids. Results exhibited the migration rate in 0 h and 48 h of PANC-1 cells and 0 h and 5 h of SW-1990 cells. The results exhibited the inhibition of tumor cells migration resulted from CD44 knockdown could be rescued by CDCA8 overexpression. Histogram and line chart are presented as mean ± SEM. NC, normal contrast; PDAC, pancreatic ductal adenocarcinoma; CCK-8, cell counting Kit-8; shRNA, short hairpin RNA; SEM, standard error of the mean. * *p* < 0.05, ** *p* < 0.01, *** *p* < 0.001.

To confirm whether CDCA8 promotes the proliferation of PDAC by activating the expression of CD44, we performed a CCK8 assay in PANC-1 and SW-1990 cell lines. According to the results, tumor cells overexpressing CDCA8 had the highest proliferation capacity, whereas tumor cells with knockdown of CD44 had the lowest proliferation capacity. However, CDCA8 overexpression offset the low proliferation rate of tumor cells caused by the knockdown of CD44 (Figure 5A,C). Migration ability of PANC-1 and SW-1990 cells was the lowest in the shCD44+NC-CDCA8 group using the wound-healing assay and CDCA8 overexpression offset the difference resulted from the CD44 knockdown (Figure 5B,D).

In summary, our results suggest that CDCA8 promotes the proliferation and migration of PDAC cells by activating the expression of the downstream molecule CD44.

### 3.5. CDCA8/SNAI2 Complex Up-Regulate CD44 through Integrating with the Promoter of CD44

**Figure 6:**
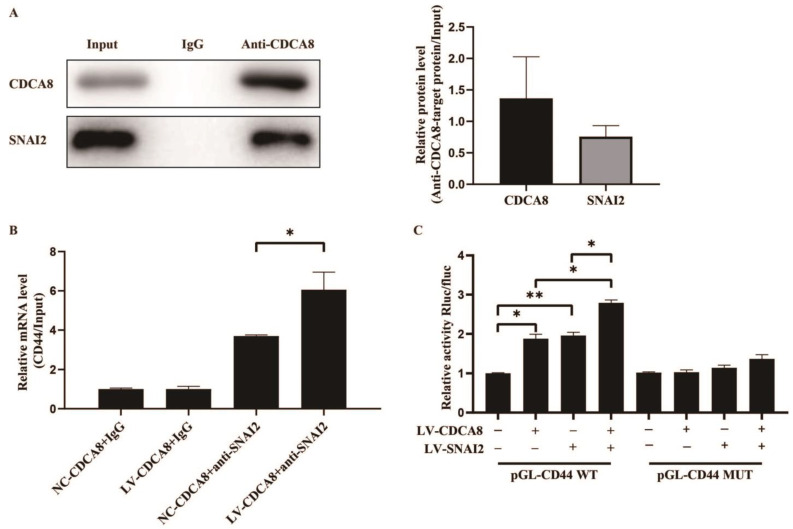
CDCA8/SNAI2 complex up-regulates CD44 via integrating with the promoter of CD44 in vitro. (**A**) CO-IP assay was performed in SW-1990 cells and SNAI2 could be detected through the anti-CDCA8 antibody. (**B**) The ChIP-qPCR assay was performed in SW-1990 cells transfected with NC-CDCA8 or LV-CDCA8. The CD44 promoter fragment was enriched via the IgG or anti-SNAI2 antibody. (**C**) Luciferase activity of pGL-CD44 WT and pGL-CD44 MUT in SW-1990 cells co-transfected with LV-CDCA8, LV-SNAI2, or LV-CDCA8+SNAI2 plasmids was analyzed by dual-luciferase reporter assay. Histograms are presented as mean ± SEM. WT, wild type; MUT, mutant type; CO-IP, co-immunoprecipitation; ChIP-qPCR, chromatin immunoprecipitation-real-time quantitative polymerase chain reaction; IgG, immunoglobulin G; SNAI2, snail family transcriptional repressor 2; shRNA, short hairpin RNA; SEM, standard error of the mean. * *p* < 0.05, ** *p* < 0.01.

To further explore the molecular mechanism between CDCA8 and CD44, we searched for human transcription factors for CD44 via the TRRUST v2 (Transcriptional Regulatory Relationships Unraveled by Sentence-based Text Mining) database and focused on a potential intermediate molecule between CDCA8 and CD44—SNAI2 (Table S3). We then carried out the CO-IP assay in the SW-1990 cell line, which showed that the CDCA8 antibody was specifically co-immunoprecipitated by SNAI2 in contrast to the IgG antibody, indicating that CDCA8 could be combined with SNAI2 as CDCA8/SNAI2 complex to work. (Figure 6A) 

Subsequently, a CHIP-qPCR assay was conducted in the SW-1990 cell line to explore whether CDCA8 could transcriptionally regulate the expression of CD44 via CDCA8/SNAI2 complex. The data showed that the overexpression of CDCA8 could increase the binding of the CDCA8/SNAI2 complex with the CD44 promoter (Figure 6B). To confirm this conclusion, a dual-luciferase reporter assay was performed and the pGL-CD44 plasmid containing the CD44 promoter region, including the WT and MUT, was co-transfected with LV-CDCA8, LV-SNAI2, and control plasmid. The data showed that CDCA8 and SNAI2 promoted the activation of the CD44 promoter in SW-1990 cells (Figure 6C).

In summary, these results clarify that CDCA8 transcriptionally promotes the expression of CD44 via the intermediate CDCA8/SNAI2 complex.

## 4. Discussion

As one of the most common malignant tumors in humans, the 5-year survival rate of patients with PDAC has increased from < 5% to 8% over the past few decades, while there has been a great improvement in the survival rate of other tumors [2,18]. Although surgical resection provides the only opportunity to cure this disease, most patients have to accept non-surgical treatments, such as chemotherapy and radiotherapy, owing to the low early diagnosis and radical resection rates [19,20]. To increase the radical resection rate, neoadjuvant chemotherapy has gradually become the main treatment for patients with locally advanced or unresectable PDAC [21,22]. However, there is a great difference in successful rates of treatment conversion among various studies [23,24]. Thus, targeted therapy remains the most promising treatment for PDAC in the future, but the lack of effective target genes limits the development of targeted therapies [25]. Therefore, there is an urgent need to identify new targets for the targeted therapy of PDAC. 

**Figure 7:**
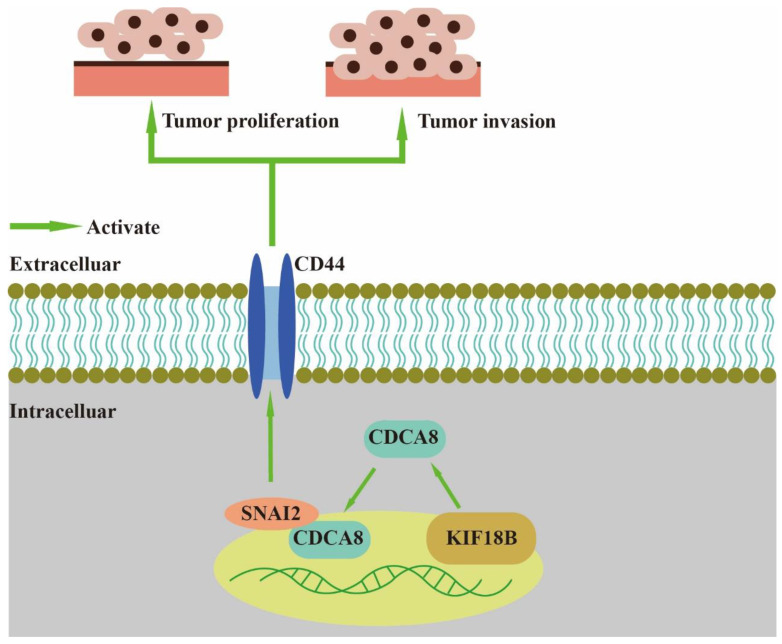
CDCA8/SNAI2-CD44 axis promotes the proliferation and invasion of PDAC.

In our study, we found that CDCA8, an essential regulator of mitosis and cell division, could up-regulate the expression of CD44 to promote proliferation and invasion of PDAC when combined with SNAI2 (Figure 7). As a member of the CPC family, CDCA8 is usually expressed in hESCs. However, previous studies have shown that CDCA8 can also be expressed in tumor cells, including breast, bladder, and lung cancers [10]. CDCA8 has been previously shown to promote pancreatic cancer cell proliferation and invasion [26]. In this study, we also found that there was overexpression of CDCA8 in PDAC tumor tissues compared to that in adjacent tissues. Furthermore, analysis of the demographic characteristics of patients with PDAC showed that CDCA8 expression was positively associated with tumor grade. The depletion of CDCA8 delays mitotic progression, resulting in kinetochore-spindle mis-attachments and an increase in bipolar spindles associated with ectopic asters. Therefore, interference with CDCA8 may have a great impact on the regulation of the cell cycle and cell proliferation [27]. Interestingly, in our study, we discovered that silencing CDCA8 decreased the percentage of cells in the G1 phase but increased the percentage of cells in the G2 phase of cell cycle. In addition, we found that knockdown of CDCA8 promoted the apoptosis of tumor cells. The explanation for this phenomenon is that CDCA8 downregulation blocked the cell cycle in the G2 phase, and the G2-damage checkpoint prevented the cell cycle from entering the M phase [28], resulting in apoptosis and a low rate of tumor cell proliferation. Through subsequent in vivo and in vitro assays, it was found that the knockdown of CDCA8 could also decrease the migration ability of tumor cells, consistent with previous studies and our clinical findings. The apoptosis of tumor cells decreased some secretory factors that stimulated the migration of tumor cells, which might have affected wound healing and transwell assays to some extent. Furthermore, it was discovered that CDCA8 promoted the proliferation and invasion of PDAC via transcriptional regulation of CD44, a transmembrane receptor participating in the progression and metastasis of tumor cells [14]. To explore the specific molecular mechanism of CDCA8 regulating CD44, a CO-IP assay was carried out to confirm that CDCA8 could be combined with a transcription factor of CD44, called SNAI2, to form the CDCA8/SNAI2 complex. CHIP-qPCR and dual-luciferase reporter assays showed that CDCA8/SNAI2 could integrate with the CD44 promoter to up-regulate transcription. 

Recently, an increasing number of studies have focused on CDCA8 [29]. Several human cancers, including lung cancer, bladder cancer, and hepatocellular carcinoma, have reported that CDCA8 could serve as a poor prognostic marker [30,31]. A previous study discovered that CDCA8 could inhibit the ATF3 tumor suppressor and activate the AKT/β-catenin signalling pathway to promote the development of hepatocellular carcinoma [32]. In addition, miR-133a/133b have been found to act as suppressors of CDCA8 [33,34]. CD44, a cell adhesion receptor, not only plays a key role in tumor cell metastasis, but also has a great impact on regulating epithelial–mesenchymal plasticity and stemness of cancer stem cells [35]. Hyaluronic acid-mediated signalling of CD44 associated with tumor metastasis have been widely studied [14]. A recent study showed that downregulating the HIF-1α/NOTCH-1 pathway was able to eliminate CD44^+^ cancer stem-like cell phenotypes to enhance the malignancy and chemo-resistance in neck squamous cell carcinomas [36]. A previous study has clarified that KIF-18B was involved in the growth and progression of tumor cells by up-regulating transcription of CDCA8 [26]. Although the upstream molecule of CDCA8 has been identified, the downstream signalling pathway remains unclear [37]. In our study, we found that the poor prognosis of patients with PDAC was positively correlated with the expression of CDCA8 in human samples. Consequently, CDCA8 could up-regulate CD44 transcription through the CDCA8/SNAI2 complex to promote PDAC progression. 

In our in vivo assays, we discovered that CDCA8 knockdown significantly inhibited the tumor volume, weight, and Ki-67 index of mouse models, indicating that CDCA8 plays a vital role in promoting the growth of PDAC. Previous studies have shown that KIF18B promotes pancreatic cancer cell proliferation by activating CDCA8 in in vitro assays. Our study further supports this viewpoint using in vivo assays. However, there were some limitations to our mouse model. The cells which were implanted subcutaneously into mouse models were not patient-derived tumor cells; therefore, they were unable to completely exhibit the biological behavior of human pancreatic cancer cells. Furthermore, these mouse models could not imitate the development of human pancreatic cancer, unlike genetically engineered mouse models.

## 5. Conclusions

In summary, the CDCA8/CD44 signalling pathway contributes to the proliferation and invasion of PDAC through the CDCA8/SNAI2 complex integrated with CD44 promoter. Our study suggests that CDCA8 may be a promising therapeutic target for PDAC.

## Figures and Tables

**Table 1:** Immunohistochemistry analysis of CDCA8 expression in pancreatic cancer tissues and adjacent tissues.

CDCA8 Expression	Tumor Tissue	Adjacent Tissue	*p* Value
Low	49 (50.5%)	57 (85.1%)	<0.001
High	48 (49.5%)	10 (14.9%)	

## Data Availability

The data presented in this study are available on request from the corresponding author.

## Supplementary Materials

The following supporting information can be downloaded at: https://www.mdpi.com/article/10.3390/cancers14215434/s1, Figure S1: The efficiency of CDCA8 knockdown was detected, and western blotting and qPCR were utilized to detect the expression; Figure S2: IPA, bioinformatics analysis western blotting, and qPCR were performed to explore the potential downstream molecule of the relationship between CD44 and CDCA8; Figure S3: The efficiency of CDCA8 overexpression and CD44 knockdown was detected, and western blotting and qPCR were utilized to detect the expression; Figure S4. Two different RNAi sequences of CDCA8 (RNAi-10540 [shCDCA8-2], RNAi-10540 [shCDCA8-3]) were used to perform MTT and apoptosis assays; Figure S5. Two different RNAi sequences of CDCA8 (RNAi-10540 [shCDCA8-2], RNAi-10540 [shCDCA8-3]) were used to perform transwell and wound-healing assays; Figure S6. The original Western Blotting figures; Table S1: Relationship between CDCA8 expression and demographic characteristics of patients with pancreatic cancer; Table S2: The sequences of designed primer; Table S3: Transcript factors for CD44.
