# Supplementary material for: CDCA8/SNAI2 Complex Activates CD44 to Promote Proliferation and Invasion of Pancreatic Ductal Adenocarcinoma
Source: Cancers (Basel). 2022 Nov 4;14(21):5434. doi: 10.3390/cancers14215434 (PMC9657053; doi:10.3390/cancers14215434)
Supplement: Supplementary file 1 [file cancers-14-05434-s001.zip › cancers-1962670-supplementary-send to xml.pdf]

cancers

MDPI

Article

CDCA8/SNAI2 Complex Activates CD44 to Promote Proliferation and Invasion of Pancreatic Ductal Adenocarcinoma

Jichun Gu, Yujie Guo, Jiali Du, Lei Kong, Junyuan Deng, Baian Tao, Hengchao Li, Chen Jin, Deliang Fu and Ji Li

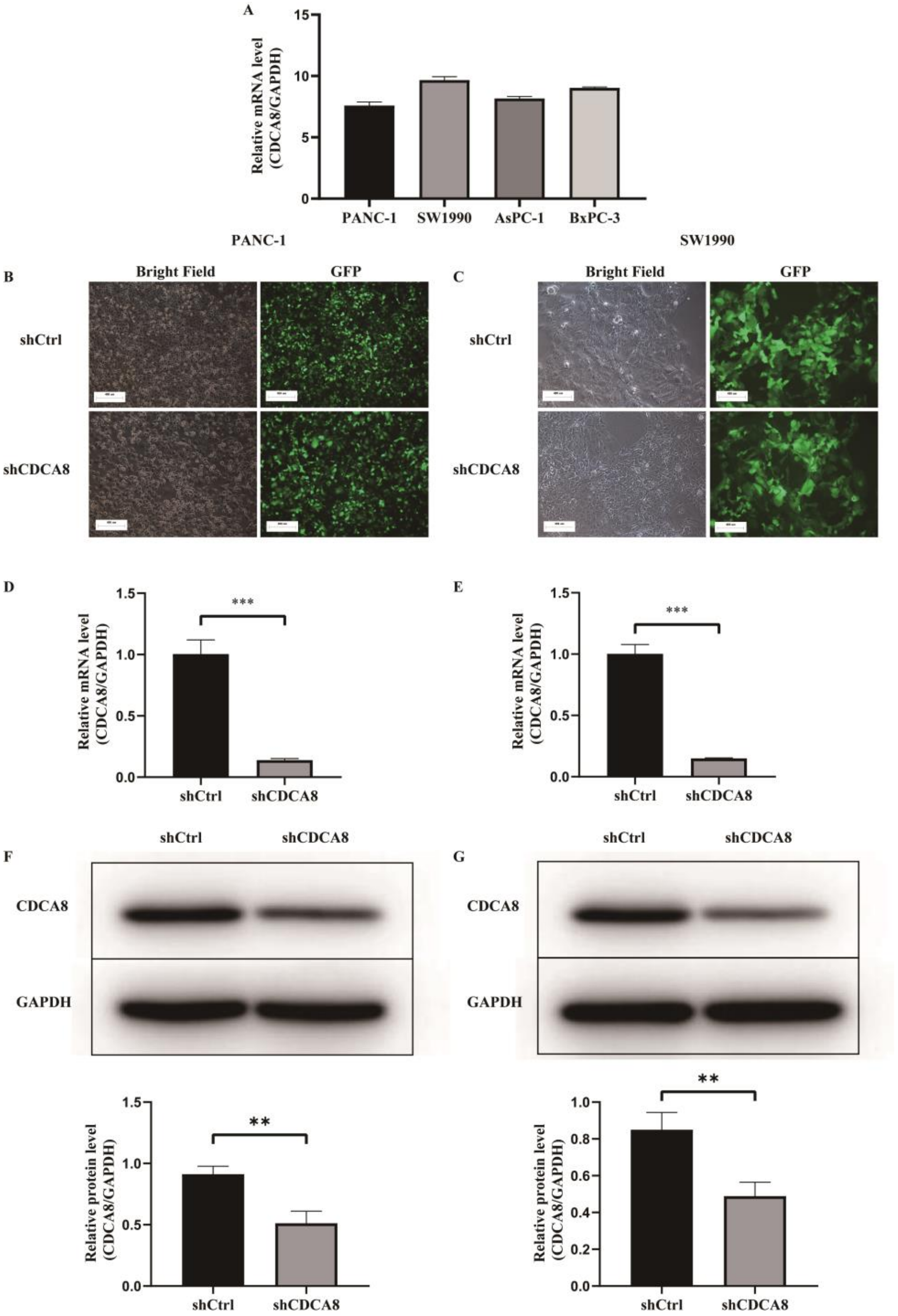

Figure S1. The efficiency of CDCA8 knockdown was monitored by western blotting and qPCR was utilized to detect the expression. (**A**) The mRNA level of CDCA8 was assayed via qPCR in PANC-

1, SW-1990, AsPC-1 and BxPC-3 cells (**B, C**) PANC-1 and SW-1990 cells were infected with shCtrl or shCDCA8 for 72h with a >80% efficiency of infection (images with ×100 magnification, scale bar = 400μm). (D, E) qPCR showed that CDCA8 was knocked down significantly in shCDCA8 group of PANC-1 and SW-1990 cells. (F, G) Western blotting showed that the expression of CDCA8 in shCDCA8 group was down-regulated in PANC-1 and SW-1990 cells. Histograms are presented as mean ± SEM. qRCP, real-time quantitative polymerase chain reaction; shRNA, short hairpin RNA; SEM, standard error of the mean. ** $P < 0.01$, *** $P < 0.001$.

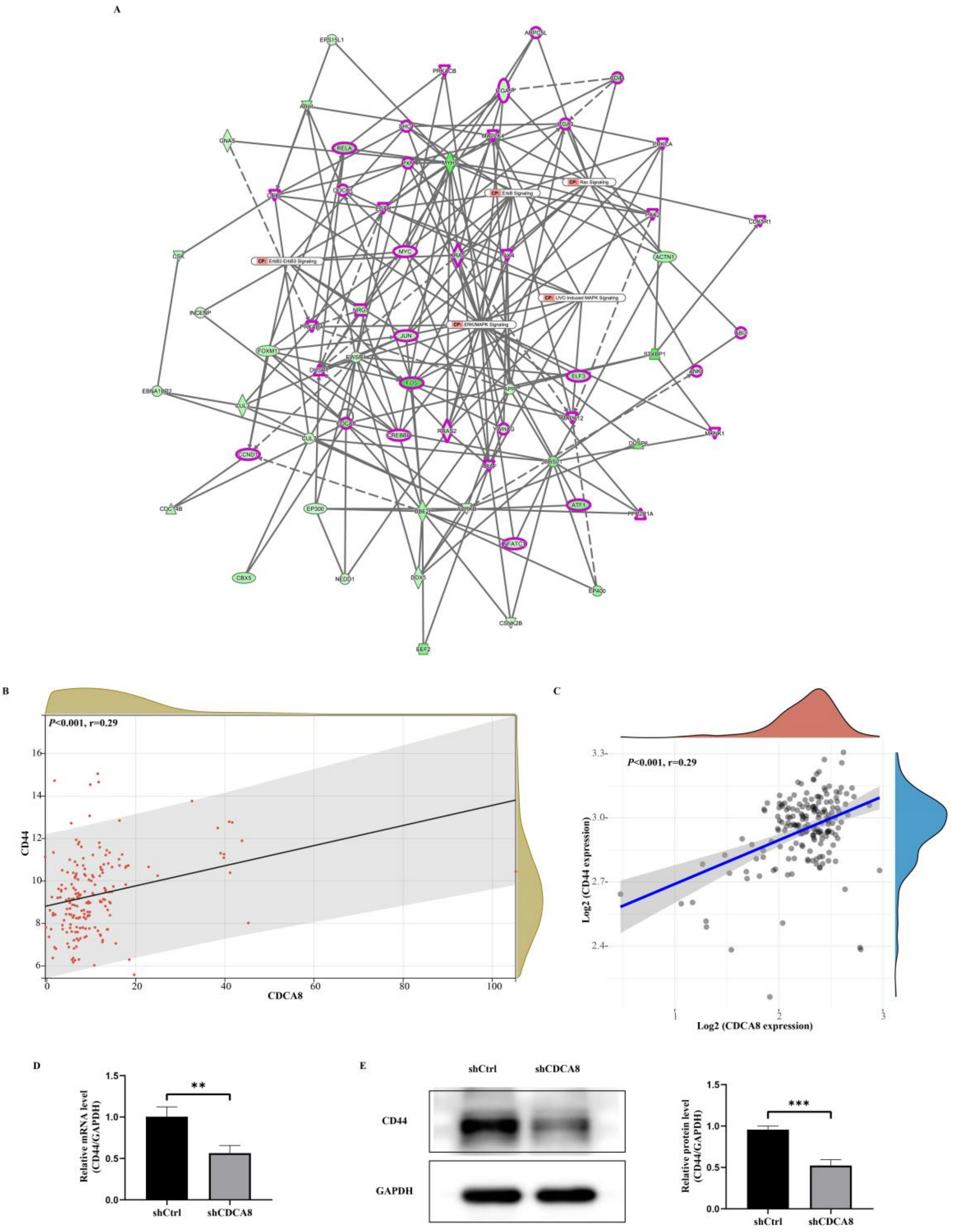

Figure S2. IPA, bioinformatics analysis western blotting and qPCR were performed to explore the relationship between CD44 and CDCA8. (**A**) IPA was carried out to explore the potential downstream molecule for CDCA8. (**B, C**) Bioinformatics analysis was performed to analyze the correlation between CDCA8 and CD44 in TCGA and GEO data set. (**D**) mRNA level of CD44 was assayed

via qPCR in PANC-1 cells transfected with shCDCA8. (**E**) Protein level of CD44 was analyzed via Western blotting in PANC-1 cells transfected with shCDCA8. Histogram is presented as mean ± SEM. TCGA, The Cancer Genome Atlas; GEO, Gene Expression Omnibus; qPCR, real-time quantitative polymerase chain reaction; SEM, standard error of the mean. ** *P* < 0.01, *** *P* < 0.001.

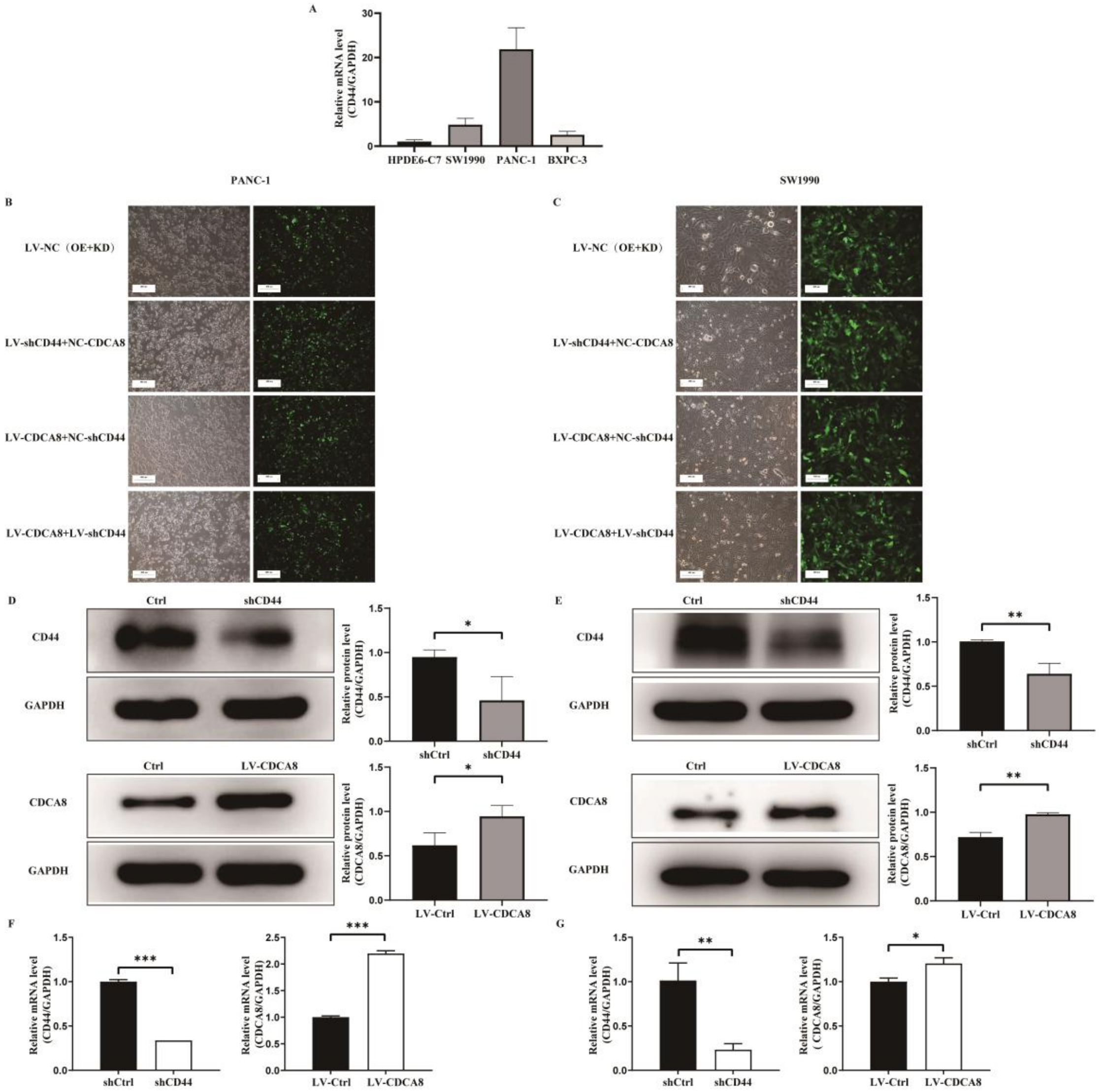

Figure S3. The efficiency of CDCA8 overexpression and CD44 knockdown was detected and western blotting and qPCR was utilized to detect the expression. (**A**) The mRNA level of CDCA8 was detected via qPCR in PANC-1, SW-1990, HPDE6-C7 and BxPC-3 cells (**B, C**) PANC-1 and SW-1990 cells were infected with LV-NC, LV-CDCA8+NC-shCtrl, NC-CDCA8+shCD44 or LV-CDCA8+shCD44 plasmids for 72h with a >80% efficiency of infection (images with ×100 magnification, scale bar = 400μm). (**D, E**) Protein level of PANC-1 and SW-1990 cells infected with LV-CDCA8 or shCD44 was detected via western blotting. (**F, G**) mRNA level of PANC-1 and SW-1990 cells infected with LV-CDCA8 or shCD44 was detected via qPCR. Histograms are presented as mean ± SEM. qRCP, real-time quantitative polymerase chain reaction; shRNA, short hairpin RNA; SEM, standard error of the mean. * *P* < 0.05, ** *P* < 0.01, *** *P*< 0.001.

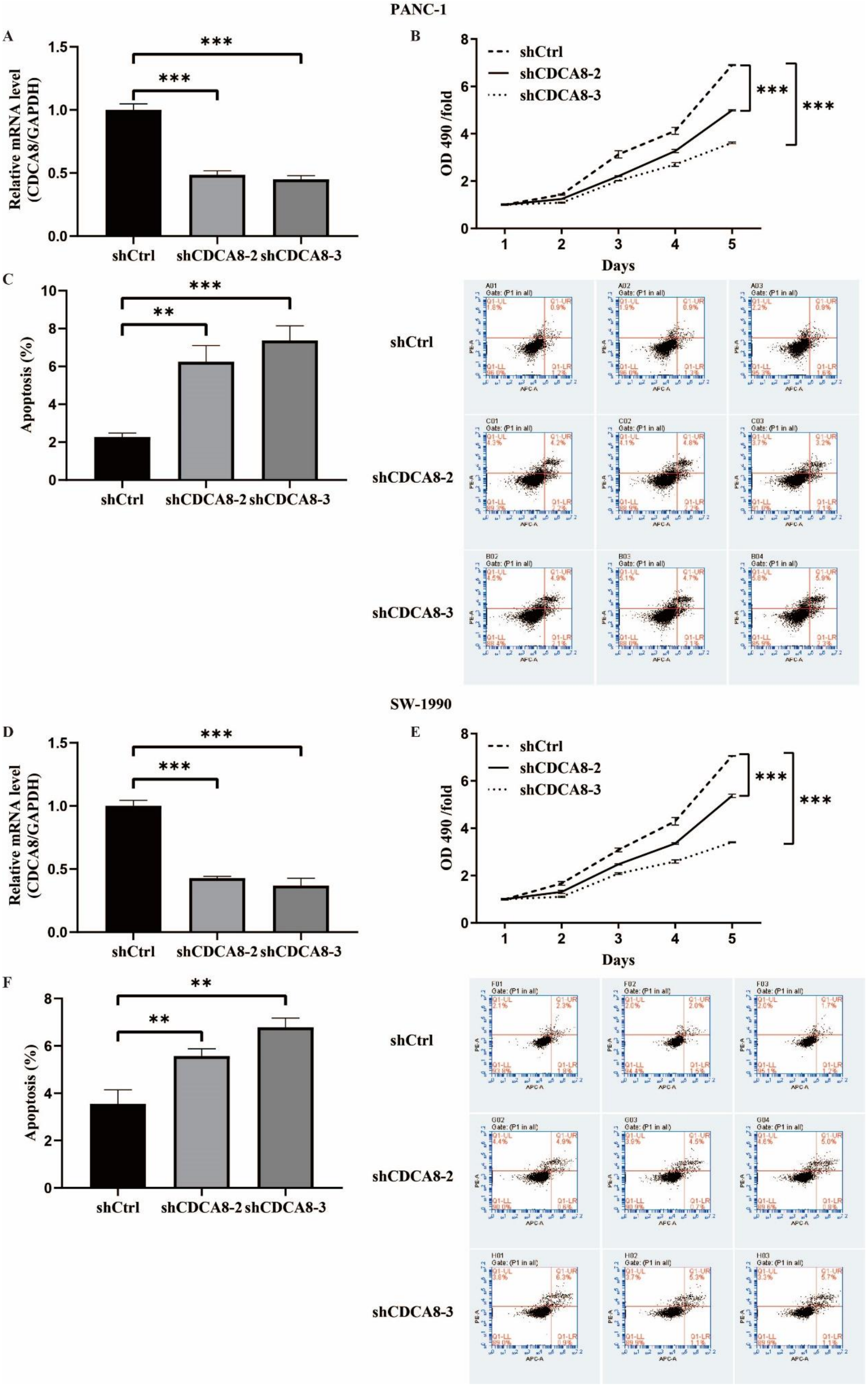

Figure S4. Two different RNAi sequences of CDCA8 (RNAi-10540 [shCDCA8-2], RNAi-10540 [shCDCA8-3]) were used to perform MTT and apoptosis assays. (**A, D**) The mRNA level of CDCA8

was detected via qPCR in PANC-1 and SW-1990 transfected with shCtrl, shCDCA8-2 and shCDCA8-3. (**B, E**) The MTT assay showed proliferation rate of PANC-1 and SW-1990 cells transfected with shCtrl, shCDCA8-2 and shCDCA8-3 plasmids. (**C, F**) The cell apoptosis rate was detected via Annexin V-APC/PI staining flow in PANC-1 and SW-1990 cells infected with shCtrl, shCDCA8-2 and shCDCA8-3 plasmids. Line charts are presented as mean ± SEM. qRCP, real-time quantitative polymerase chain reaction; MTT, 3-(4,5-Dimethylthiazol-2-yl) -2,5-diphenyltetrazolium bromide; PI, phosphatidylserine; shRNA, short hairpin RNA; SEM, standard error of the mean. ***P* < 0.01, *** *P* < 0.001.

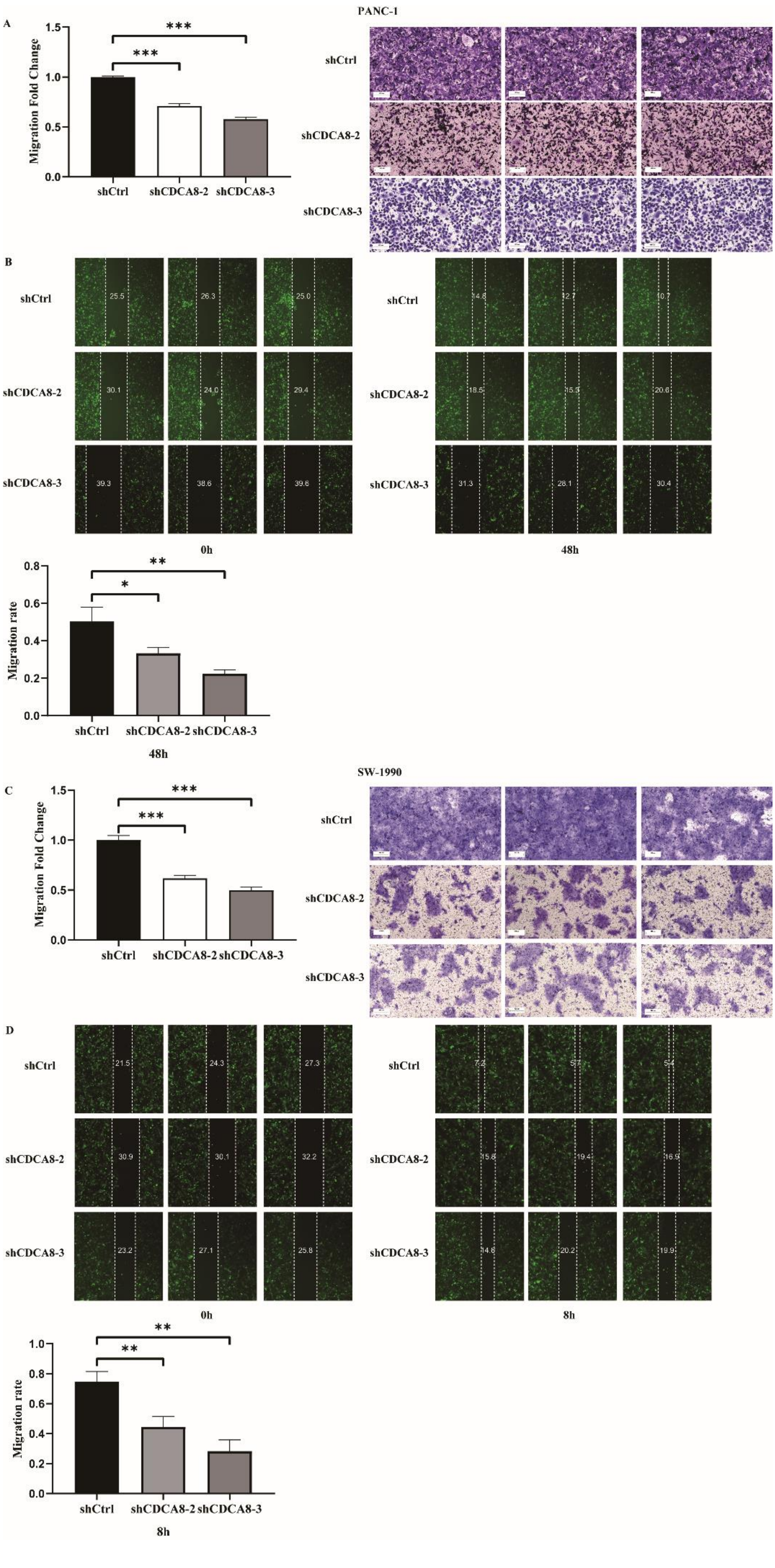
A
PANC-1
Migration Fold Change
1.5
1.0
0.5
0.0
***
***
shCtrl
shCDCA8-2
shCDCA8-3
B
shCtrl
shCDCA8-2
shCDCA8-3
25.5
26.3
25.0
30.1
24.0
29.4
39.3
38.6
39.6
0h
14.8
12.7
10.7
18.5
15.5
20.6
31.3
28.1
30.4
48h
Migration rate
0.8
0.6
0.4
0.2
0.0
**
*
48h
C
SW-1990
D
21.5
24.3
27.3
30.9
30.1
32.2
23.2
27.1
25.8
0h
7.2
5.7
5.4
15.8
19.4
16.9
14.8
20.2
19.9
8h
1.0
8h

Figure S5. Two different RNAi sequences of CDCA8 (RNAi-10540 [shCDCA8-2], RNAi-10540 [shCDCA8-3]) were used to perform transwell and wound-healing assays. (**A, C**) Transwell assays were carried out in PANC-1 and SW-1990 cells infected with shCtrl, shCDCA8-2 and shCDCA8-3 plasmids. Representative images are shown (images with ×100 magnification, scale bar = 400μm). (**B, D**) Wound-healing assays were performed in PANC-1 and SW-1990 cells infected with shCtrl, shCDCA8-2 and shCDCA8-3 plasmids. Results exhibited the migration rate of PANC-1 cells in 0h and 48h and of SW-1990 cells in 0h and 8h. Histograms are presented as mean ± SEM. shRNA, short hairpin RNA; SEM, standard error of the mean. $^{*}P < 0.05$, $^{**}P < 0.01$, $^{***}P < 0.001$.

Table S1. Relationship between CDCA8 expression and demographic characteristics of patients with PDAC.

Variable, n	No. of patients	CDCA8 expression		*P* value
		low	high	
	N = 97	*N* = 49	*N* = 48	
Age				0.223
≤59	48	21	27	
>59	48	27	21	
Sex				0.620
Male	61	32	29	
Female	36	17	19	
Lymph node positive				0.681
No	47	25	22	
Yes	41	20	21	
Tumor size				0.835
≤4cm	59	30	29	
>4cm	37	18	19	
Tumor Grade				0.007
II	65	39	26	
III	31	10	21	
IV	1	0	1	
Stage				0.207
I	37	21	16	
II-III	55	26	29	
IV	2	0	2	
History of diabetes				0.030
No	5	5	0	
Yes	61	30	31	

Table S2. The sequences of designed primer.

Target gene	Upstream primer sequence	Downstream primer sequence	Amplified fragment size (bp)
CDCA8	TTGAGTCAGACAGGCAGAACC	TTCCTCCAAGGGCGAAGTAG	118
CD44	TGGGTTCATAGAAGGGCACG	ATACTGGGAGGTGTTGGATGTG	106
GAPDH	TGACTTCAACAGCGACACCCA	CACCCTGTTGCTGTAGCCAAA	121

Table S3. Transcript factors for CD44.

Transcript Factors	Target Gene	Type
CTNNB1	CD44	Unknown
HDAC1	CD44	Repression
HMGA1	CD44	Activation

IKBKB	CD44	Repression
MYCN	CD44	Activation
SMARCA1	CD44	Repression
SMARCA4	CD44	Repression
SMARCB1	CD44	Repression
SNAI2	CD44	Activation
SP1	CD44	Activation
TCF4	CD44	Unknown
TWIST1	CD44	Activation
TWIST2	CD44	Activation
