# Supplementary figures and images for: CDCA8/SNAI2 Complex Activates CD44 to Promote Proliferation and Invasion of Pancreatic Ductal Adenocarcinoma
Source: Cancers (Basel). 2022 Nov 4;14(21):5434. doi: 10.3390/cancers14215434 (PMC9657053; doi:10.3390/cancers14215434)

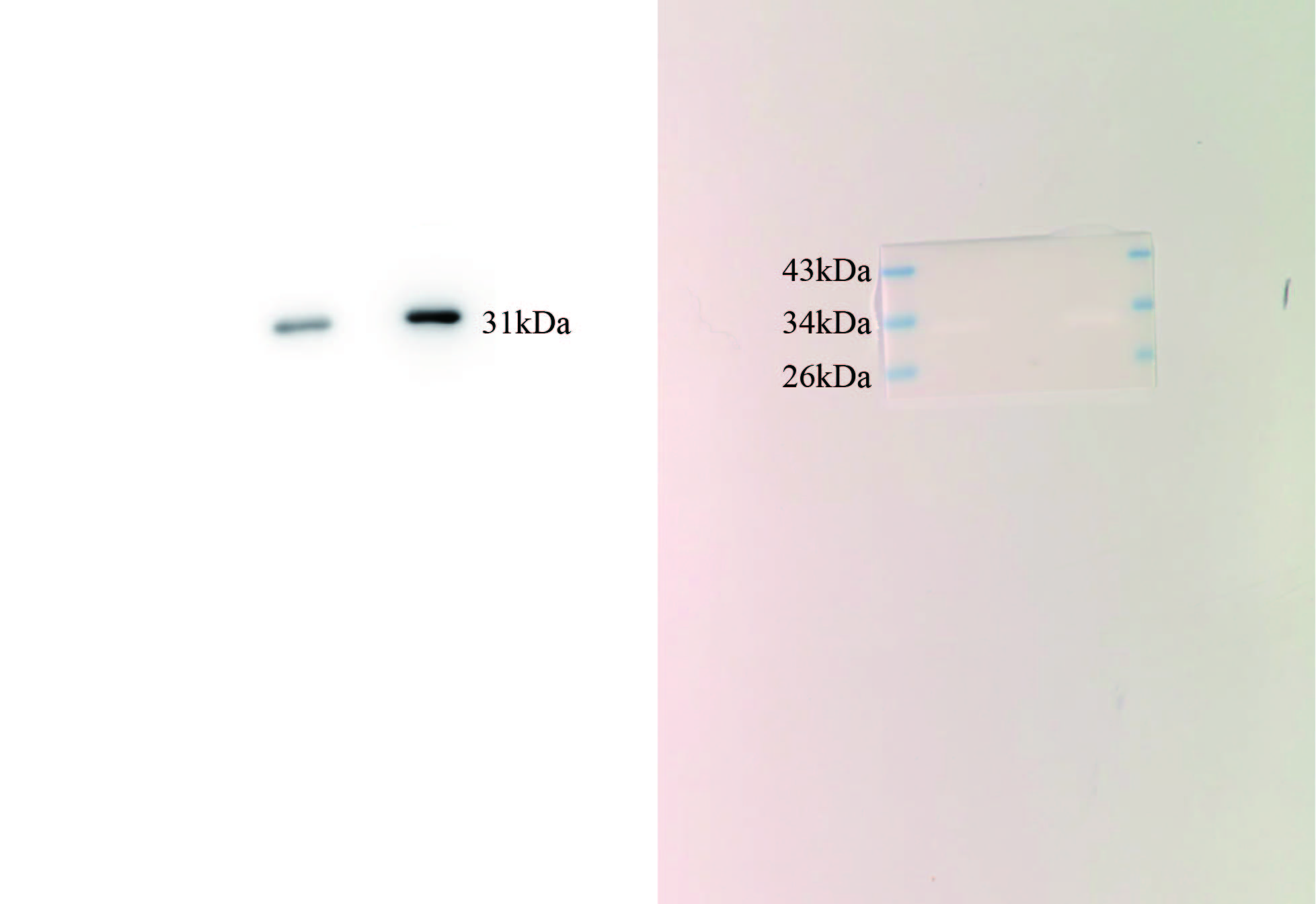

Supplement: Supplementary file 1 [file cancers-14-05434-s001.zip › Fig6-CO-IP-WB-SW-1990-CDCA8-1.jpg]

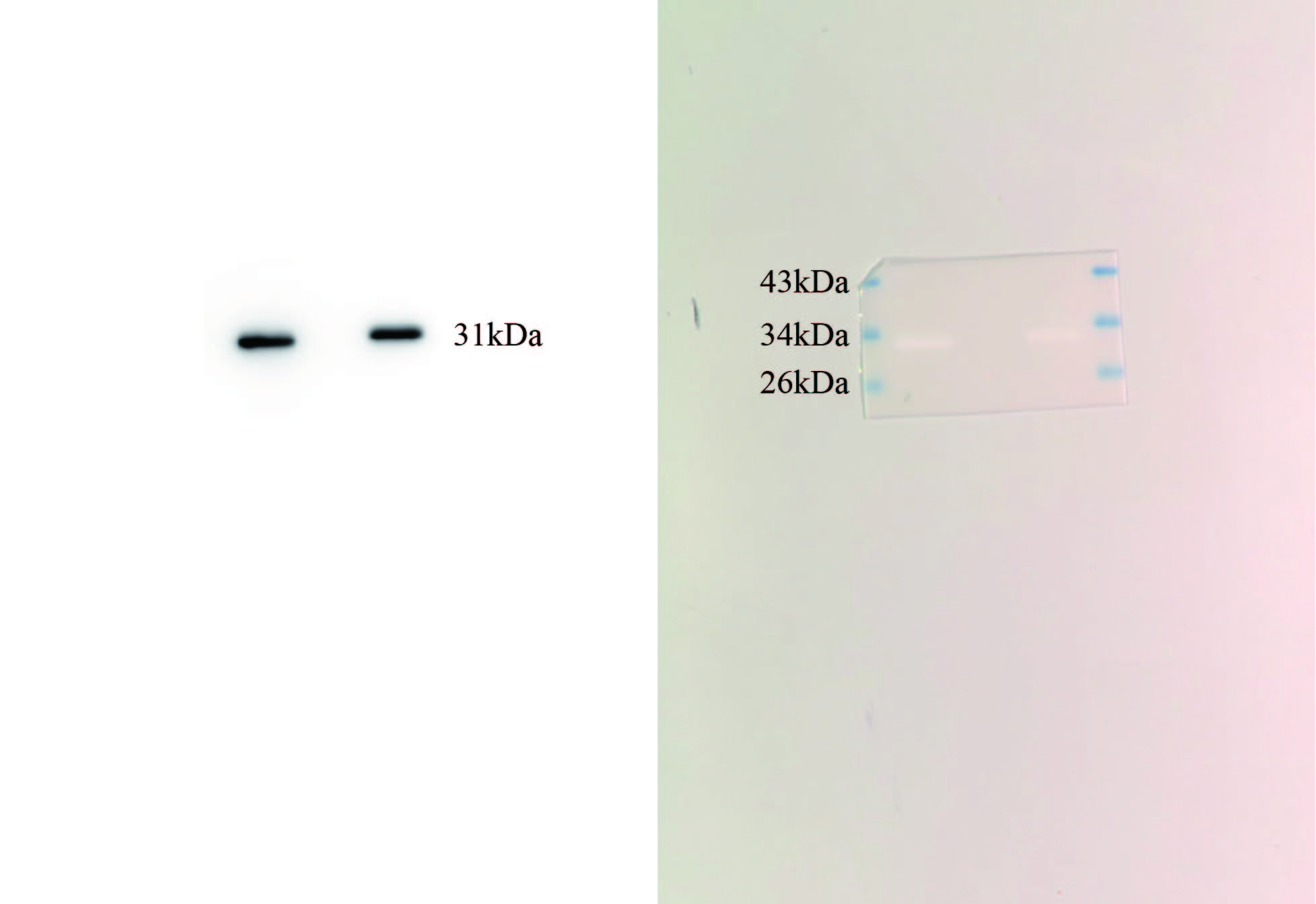

Supplement: Supplementary file 1 [file cancers-14-05434-s001.zip › Fig6-CO-IP-WB-SW-1990-CDCA8-2.jpg]

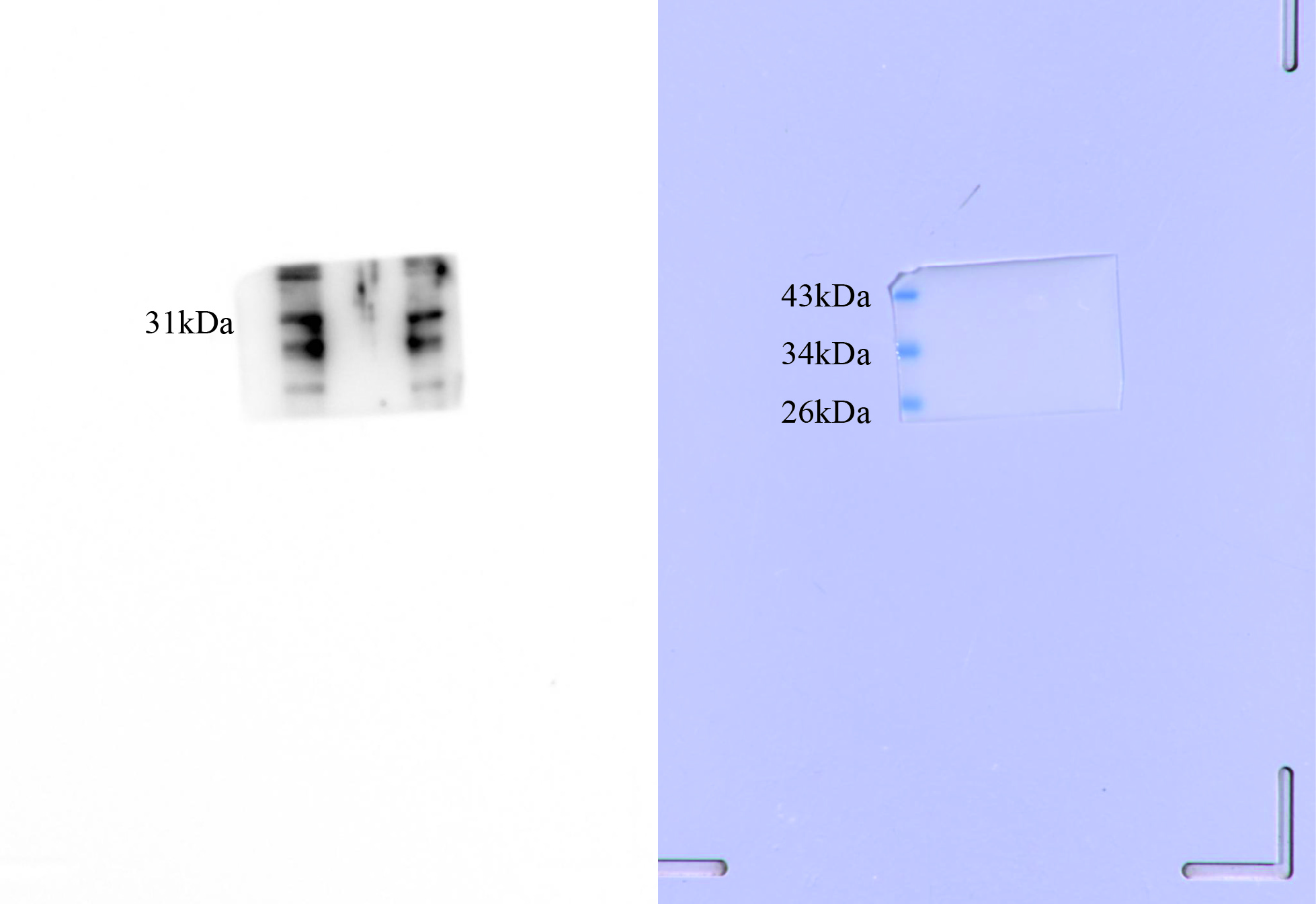

Supplement: Supplementary file 1 [file cancers-14-05434-s001.zip › Fig6-CO-IP-WB-SW-1990-CDCA8-3.jpg]

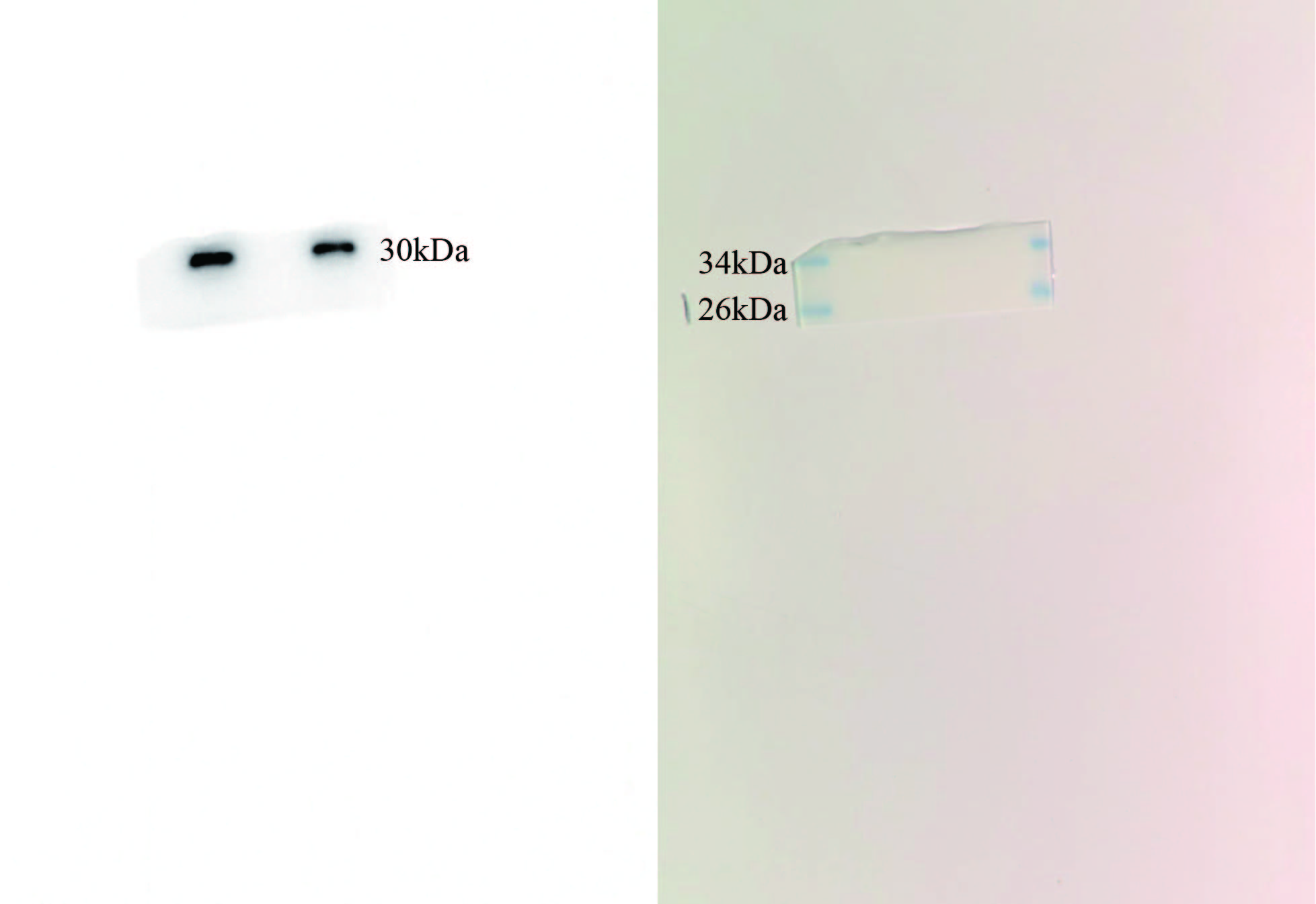

Supplement: Supplementary file 1 [file cancers-14-05434-s001.zip › Fig6-CO-IP-WB-SW-1990-SNAI2-1.jpg]

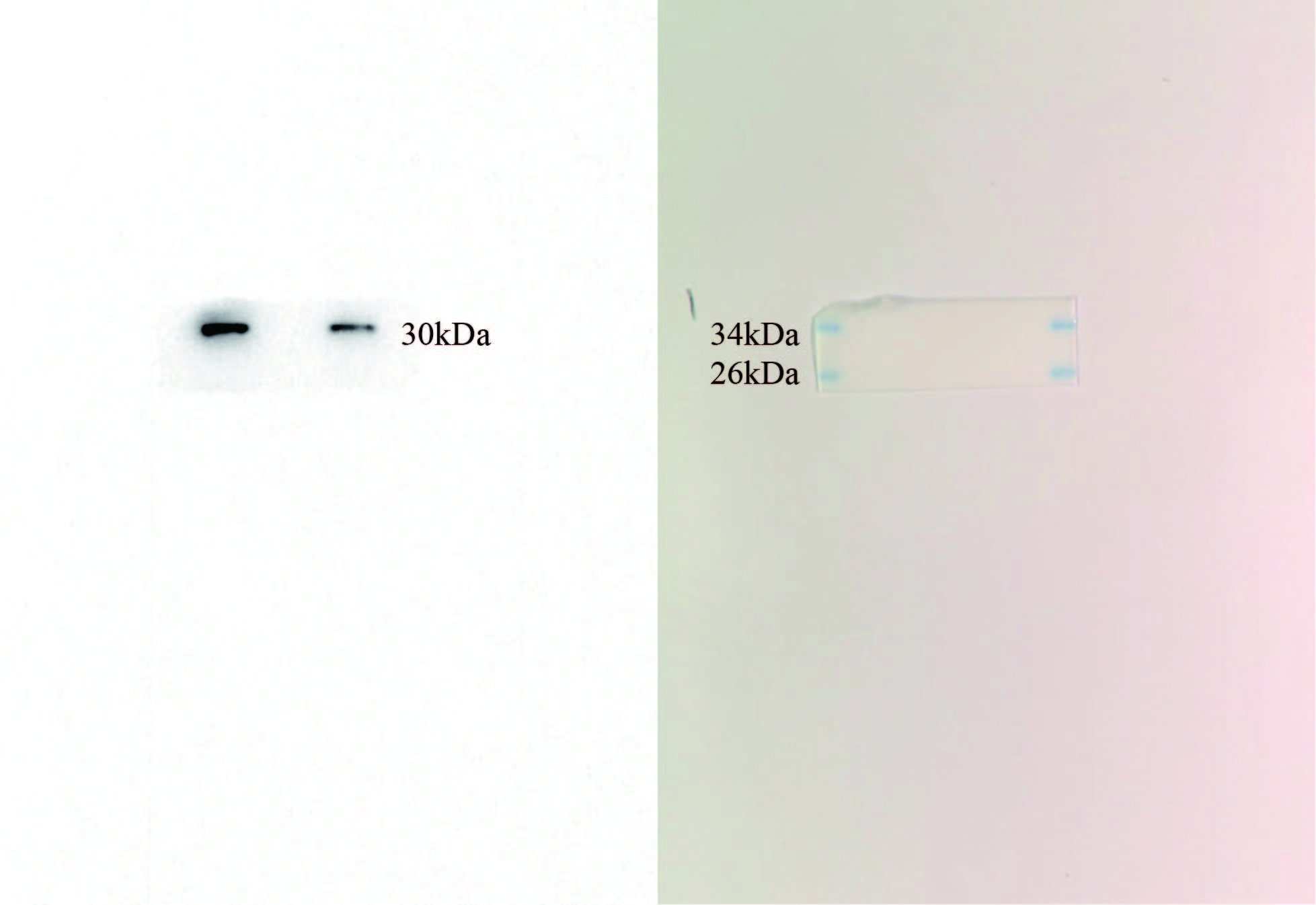

Supplement: Supplementary file 1 [file cancers-14-05434-s001.zip › Fig6-CO-IP-WB-SW-1990-SNAI2-2.jpg]

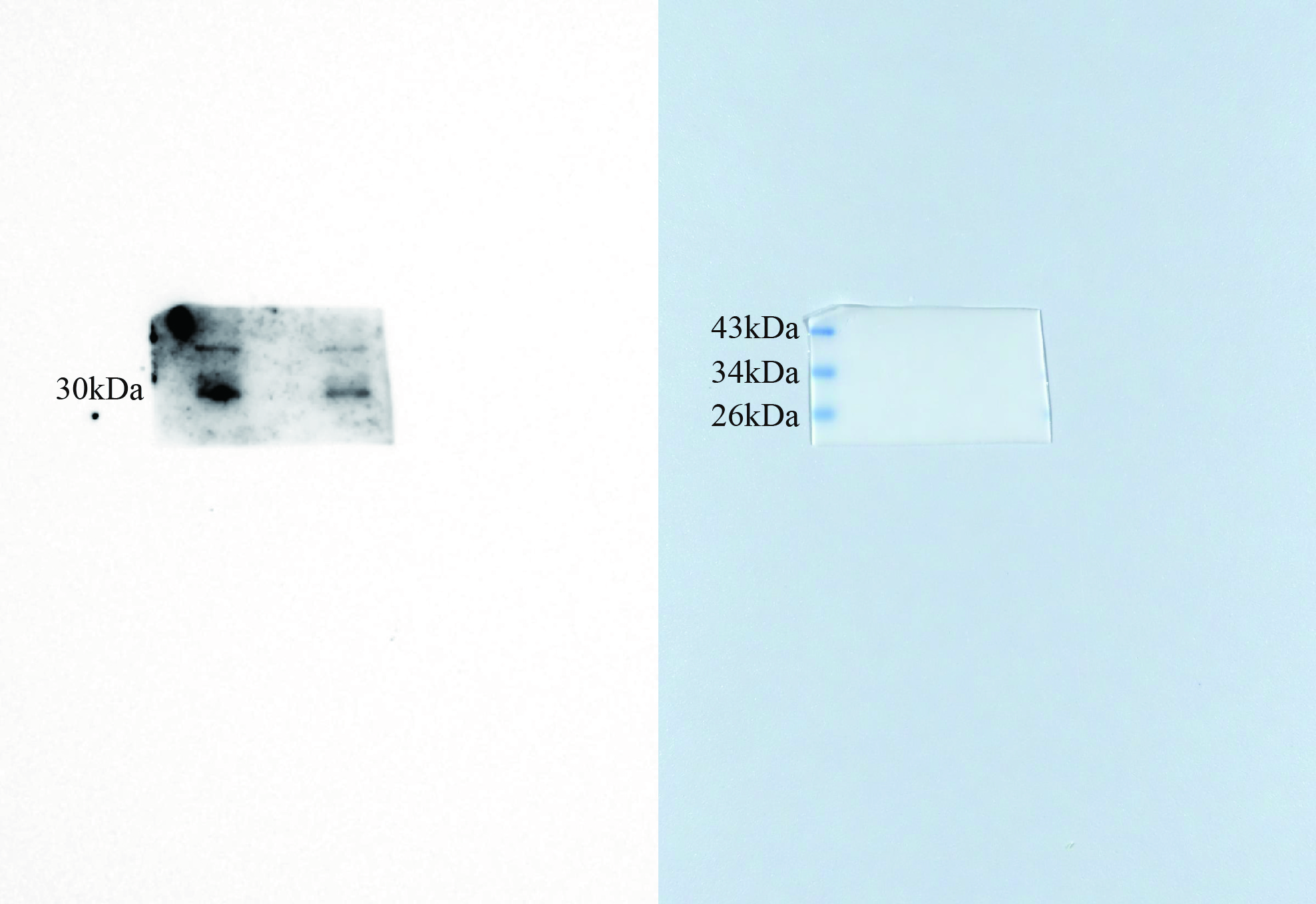

Supplement: Supplementary file 1 [file cancers-14-05434-s001.zip › Fig6-CO-IP-WB-SW-1990-SNAI2-3.jpg]

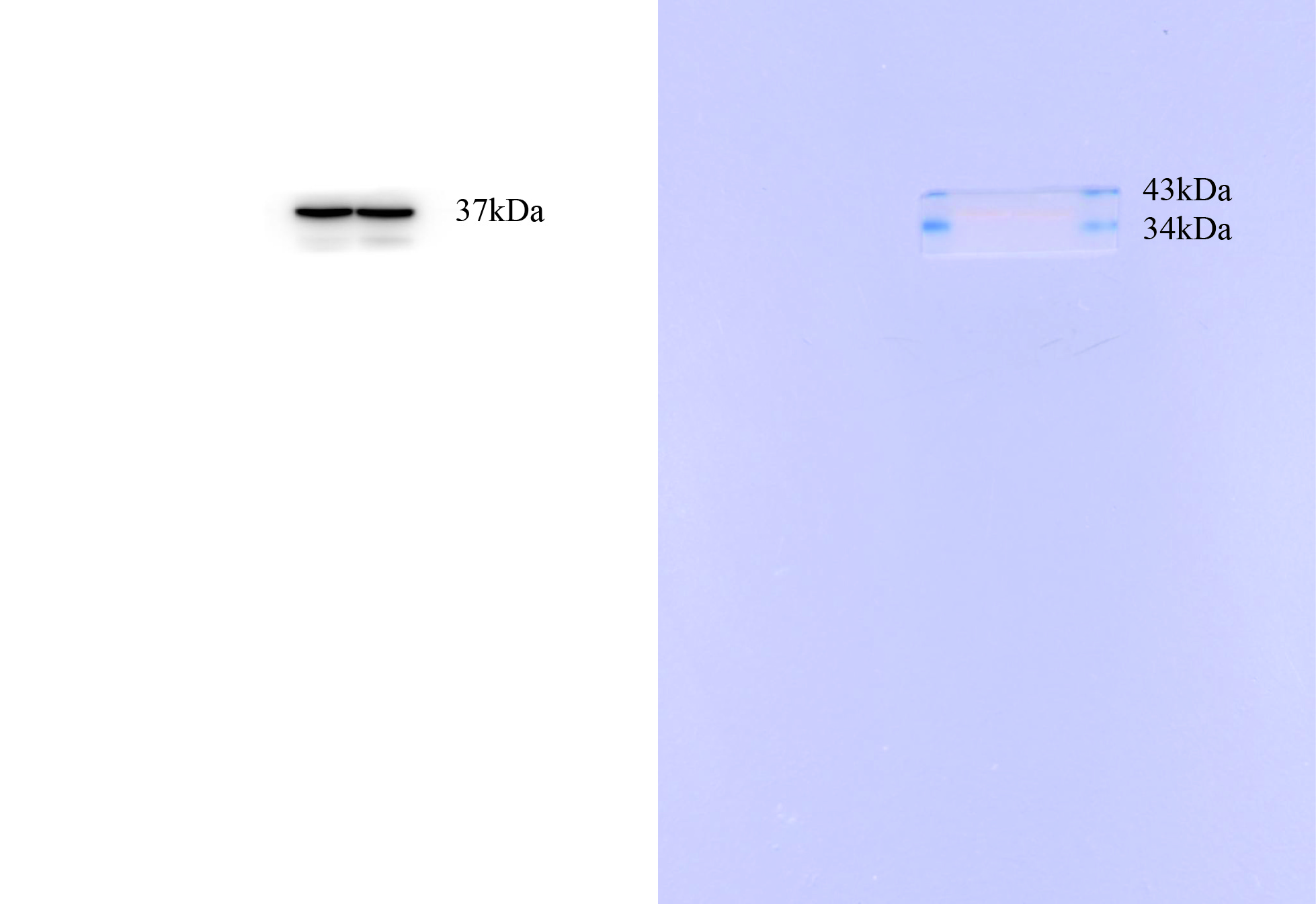

Supplement: Supplementary file 1 [file cancers-14-05434-s001.zip › FigS1-WB-PANC-1-shCDCA8 GAPDH-1.jpg]

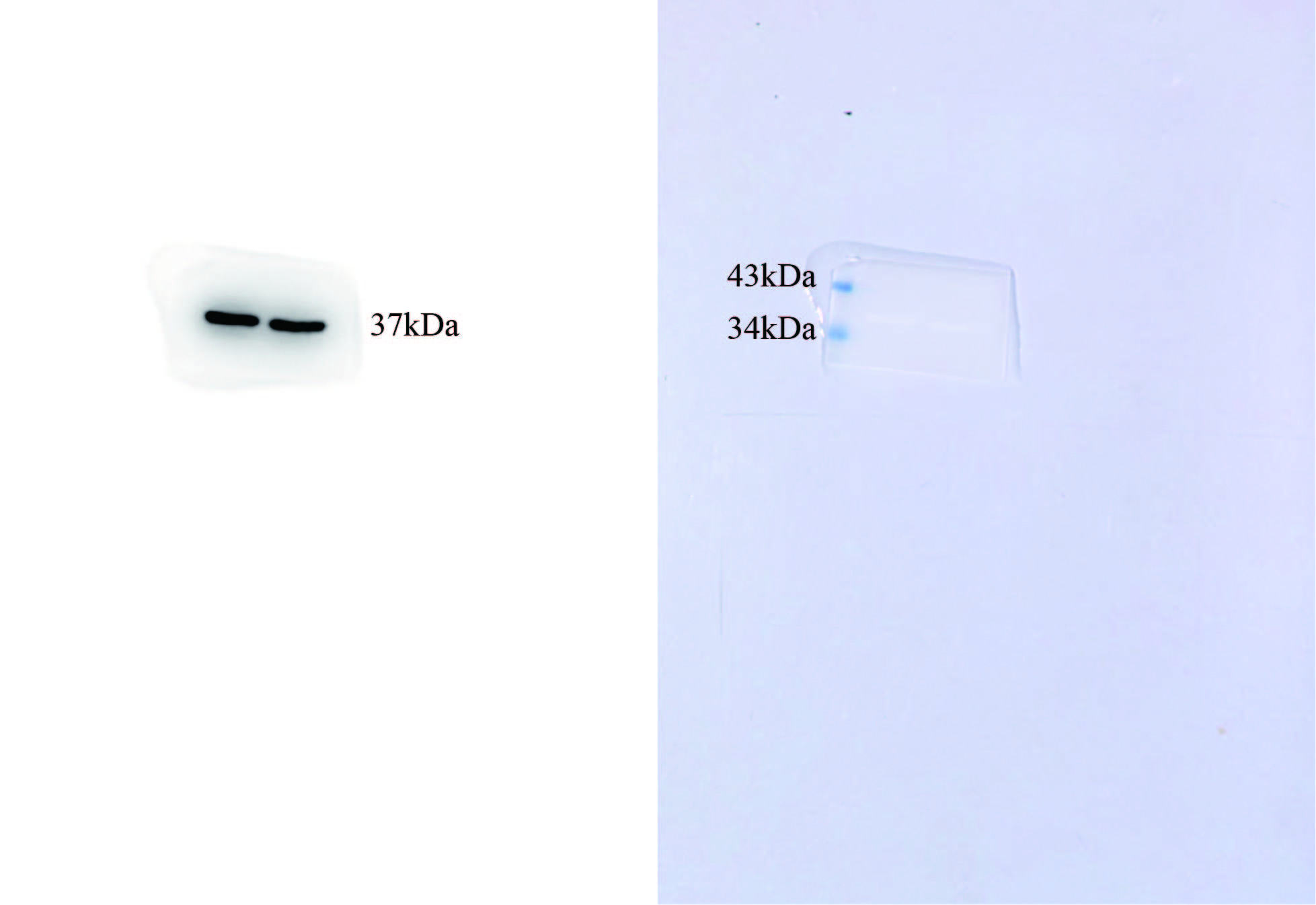

Supplement: Supplementary file 1 [file cancers-14-05434-s001.zip › FigS1-WB-PANC-1-shCDCA8 GAPDH-2.jpg]

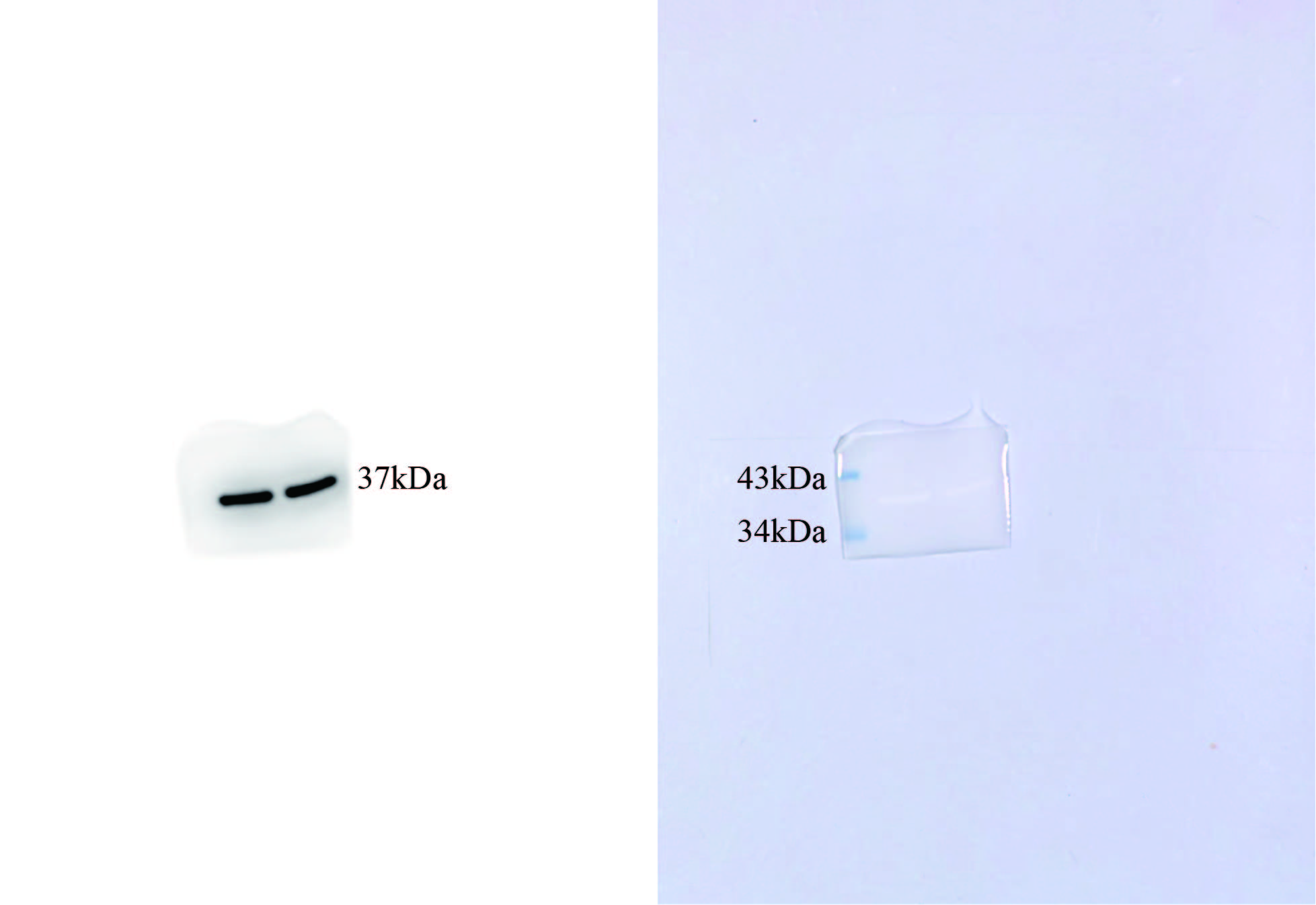

Supplement: Supplementary file 1 [file cancers-14-05434-s001.zip › FigS1-WB-PANC-1-shCDCA8 GAPDH-3.jpg]

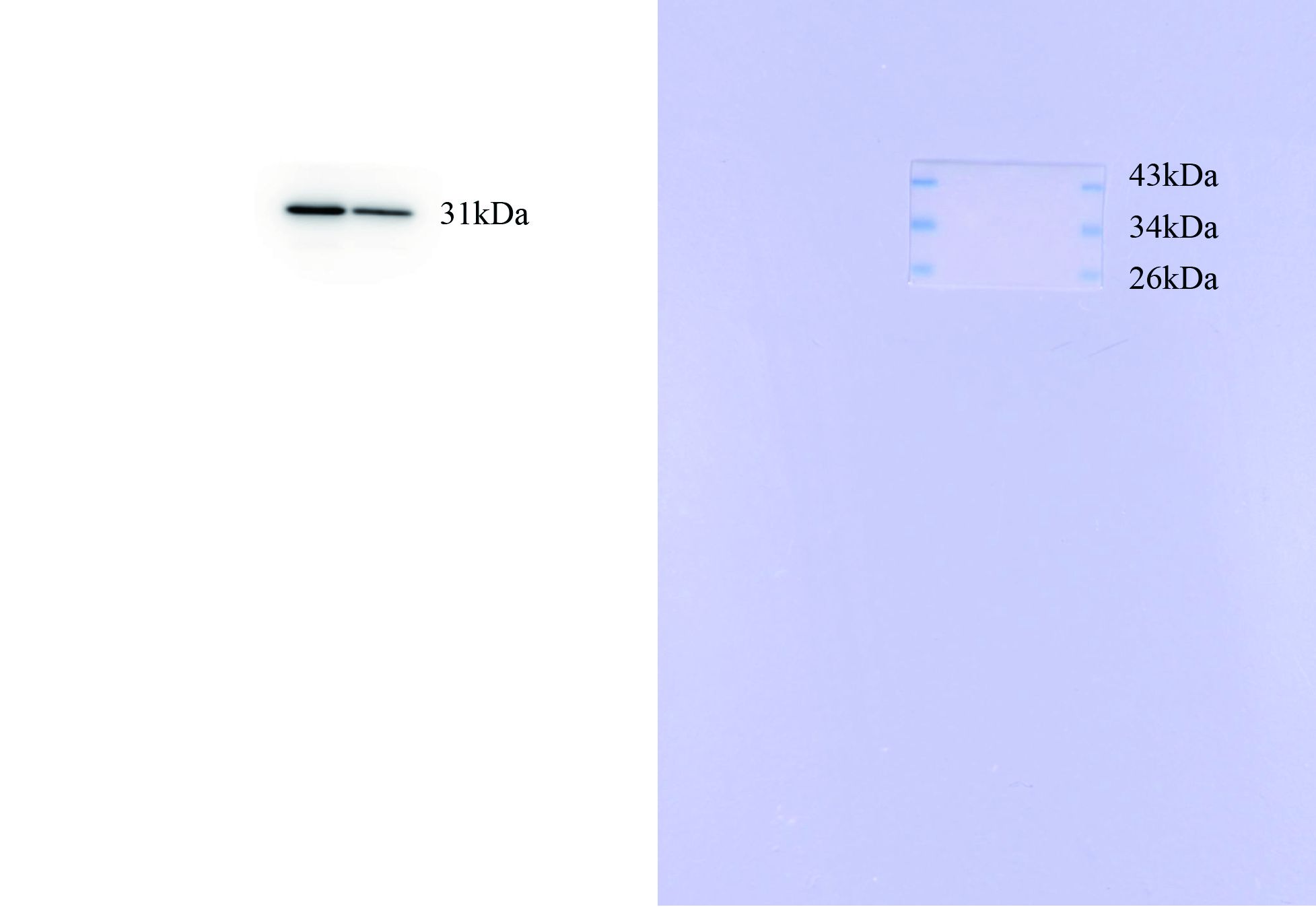

Supplement: Supplementary file 1 [file cancers-14-05434-s001.zip › FigS1-WB-PANC-1-shCDCA8-1.jpg]

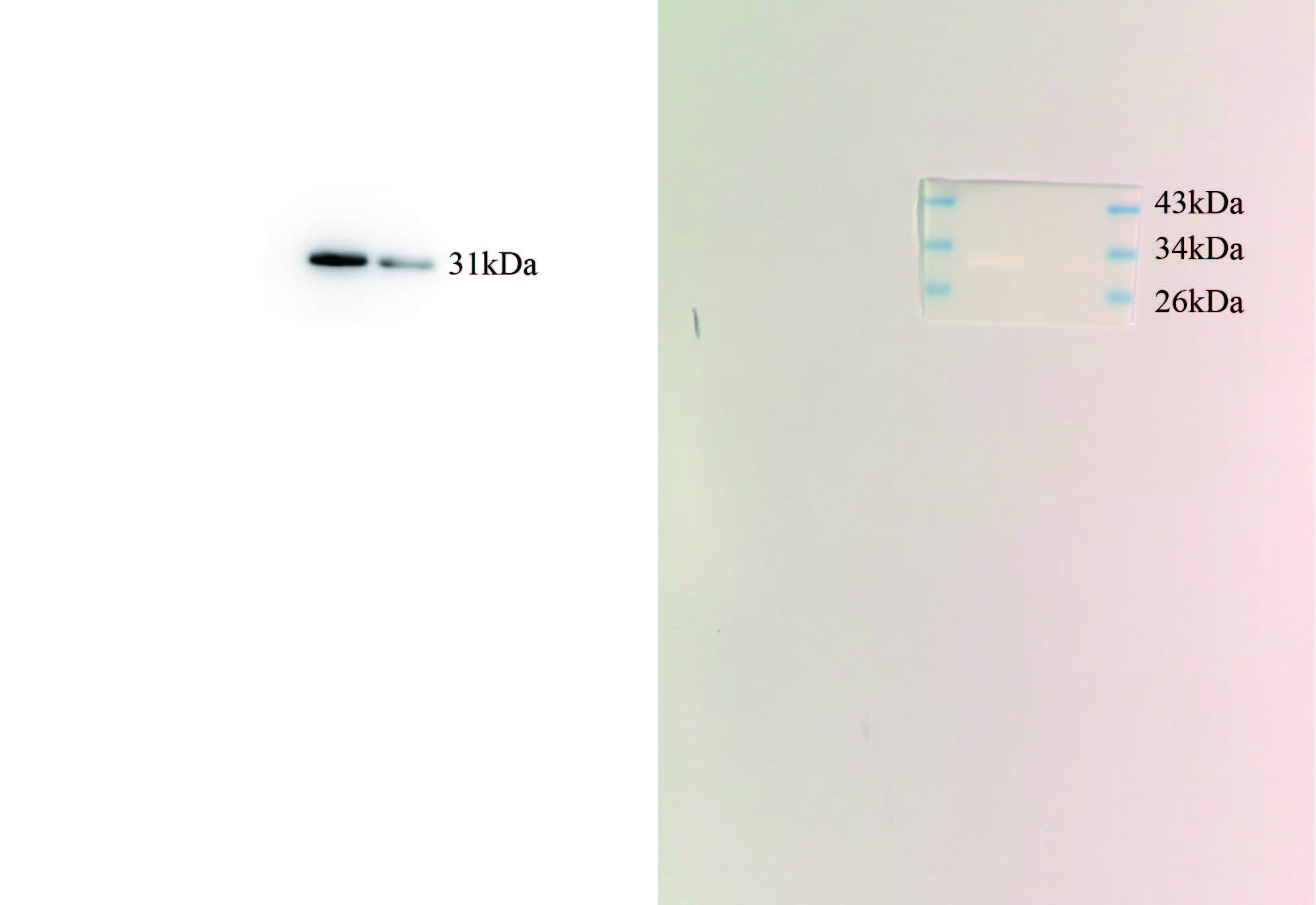

Supplement: Supplementary file 1 [file cancers-14-05434-s001.zip › FigS1-WB-PANC-1-shCDCA8-2.jpg]

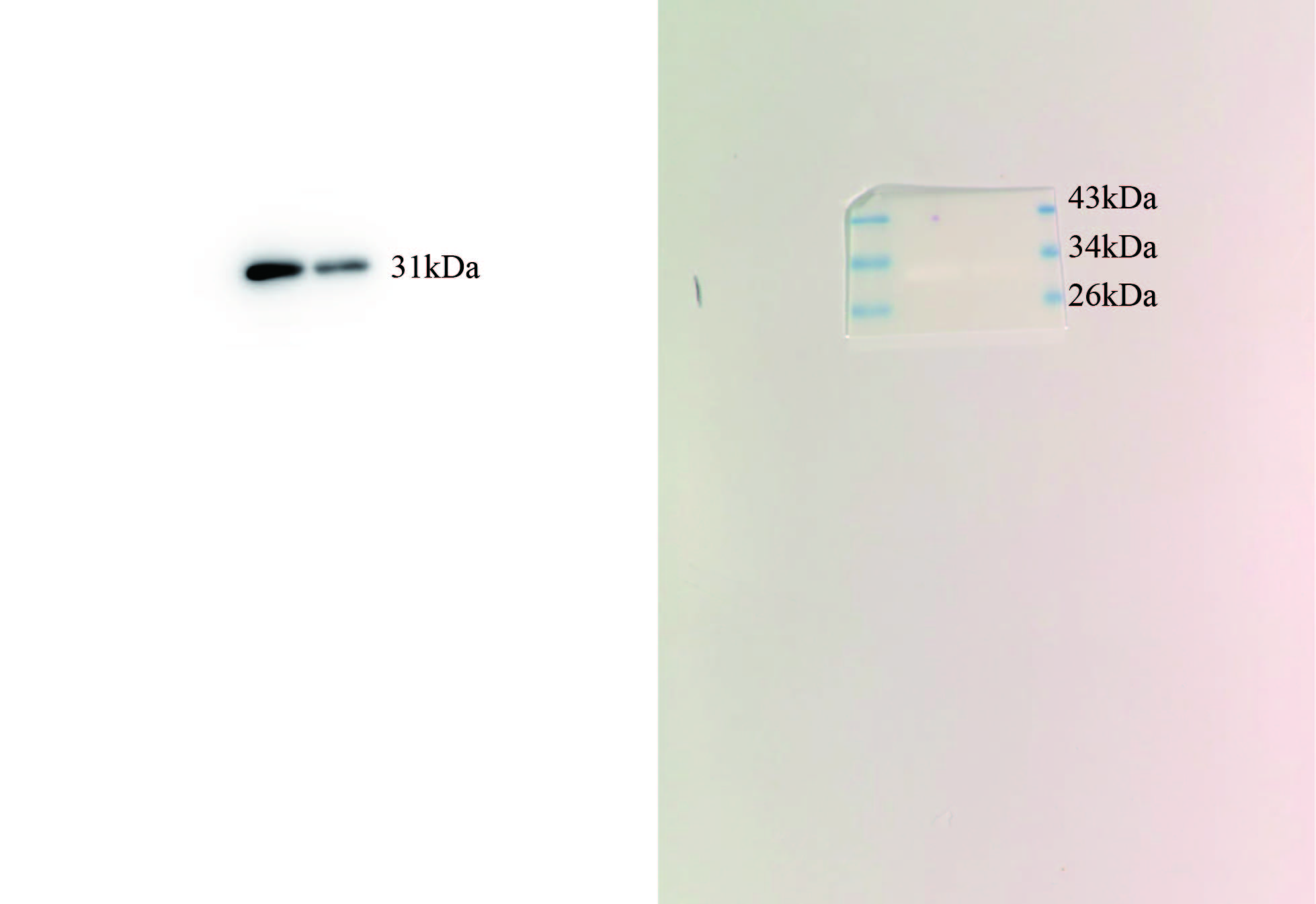

Supplement: Supplementary file 1 [file cancers-14-05434-s001.zip › FigS1-WB-PANC-1-shCDCA8-3.jpg]

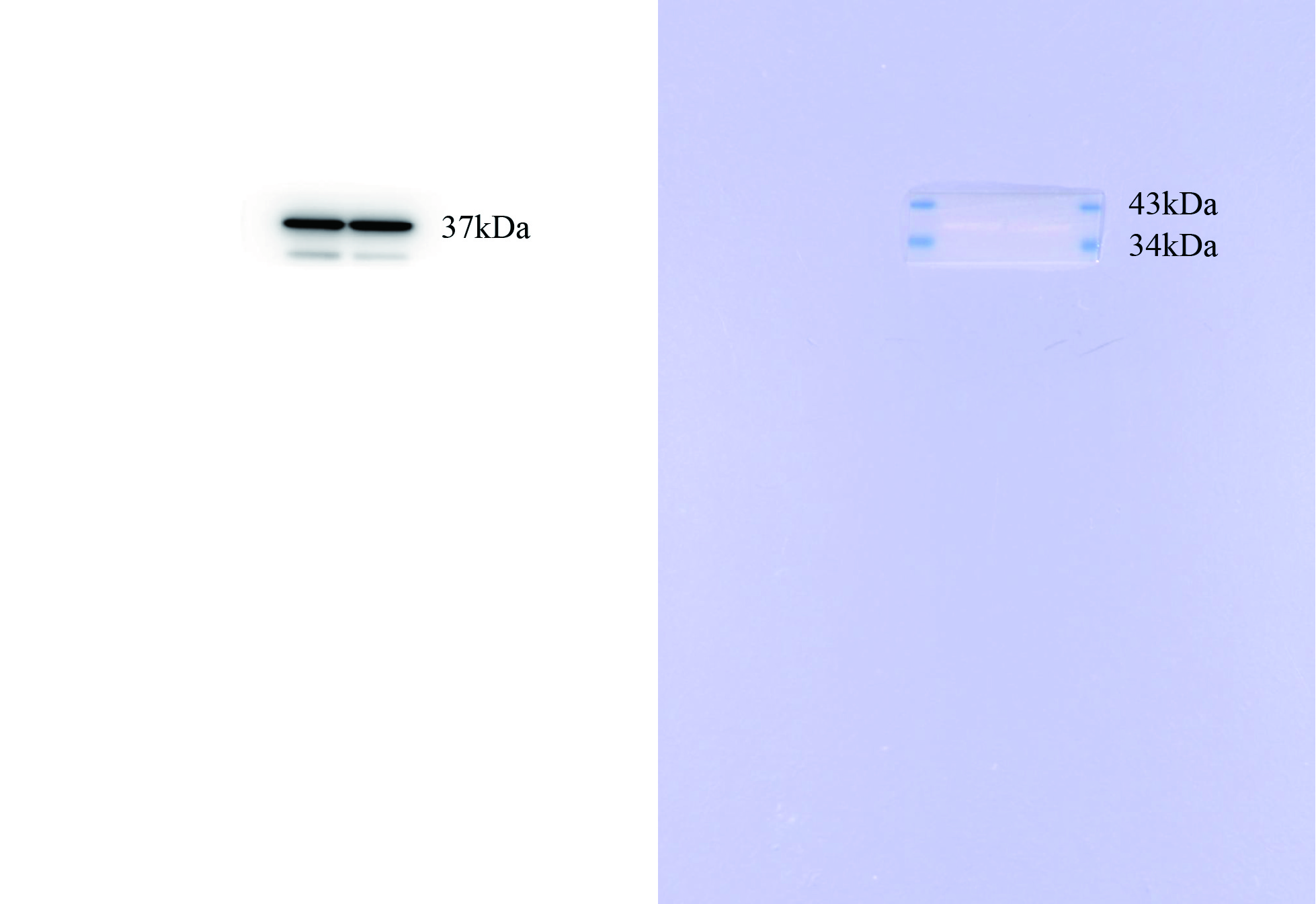

Supplement: Supplementary file 1 [file cancers-14-05434-s001.zip › FigS1-WB-SW1990-shCDCA8 GAPDH-1.jpg]

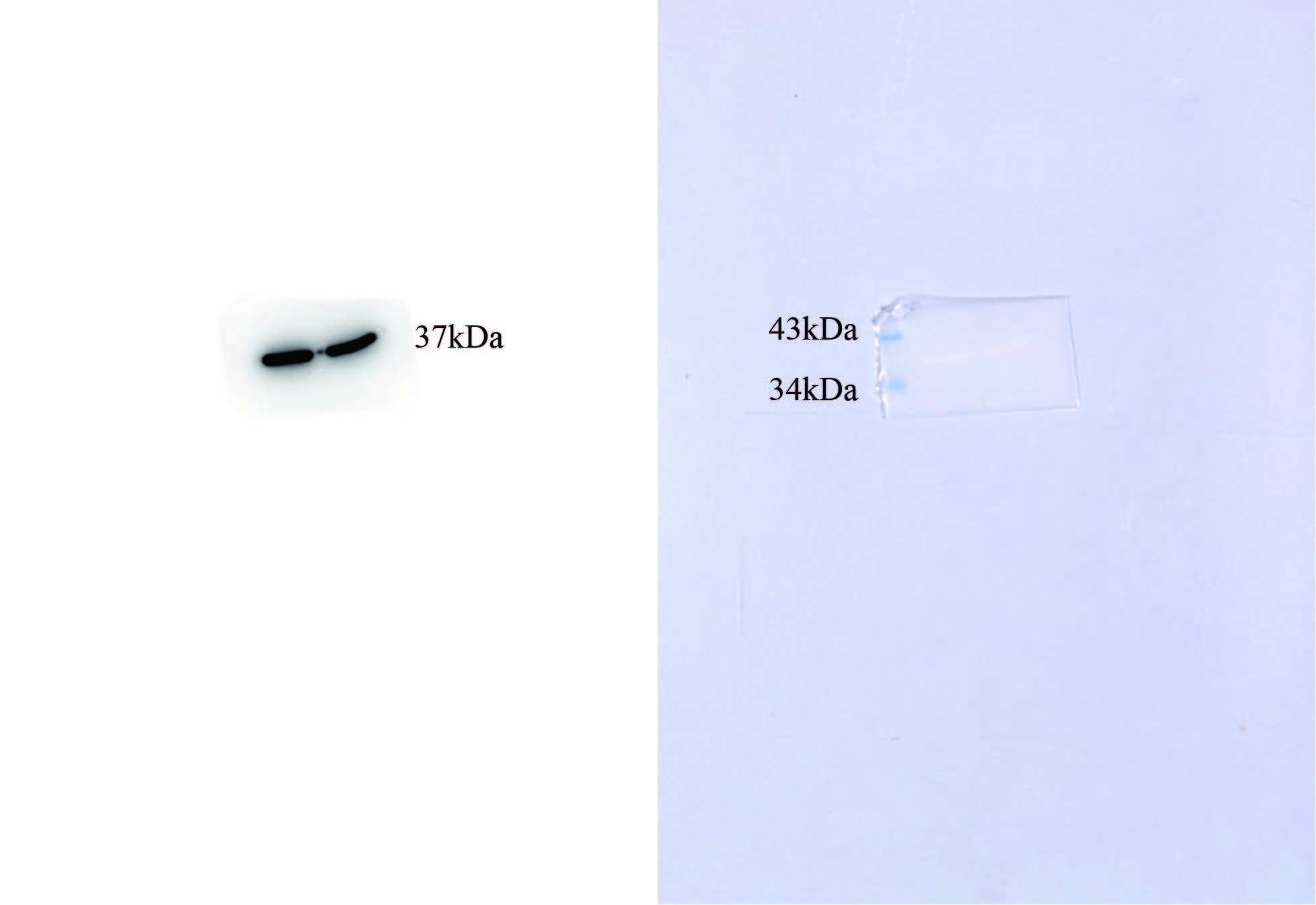

Supplement: Supplementary file 1 [file cancers-14-05434-s001.zip › FigS1-WB-SW1990-shCDCA8 GAPDH-2.jpg]

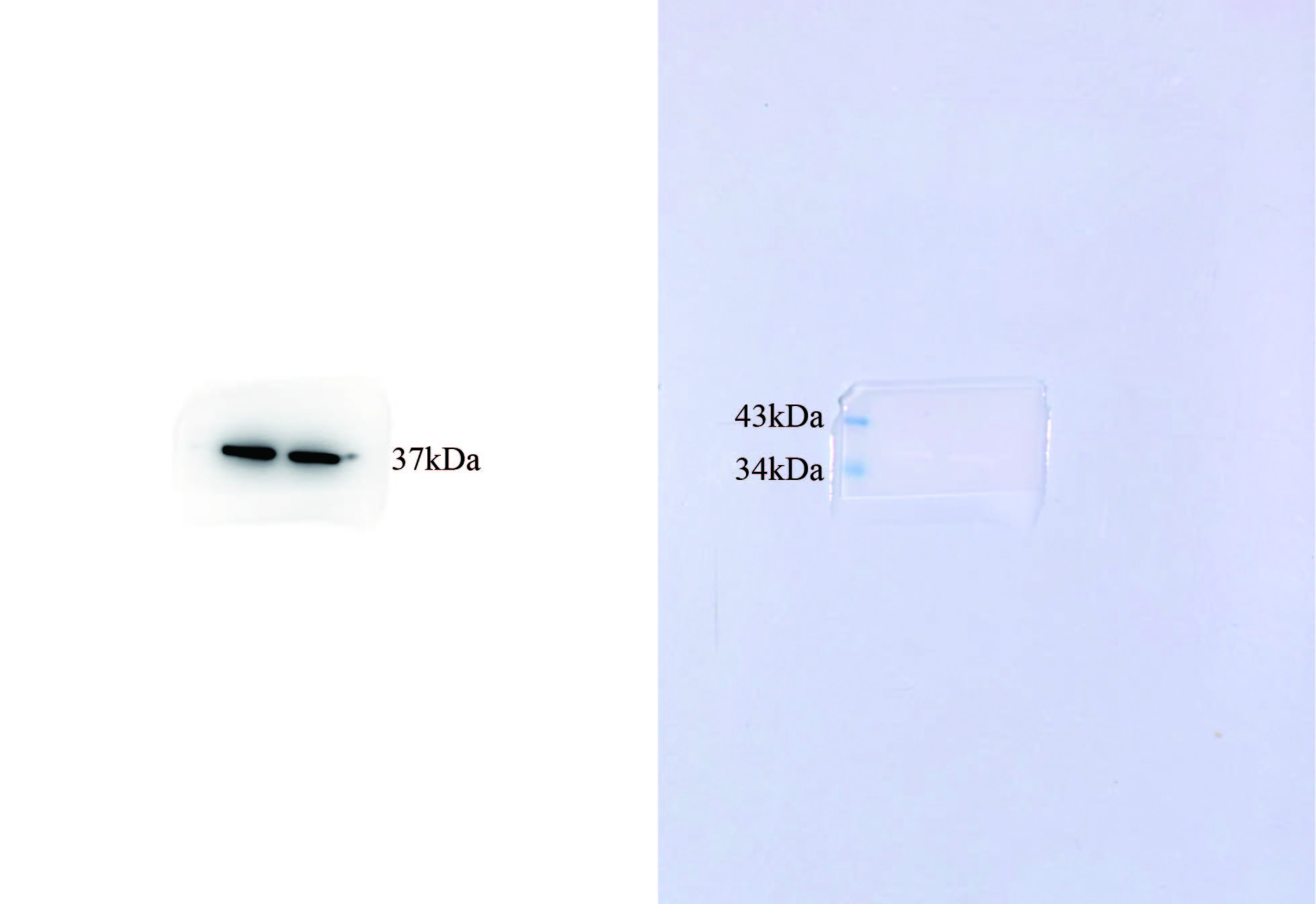

Supplement: Supplementary file 1 [file cancers-14-05434-s001.zip › FigS1-WB-SW1990-shCDCA8 GAPDH-3.jpg]

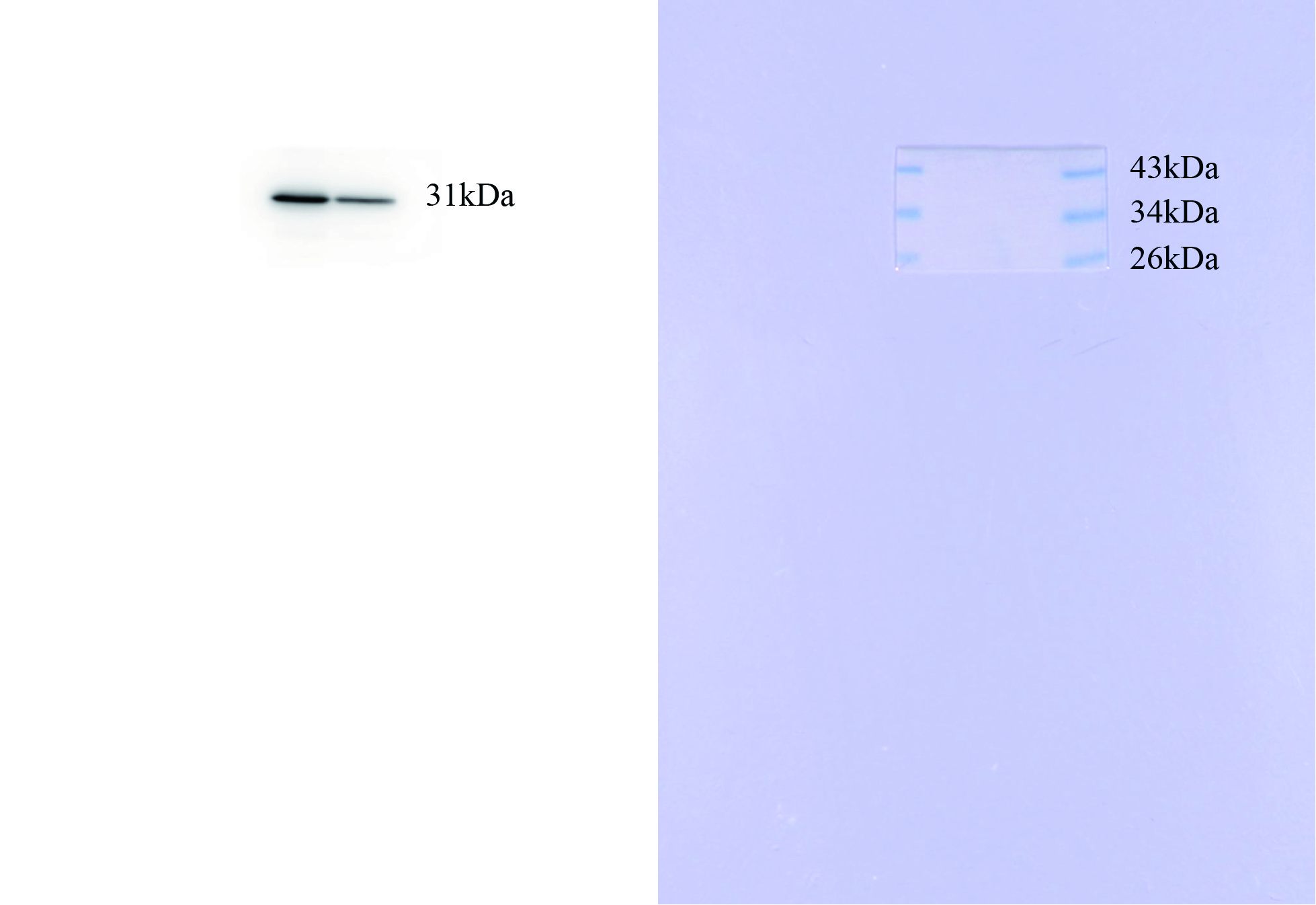

Supplement: Supplementary file 1 [file cancers-14-05434-s001.zip › FigS1-WB-SW1990-shCDCA8-1.jpg]

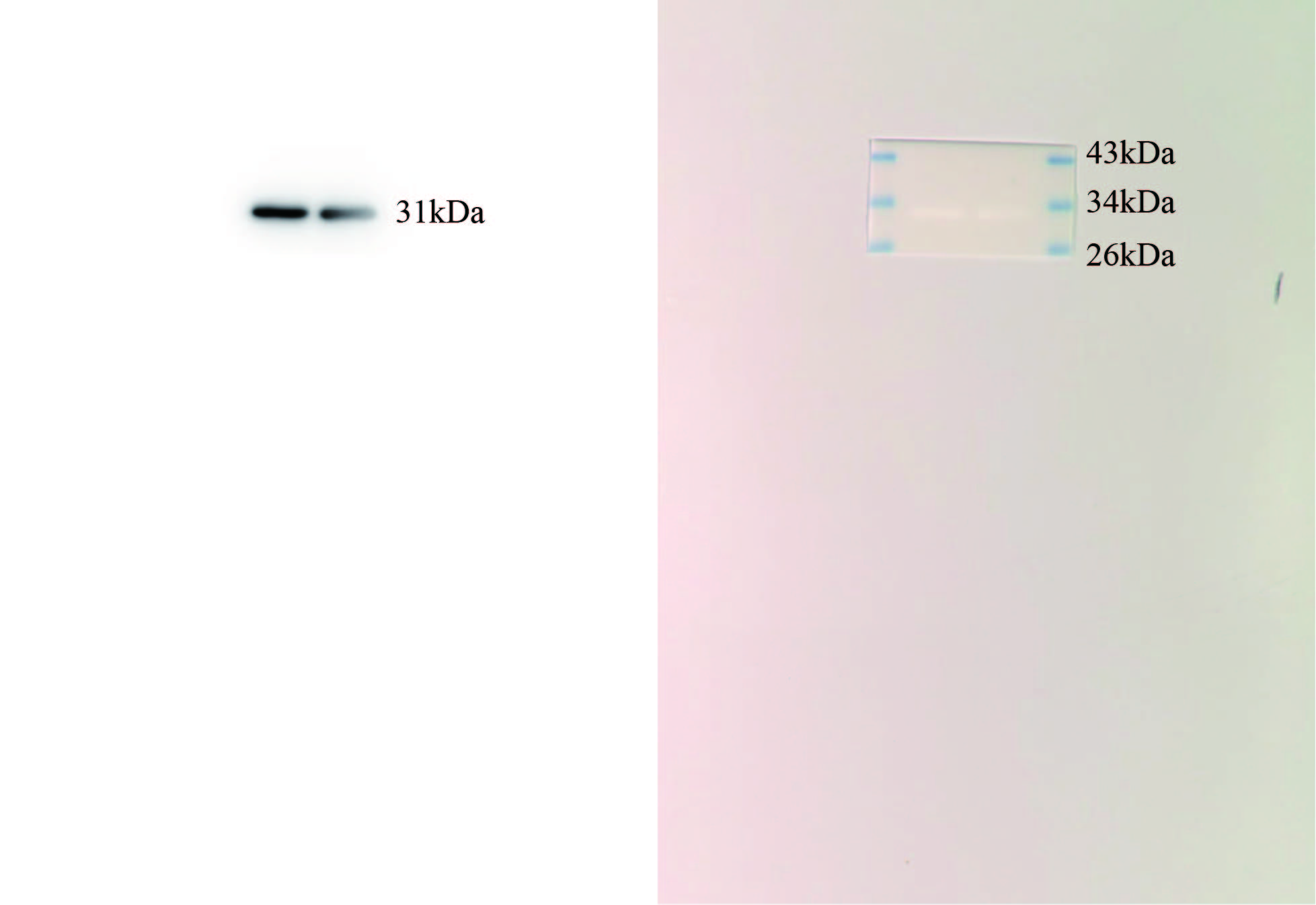

Supplement: Supplementary file 1 [file cancers-14-05434-s001.zip › FigS1-WB-SW1990-shCDCA8-2.jpg]

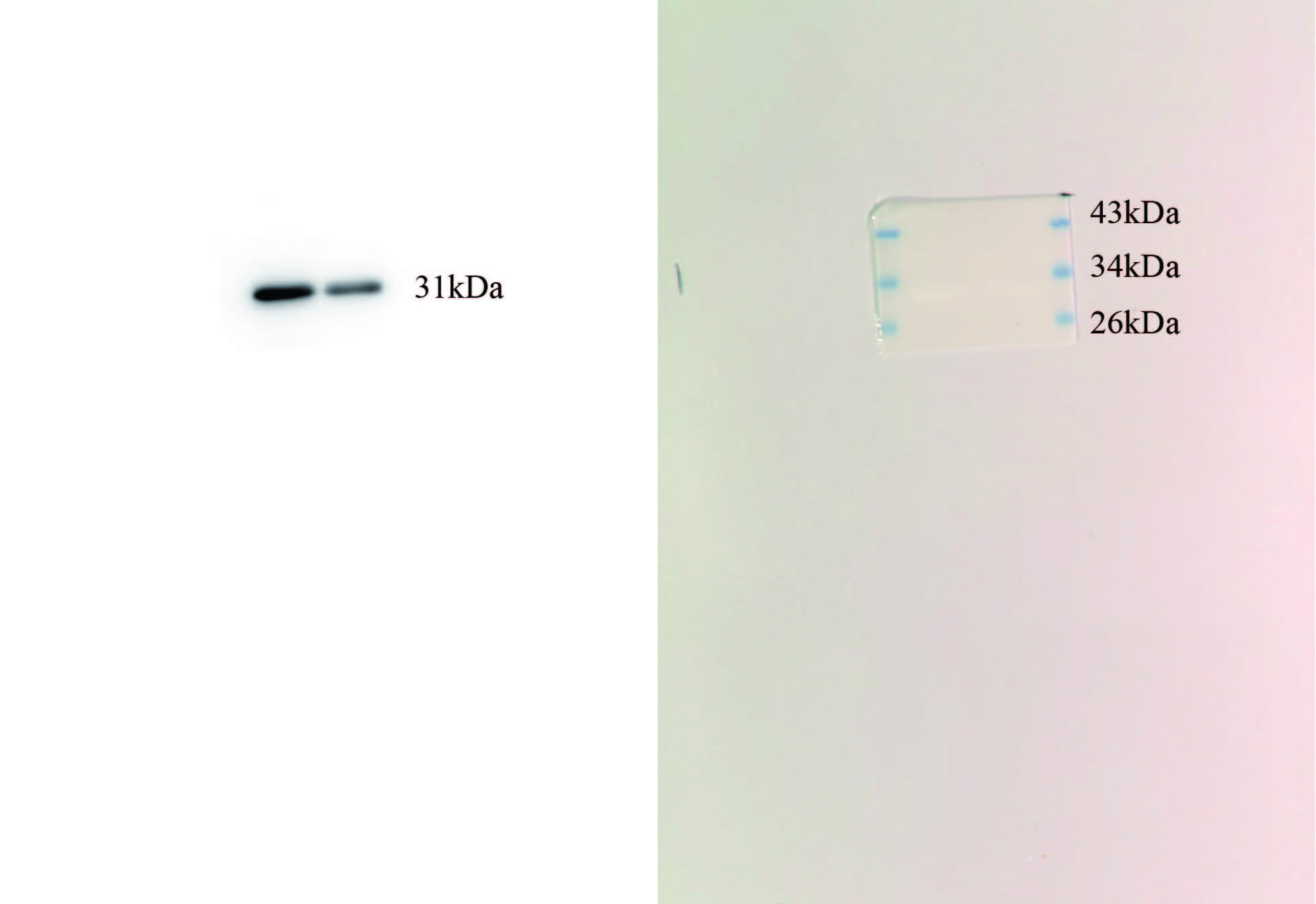

Supplement: Supplementary file 1 [file cancers-14-05434-s001.zip › FigS1-WB-SW1990-shCDCA8-3.jpg]

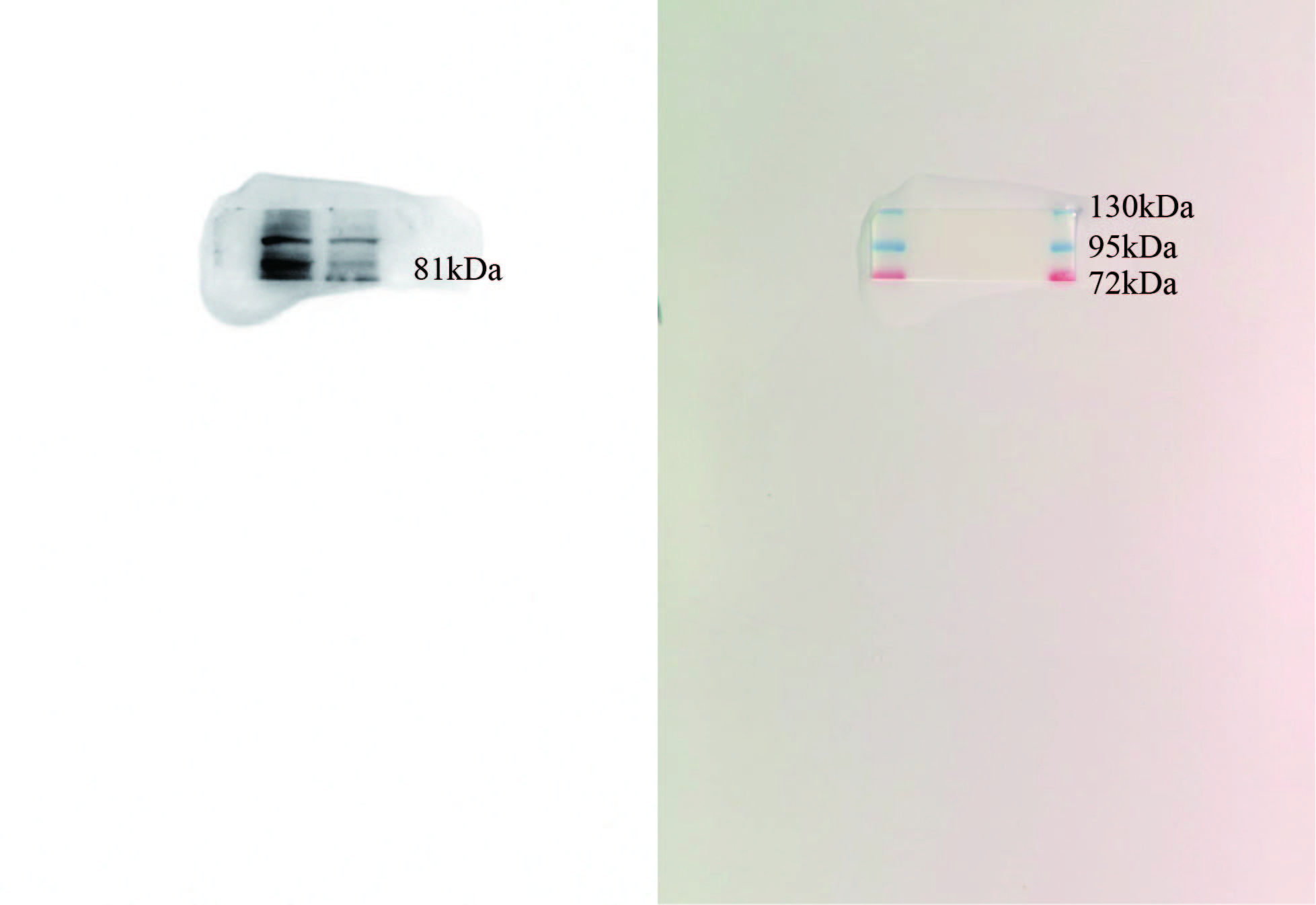

Supplement: Supplementary file 1 [file cancers-14-05434-s001.zip › FigS2-WB-PANC-1-CD44-1.jpg]

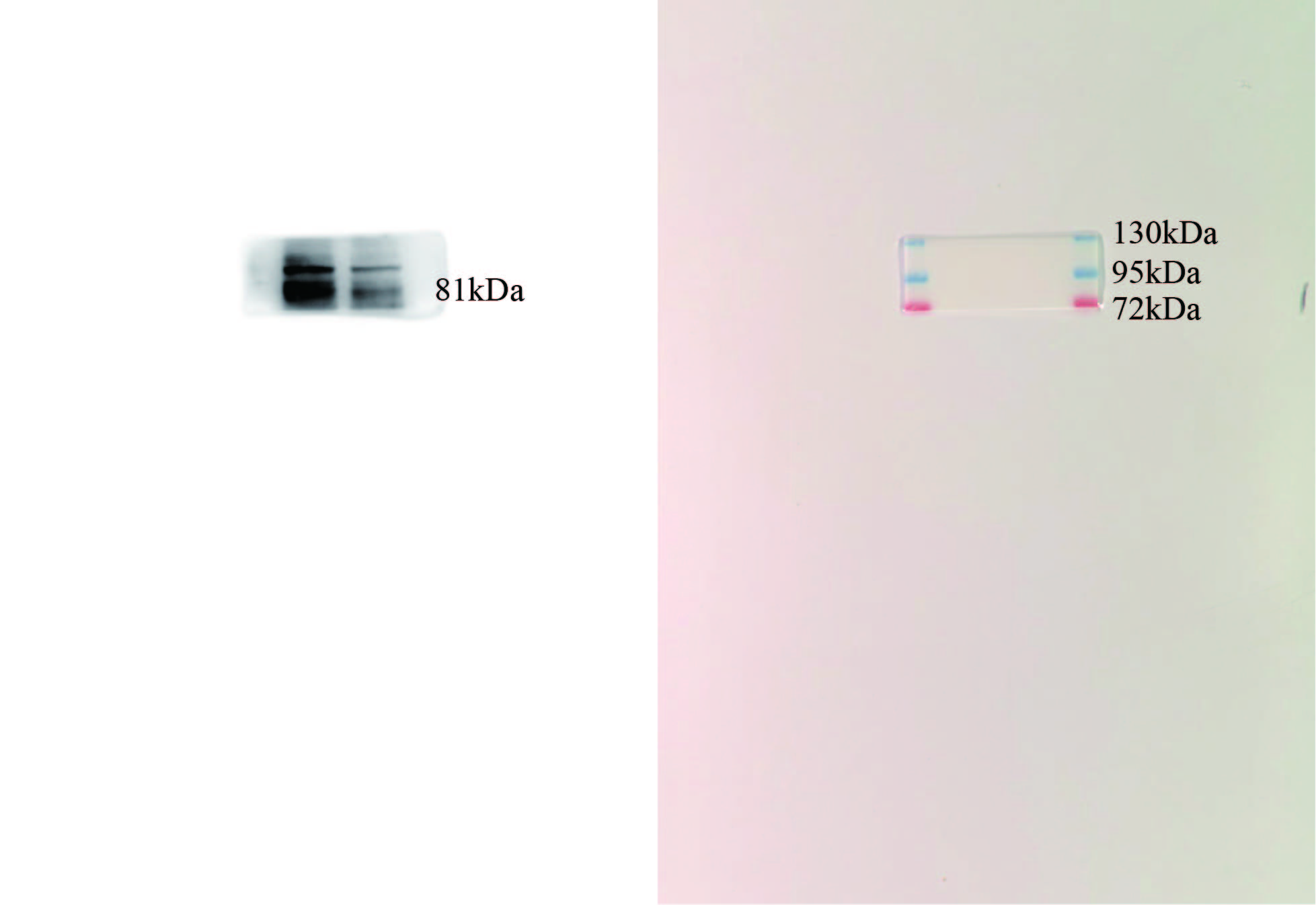

Supplement: Supplementary file 1 [file cancers-14-05434-s001.zip › FigS2-WB-PANC-1-CD44-2.jpg]

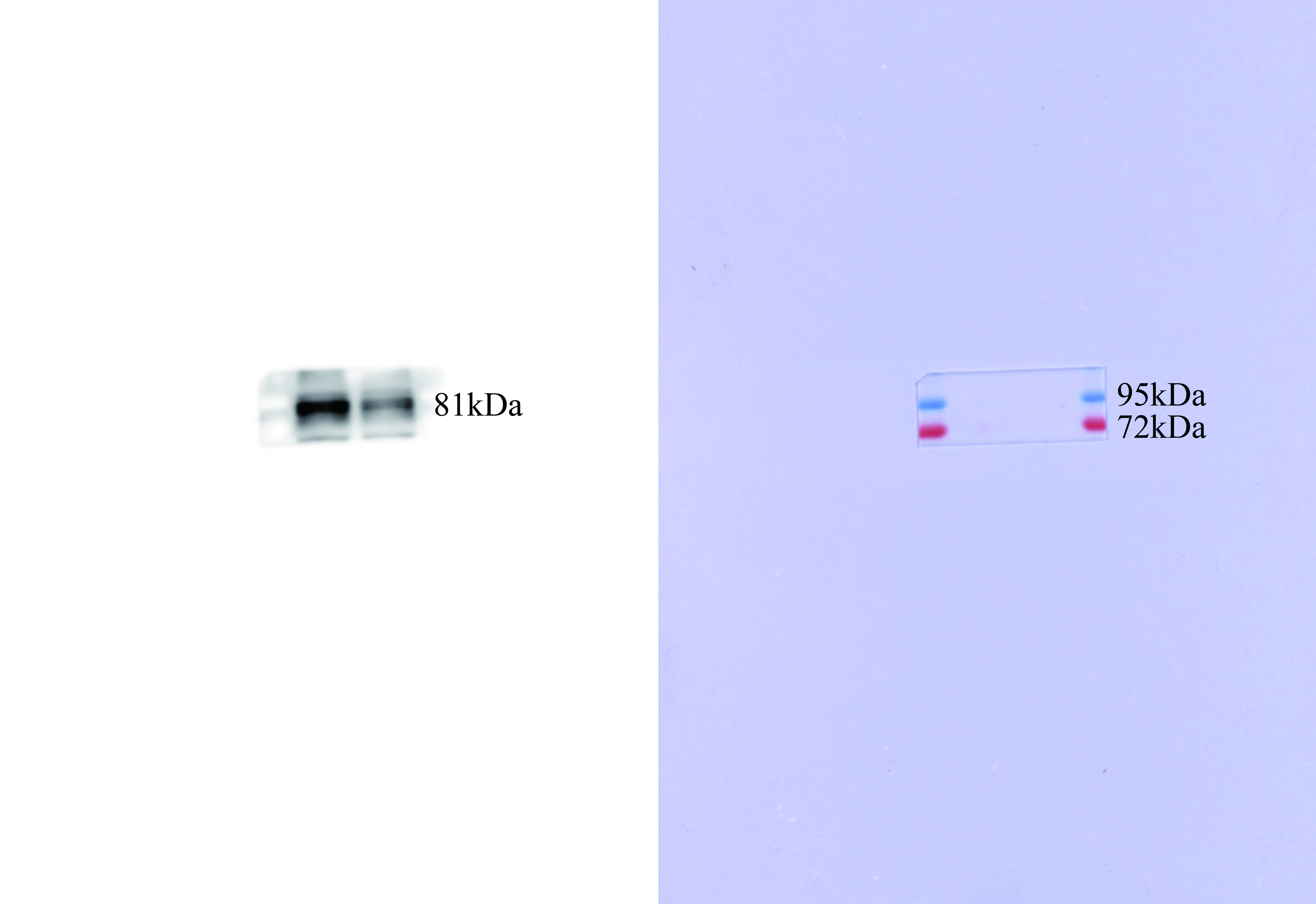

Supplement: Supplementary file 1 [file cancers-14-05434-s001.zip › FigS2-WB-PANC-1-CD44-3.jpg]

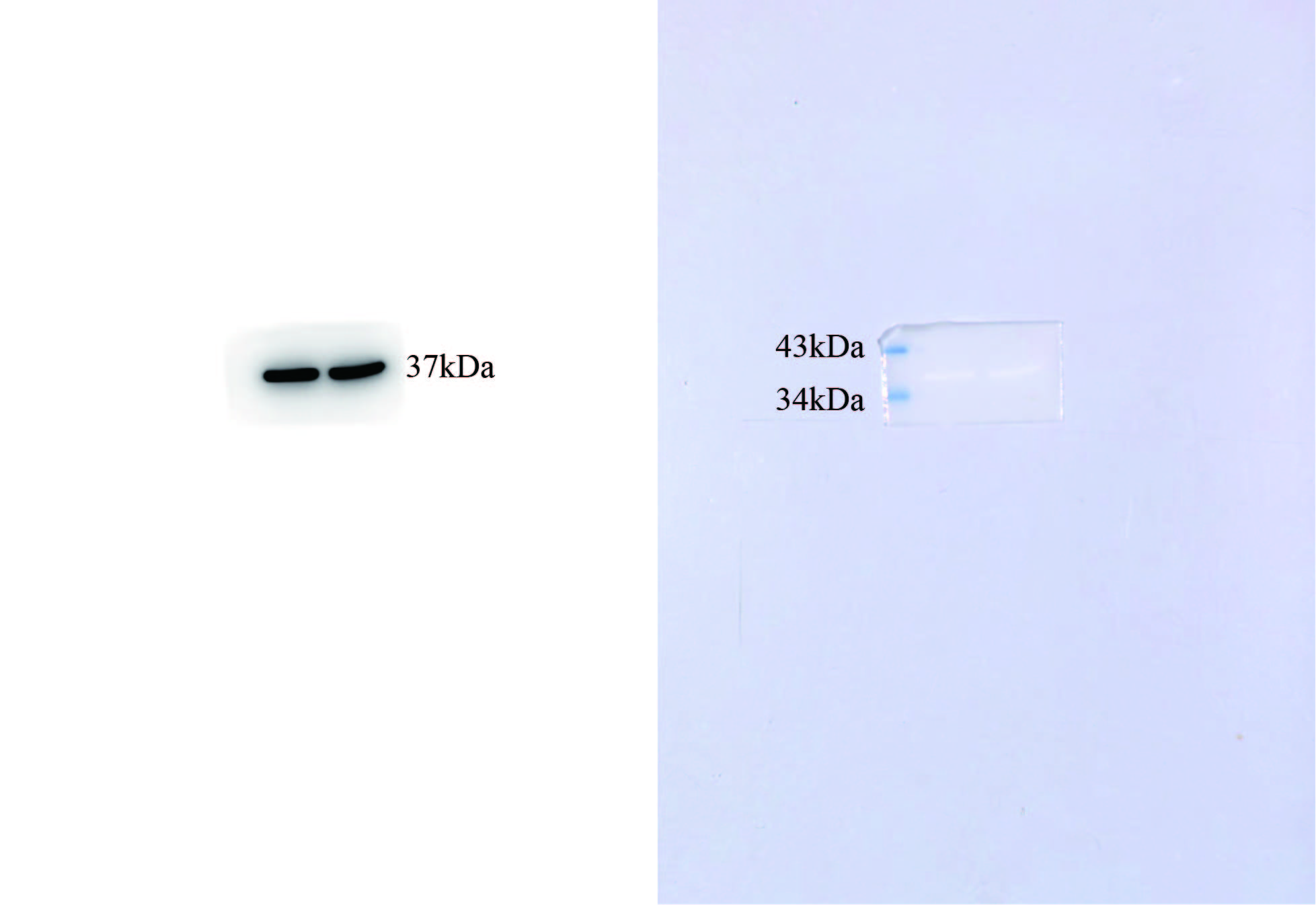

Supplement: Supplementary file 1 [file cancers-14-05434-s001.zip › FigS2-WB-PANC-1-GAPDH-1.jpg]

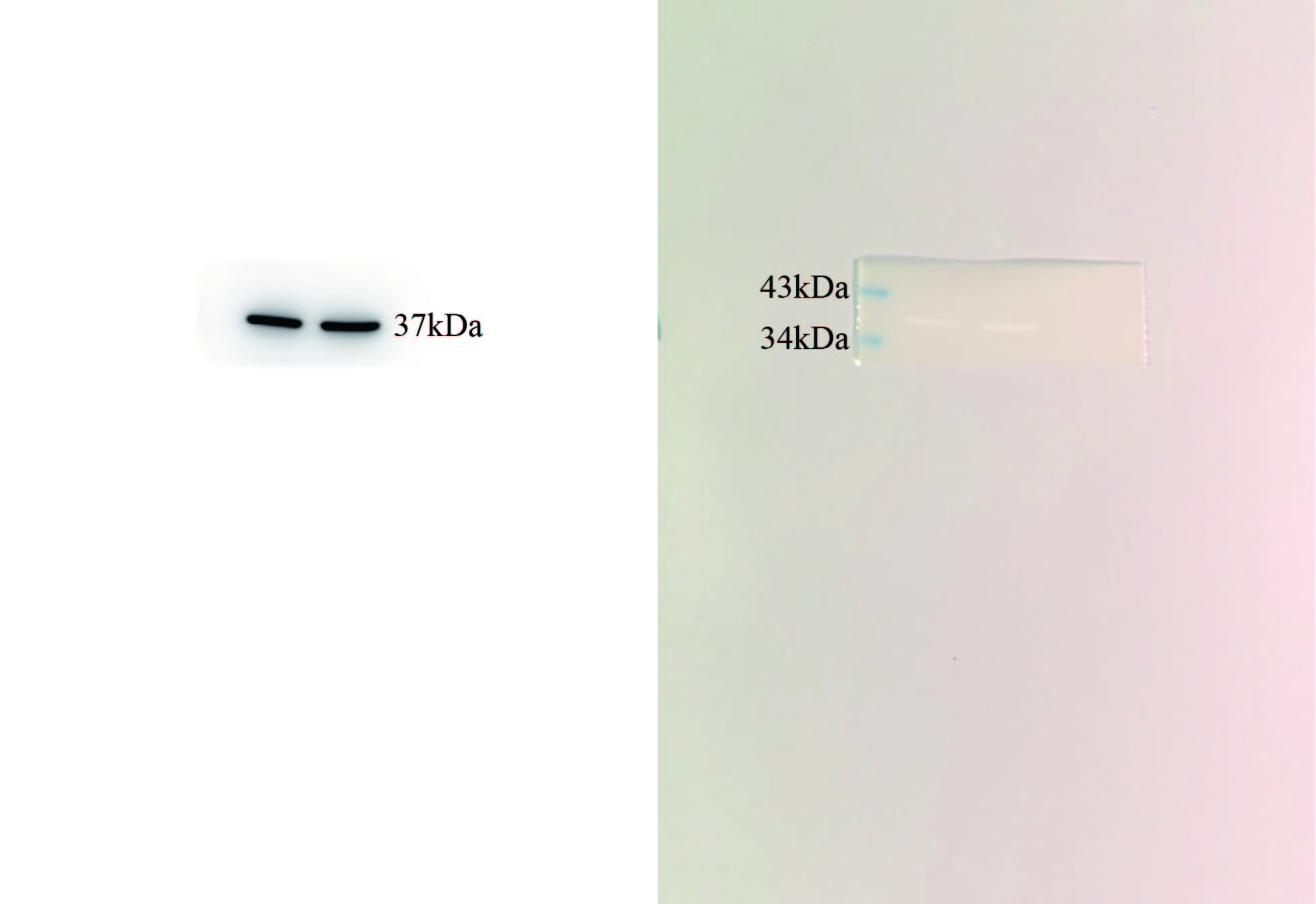

Supplement: Supplementary file 1 [file cancers-14-05434-s001.zip › FigS2-WB-PANC-1-GAPDH-2.jpg]

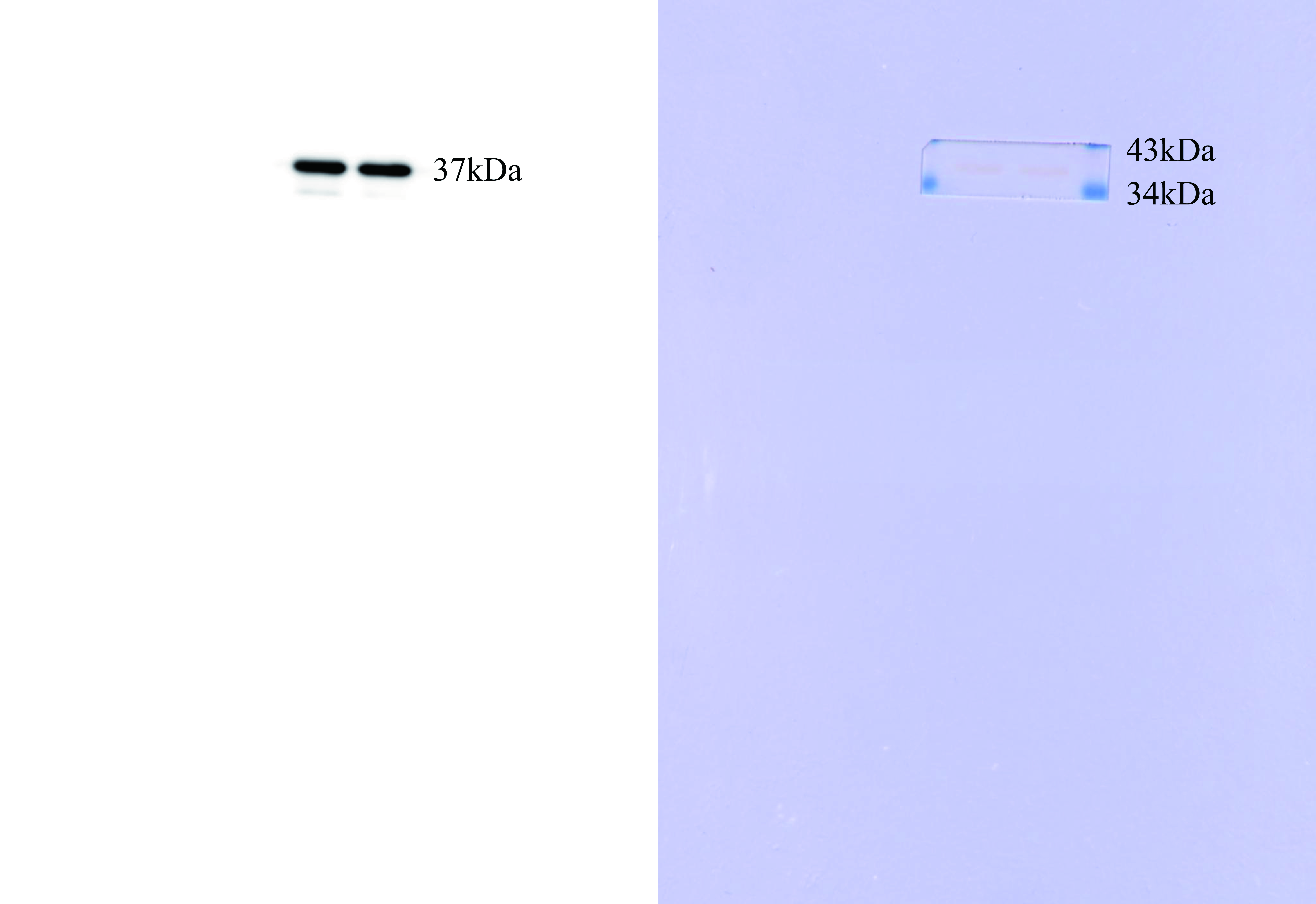

Supplement: Supplementary file 1 [file cancers-14-05434-s001.zip › FigS2-WB-PANC-1-GAPDH-3.jpg]

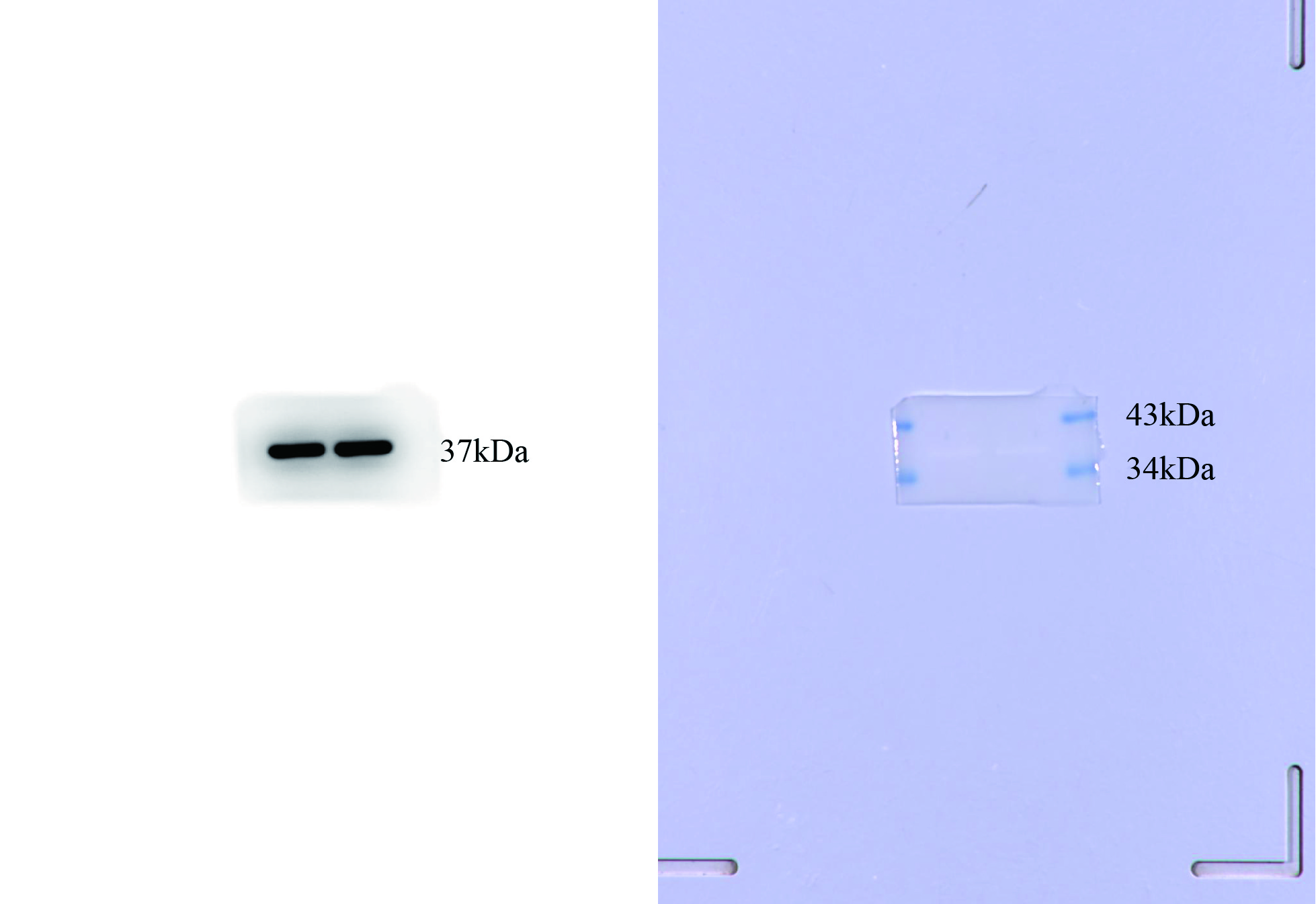

Supplement: Supplementary file 1 [file cancers-14-05434-s001.zip › FigS3-WB-PANC-1-LV-CDCA8 GAPDH-1.jpg]

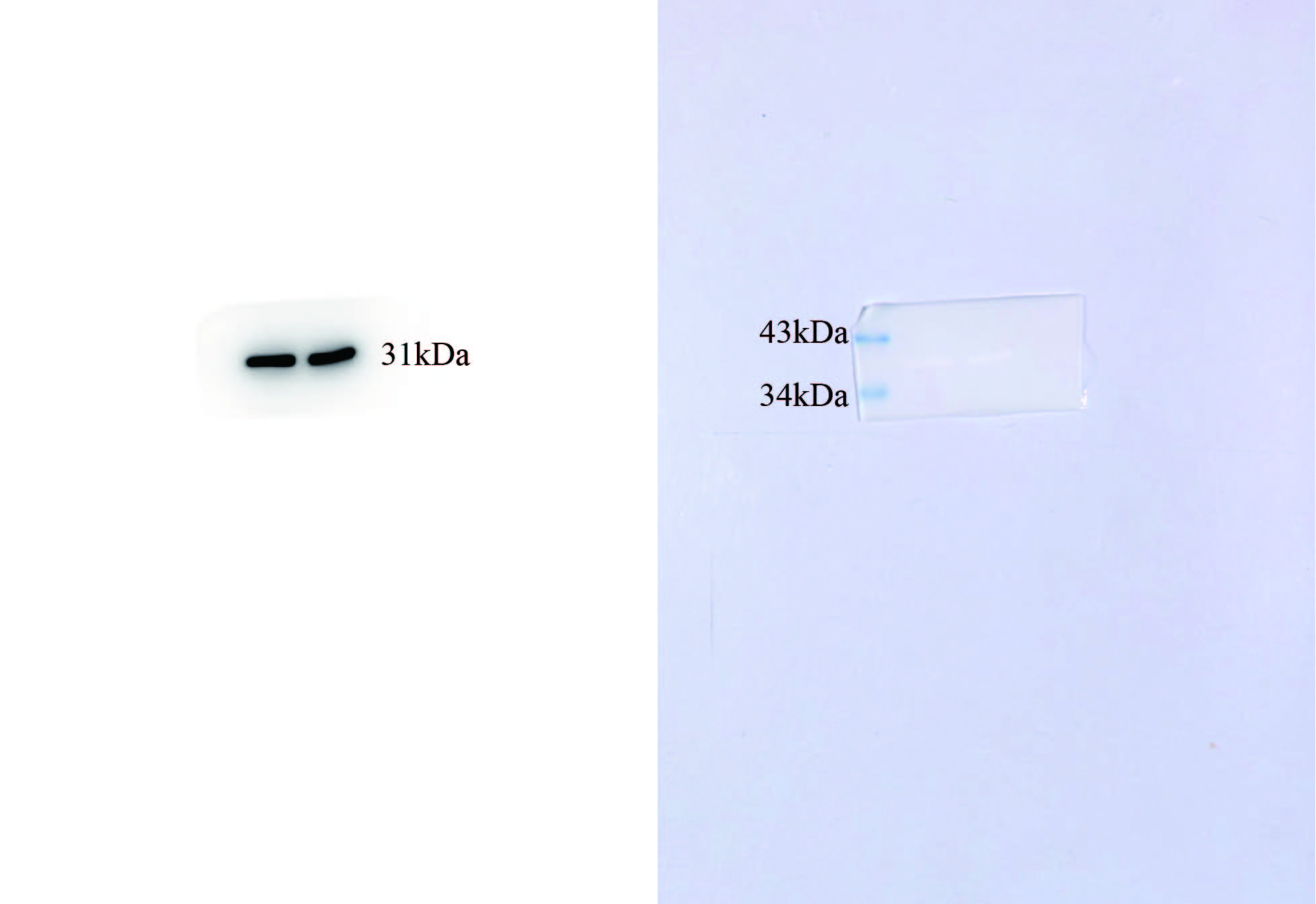

Supplement: Supplementary file 1 [file cancers-14-05434-s001.zip › FigS3-WB-PANC-1-LV-CDCA8 GAPDH-2.jpg]

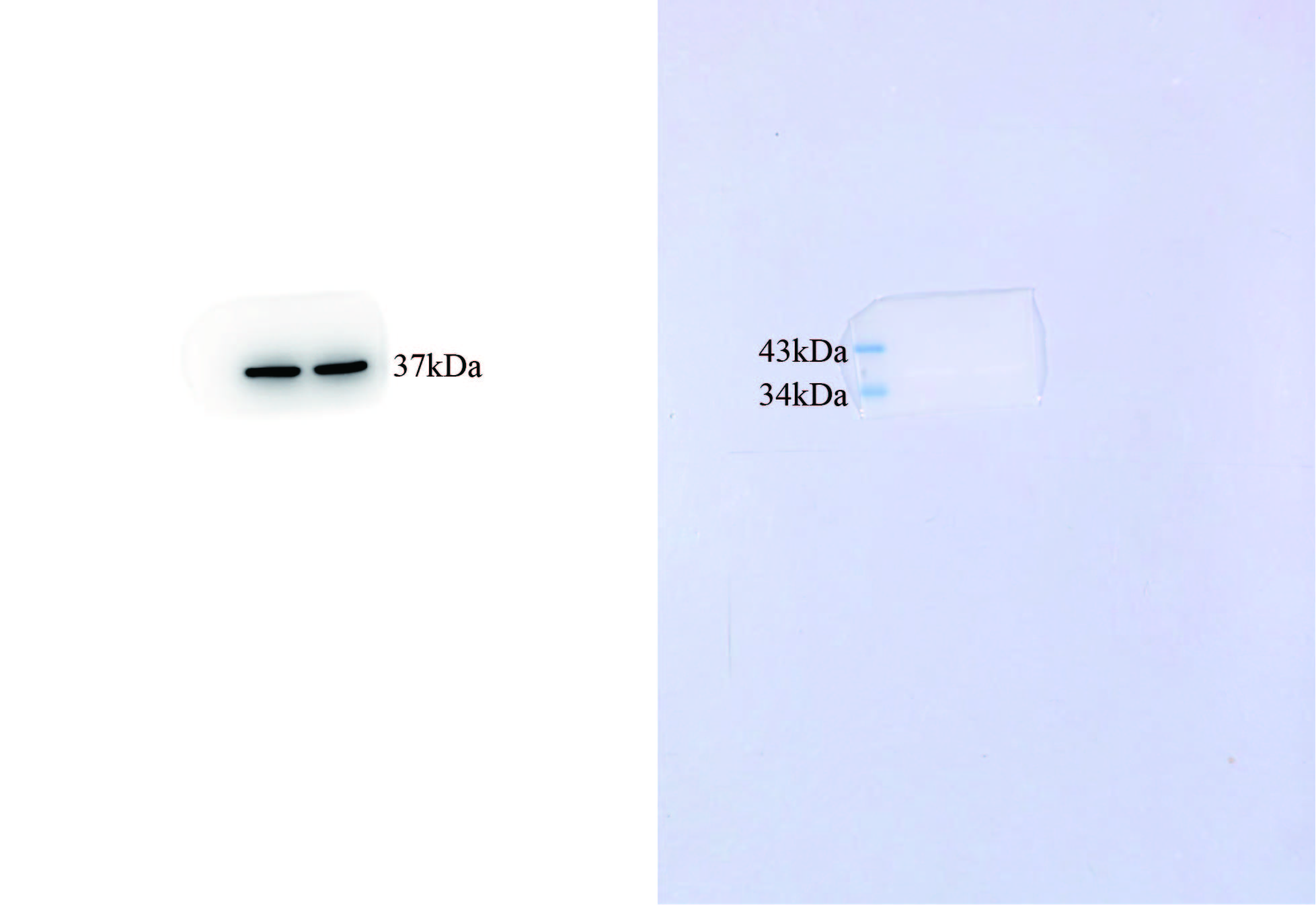

Supplement: Supplementary file 1 [file cancers-14-05434-s001.zip › FigS3-WB-PANC-1-LV-CDCA8 GAPDH-3.jpg]

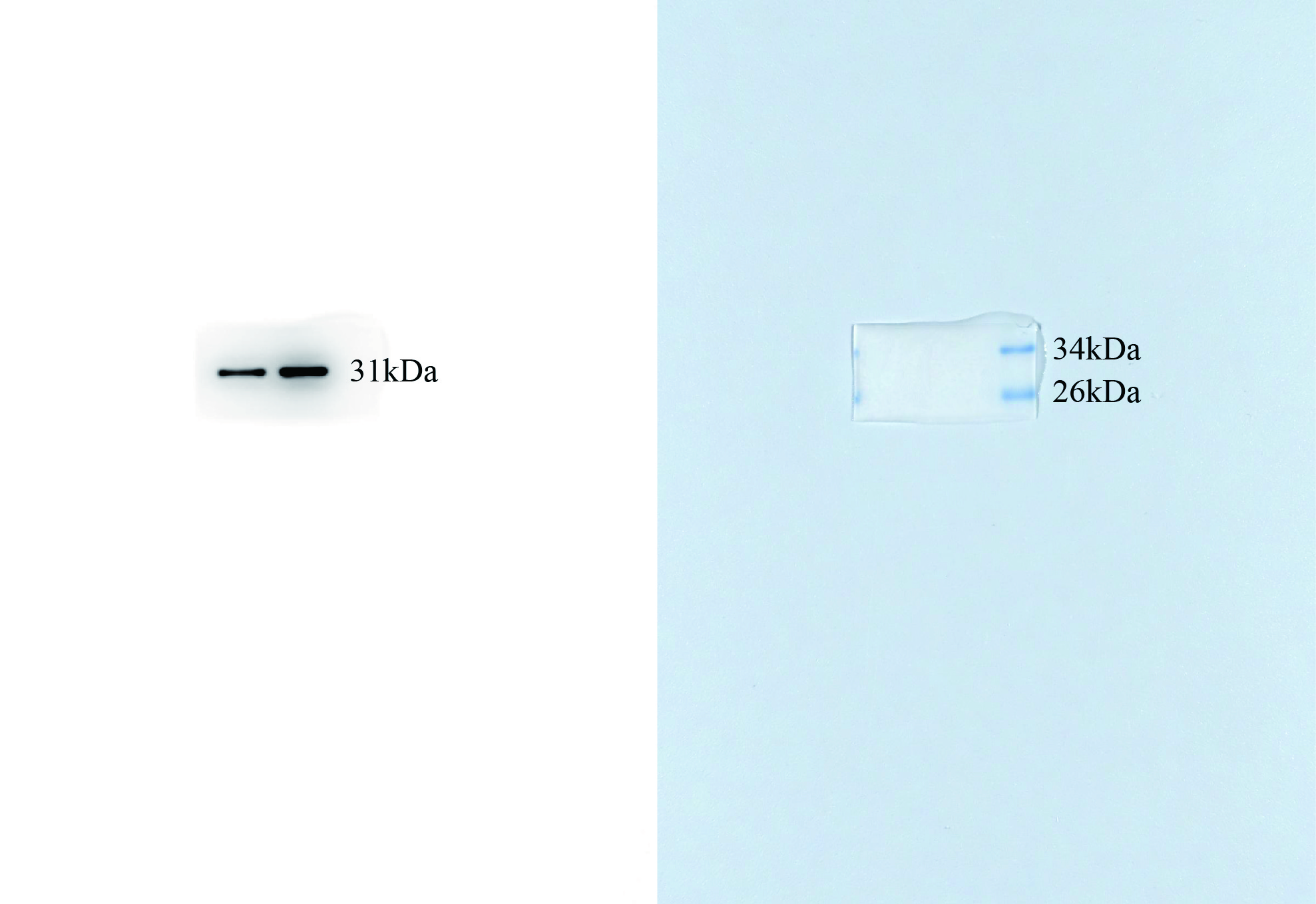

Supplement: Supplementary file 1 [file cancers-14-05434-s001.zip › FigS3-WB-PANC-1-LV-CDCA8-1.jpg]

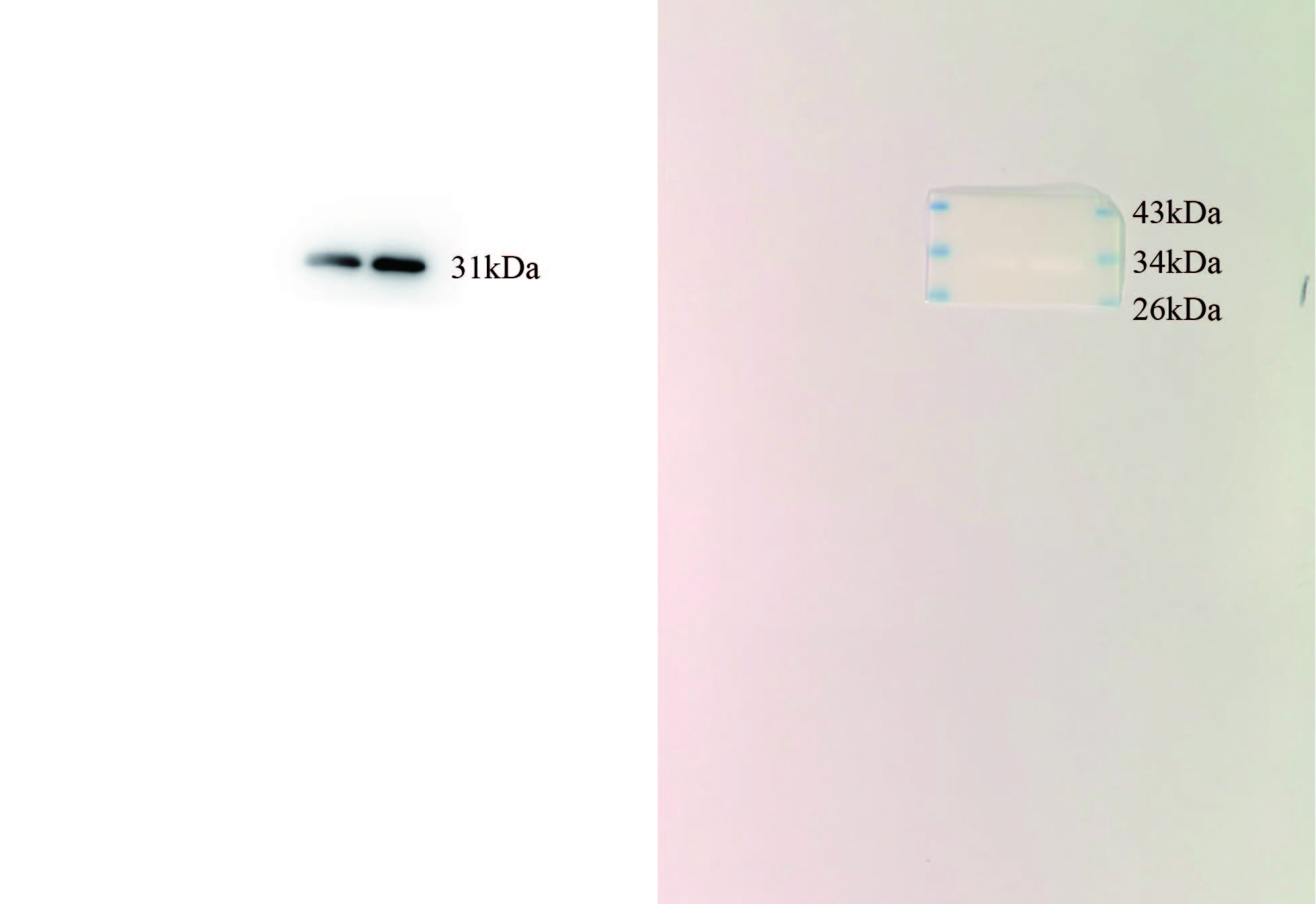

Supplement: Supplementary file 1 [file cancers-14-05434-s001.zip › FigS3-WB-PANC-1-LV-CDCA8-2.jpg]

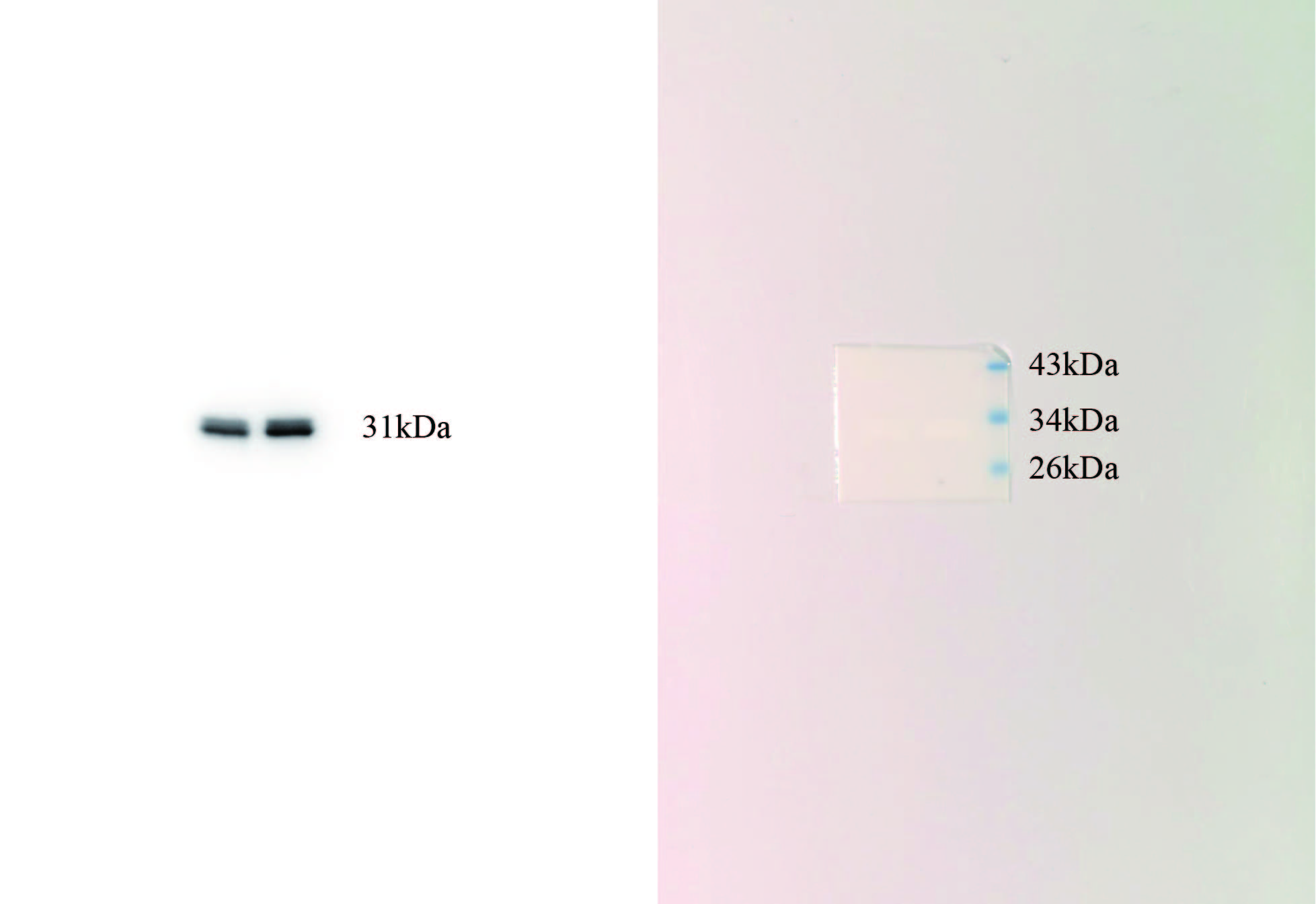

Supplement: Supplementary file 1 [file cancers-14-05434-s001.zip › FigS3-WB-PANC-1-LV-CDCA8-3.jpg]

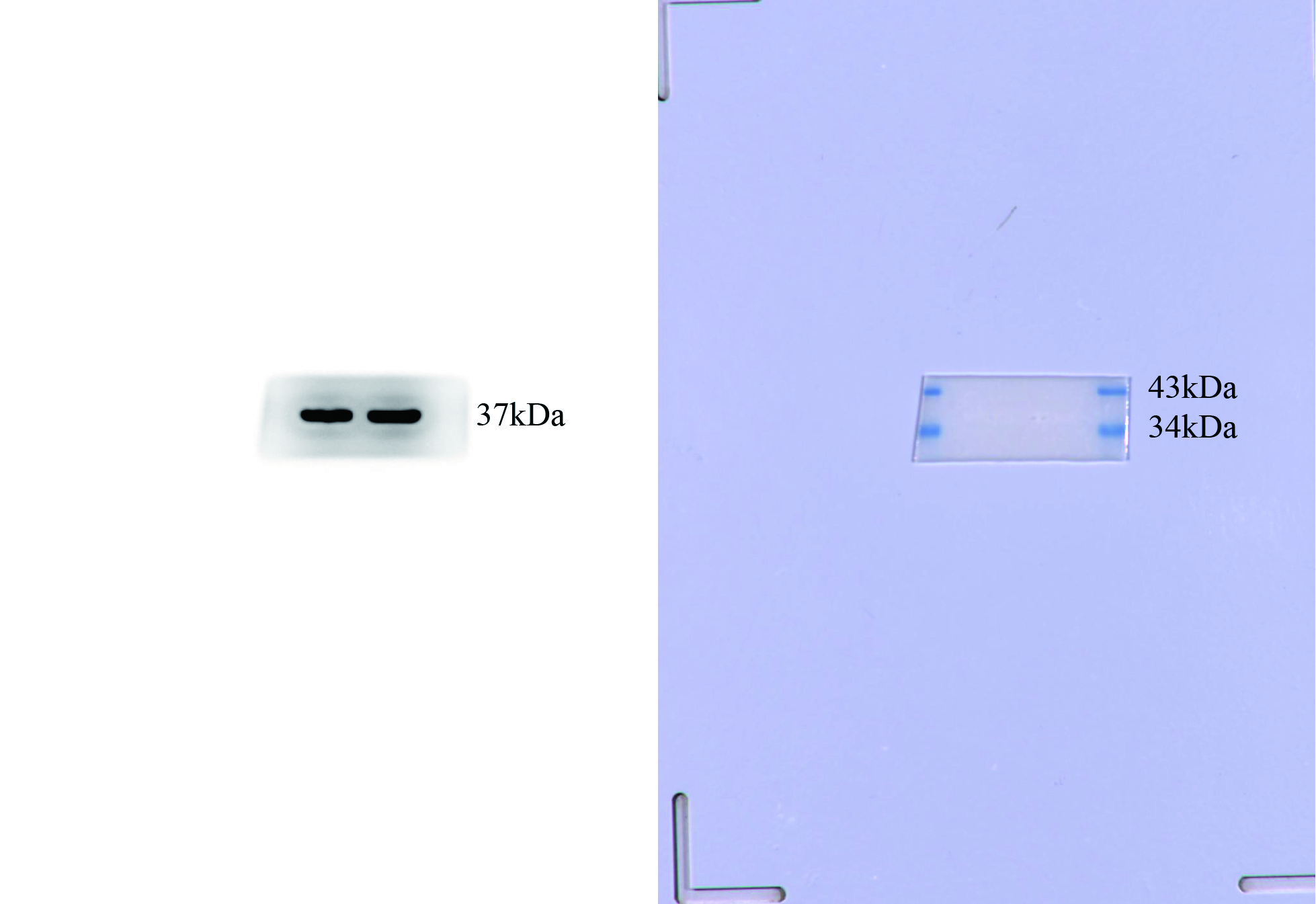

Supplement: Supplementary file 1 [file cancers-14-05434-s001.zip › FigS3-WB-PANC-1-shCD44 GAPDH-1.jpg]

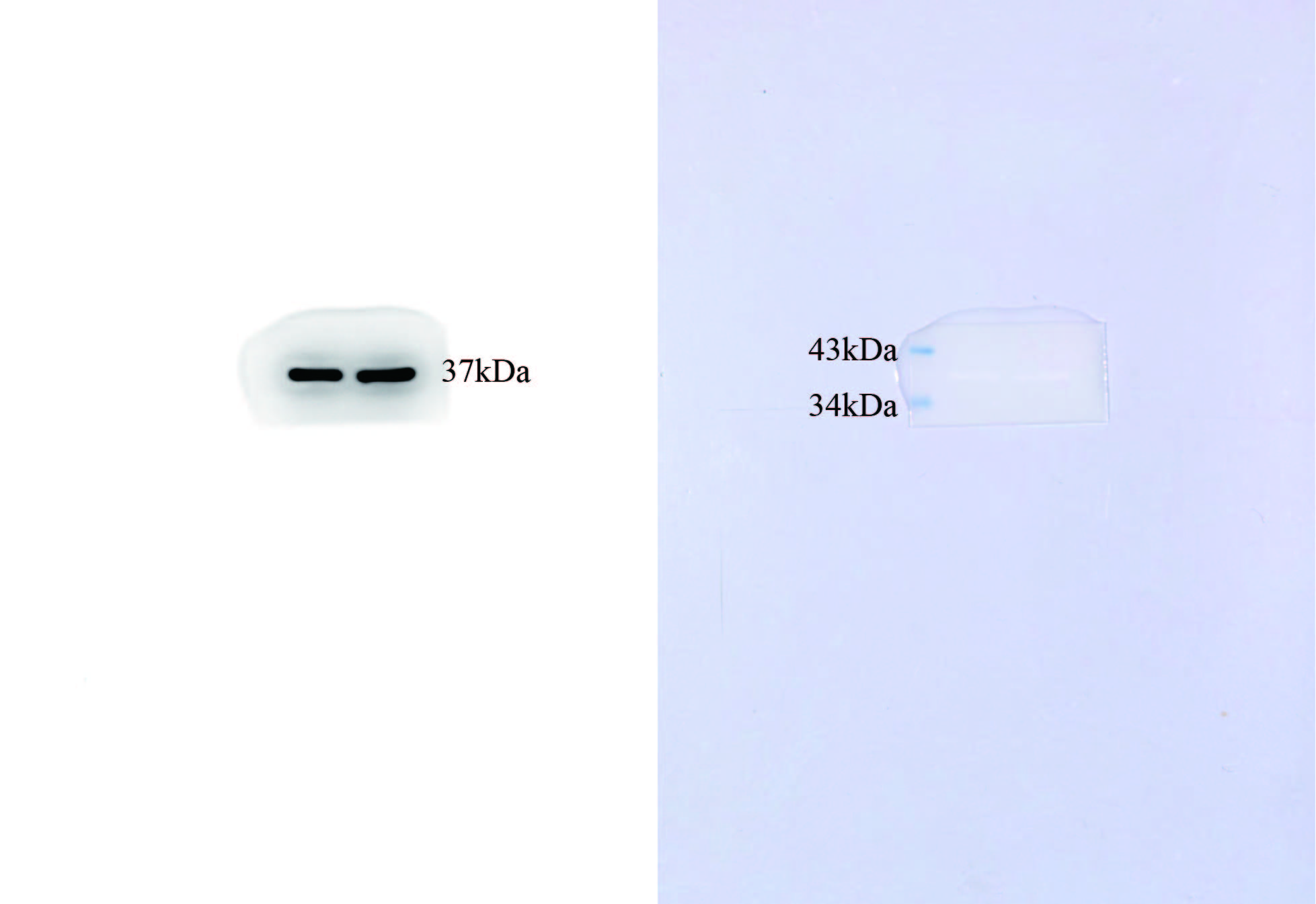

Supplement: Supplementary file 1 [file cancers-14-05434-s001.zip › FigS3-WB-PANC-1-shCD44 GAPDH-2.jpg]

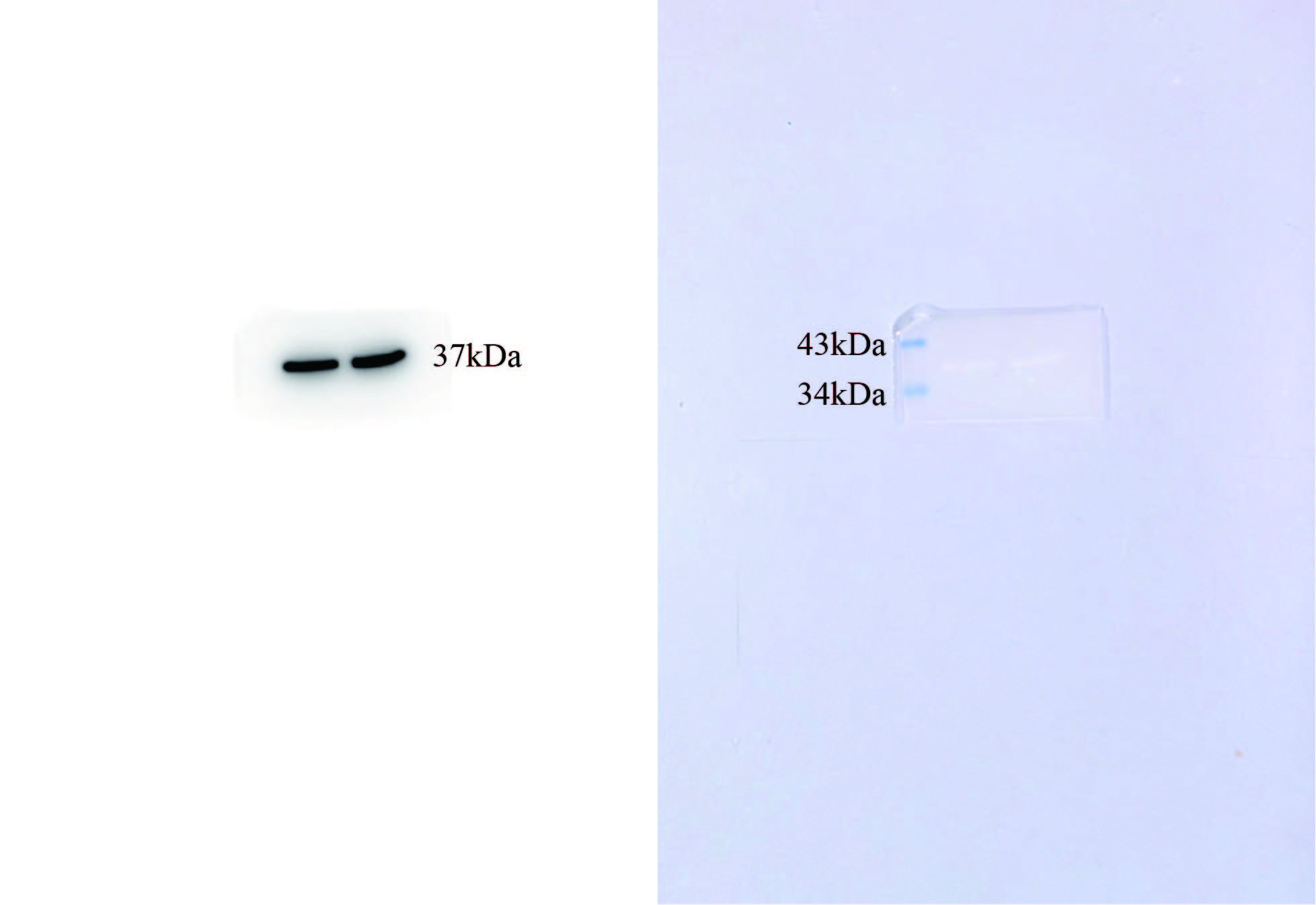

Supplement: Supplementary file 1 [file cancers-14-05434-s001.zip › FigS3-WB-PANC-1-shCD44 GAPDH-3.jpg]

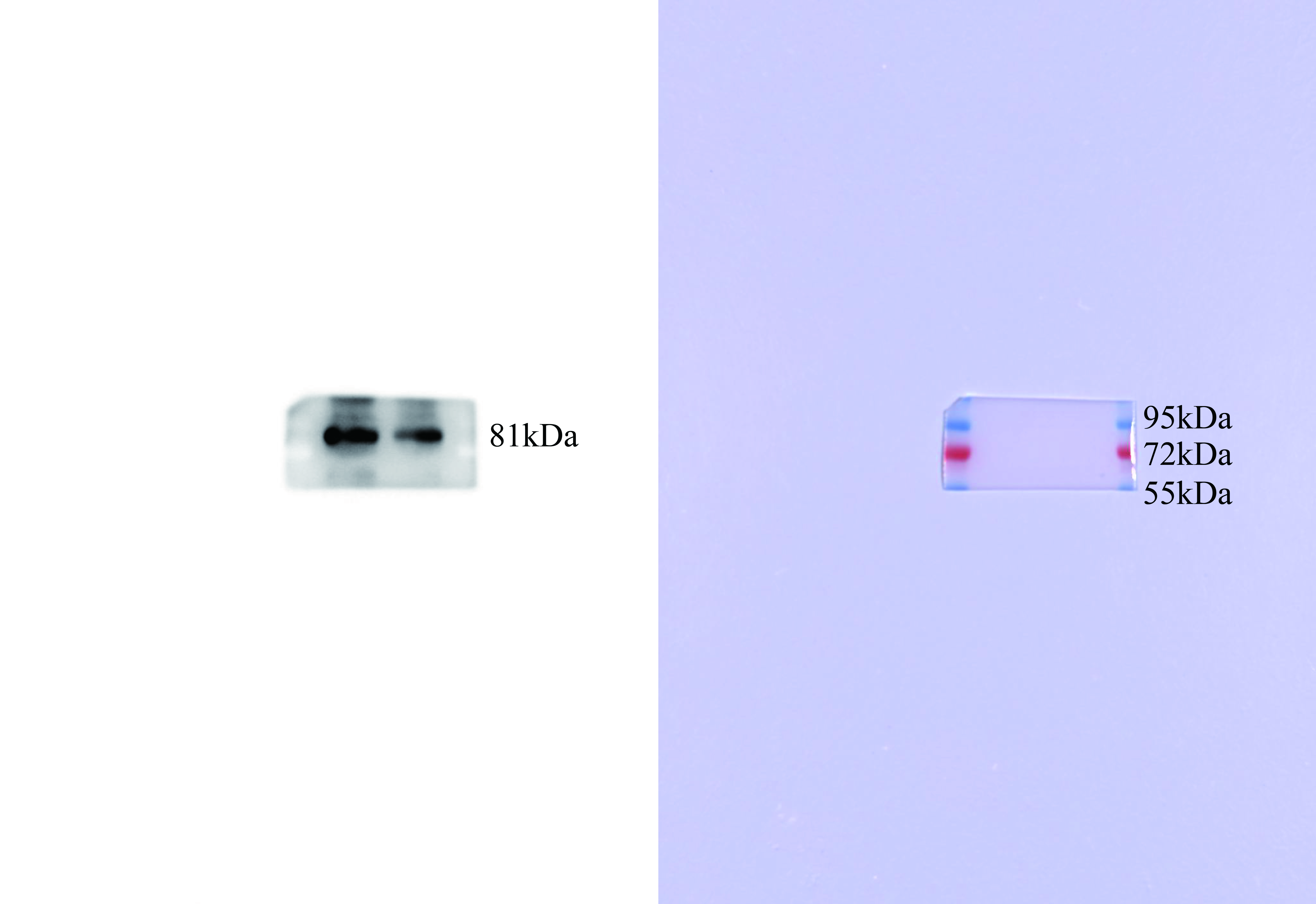

Supplement: Supplementary file 1 [file cancers-14-05434-s001.zip › FigS3-WB-PANC-1-shCD44-1.jpg]

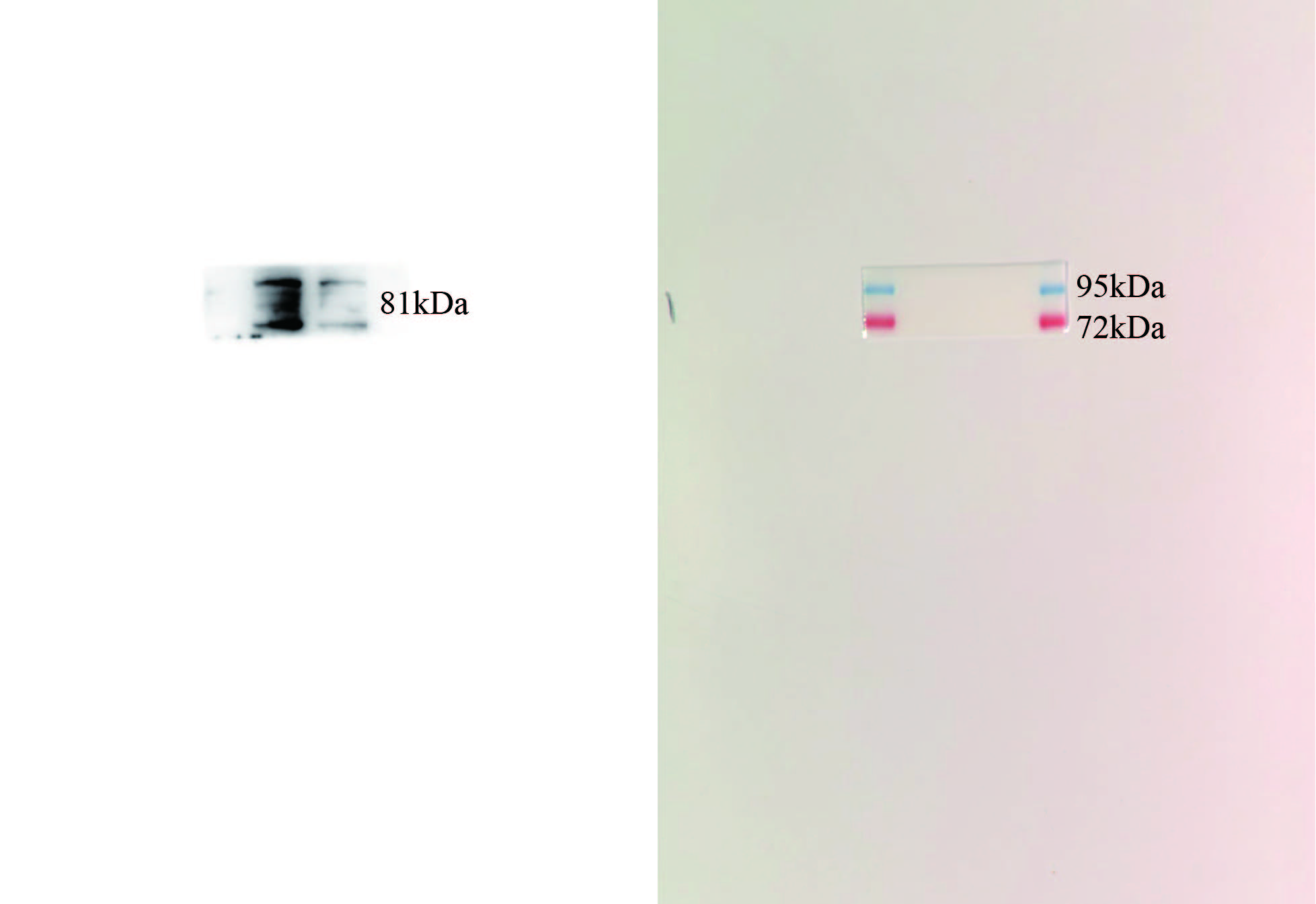

Supplement: Supplementary file 1 [file cancers-14-05434-s001.zip › FigS3-WB-PANC-1-shCD44-2.jpg]

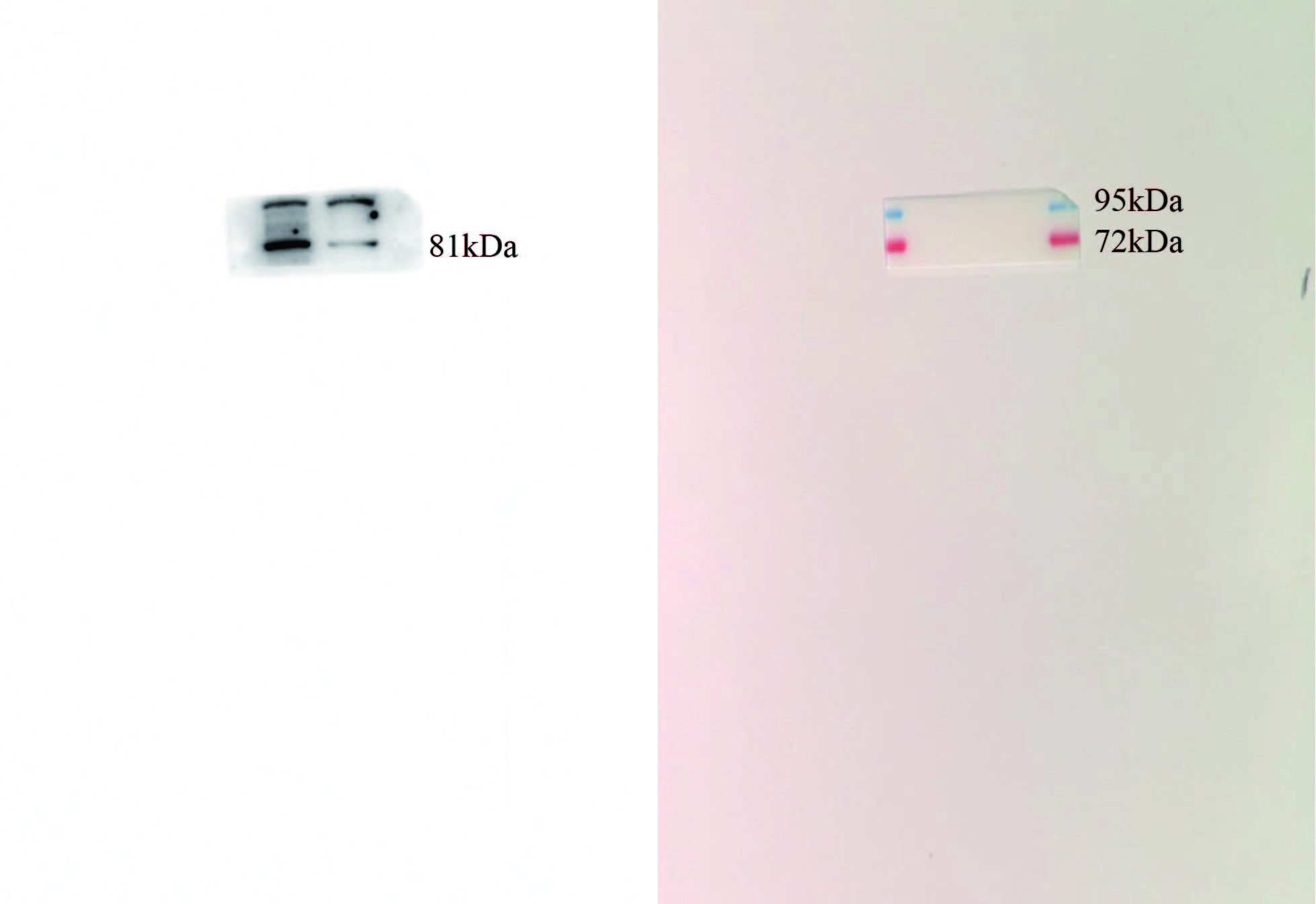

Supplement: Supplementary file 1 [file cancers-14-05434-s001.zip › FigS3-WB-PANC-1-shCD44-3.jpg]

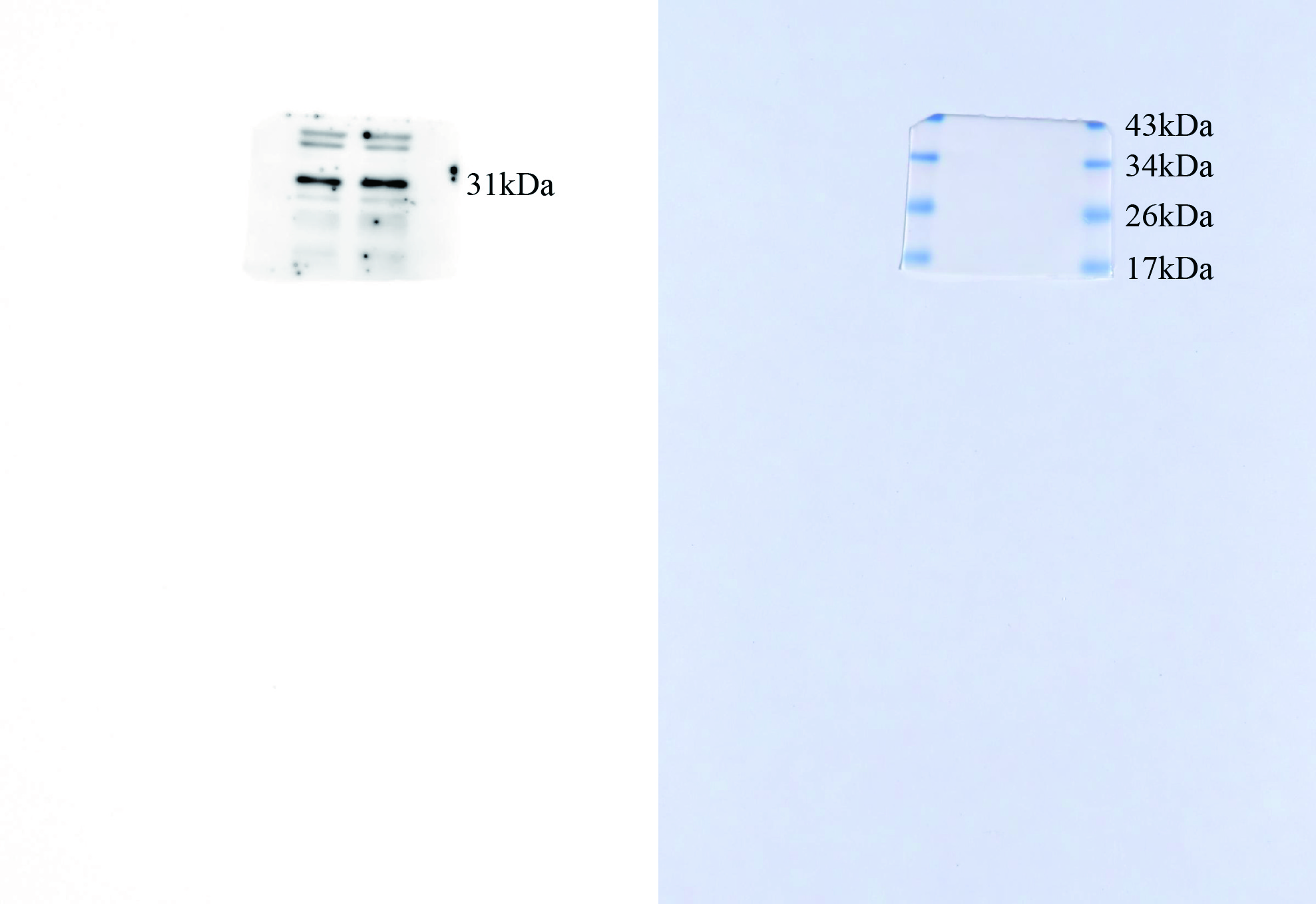

Supplement: Supplementary file 1 [file cancers-14-05434-s001.zip › FigS3-WB-SW-1990-LV-CDCA8-1.jpg]

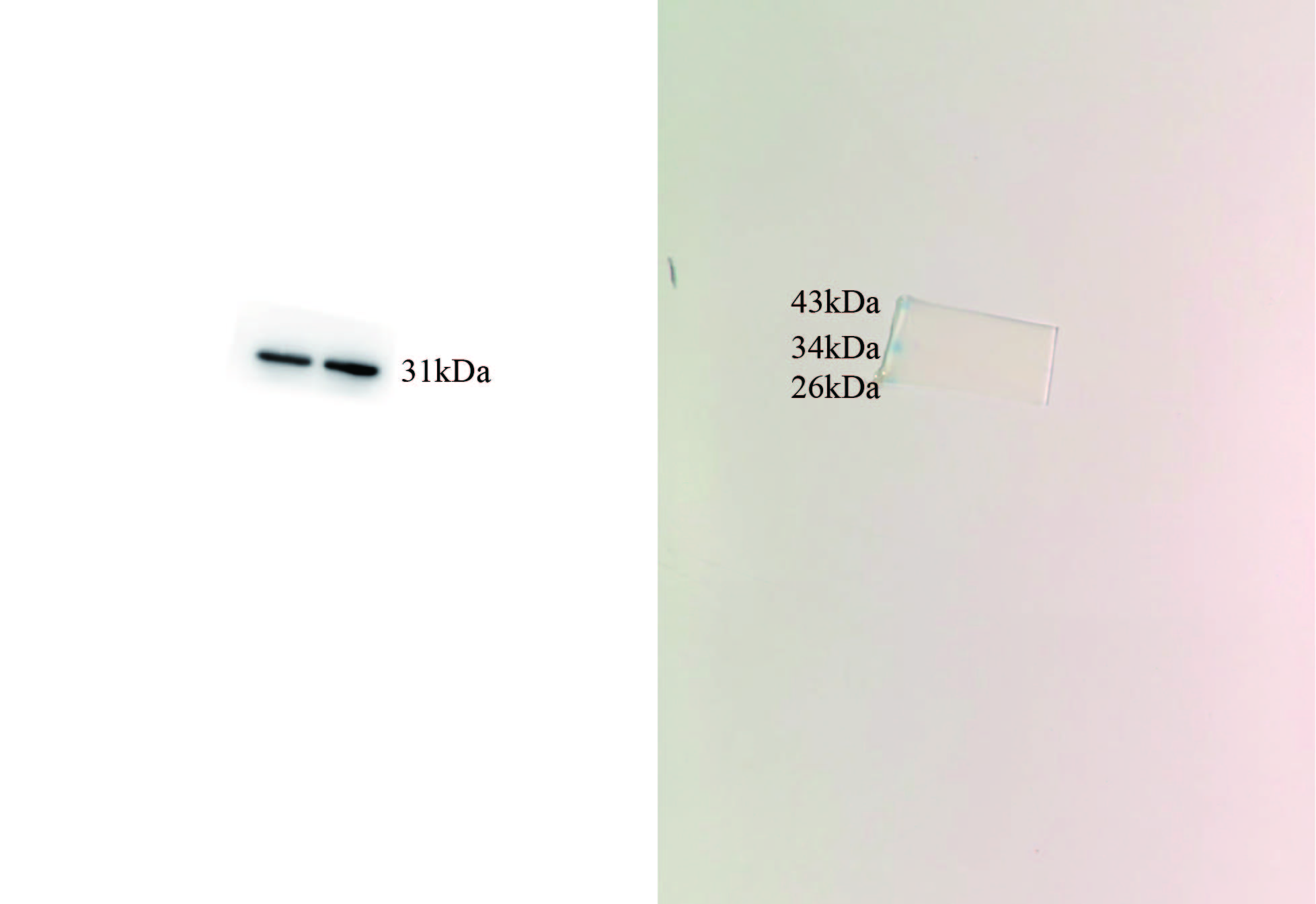

Supplement: Supplementary file 1 [file cancers-14-05434-s001.zip › FigS3-WB-SW-1990-LV-CDCA8-2.jpg]

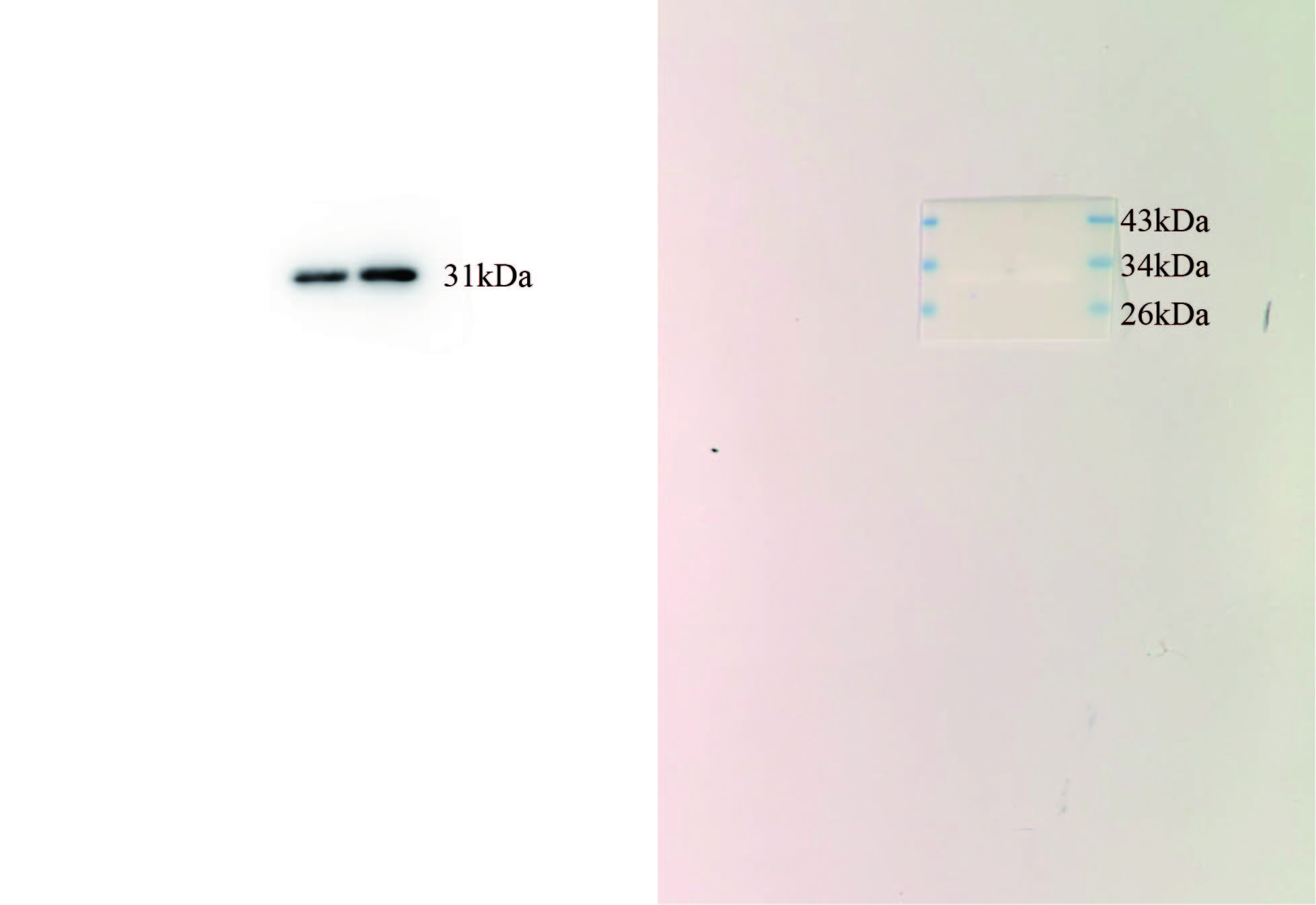

Supplement: Supplementary file 1 [file cancers-14-05434-s001.zip › FigS3-WB-SW-1990-LV-CDCA8-3.jpg]

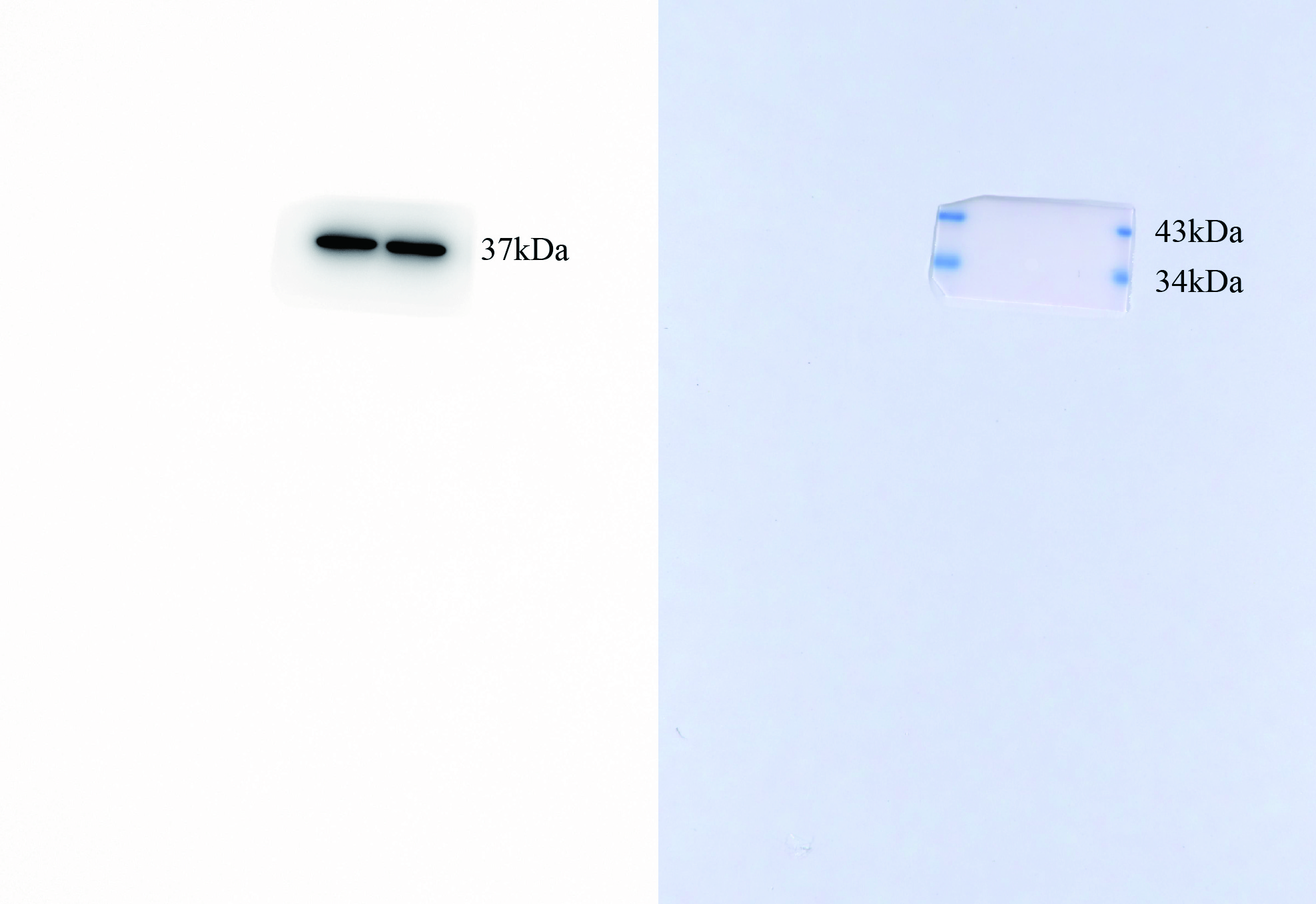

Supplement: Supplementary file 1 [file cancers-14-05434-s001.zip › FigS3-WB-SW-1990-LV-CDCA8-GAPDH-1.jpg]

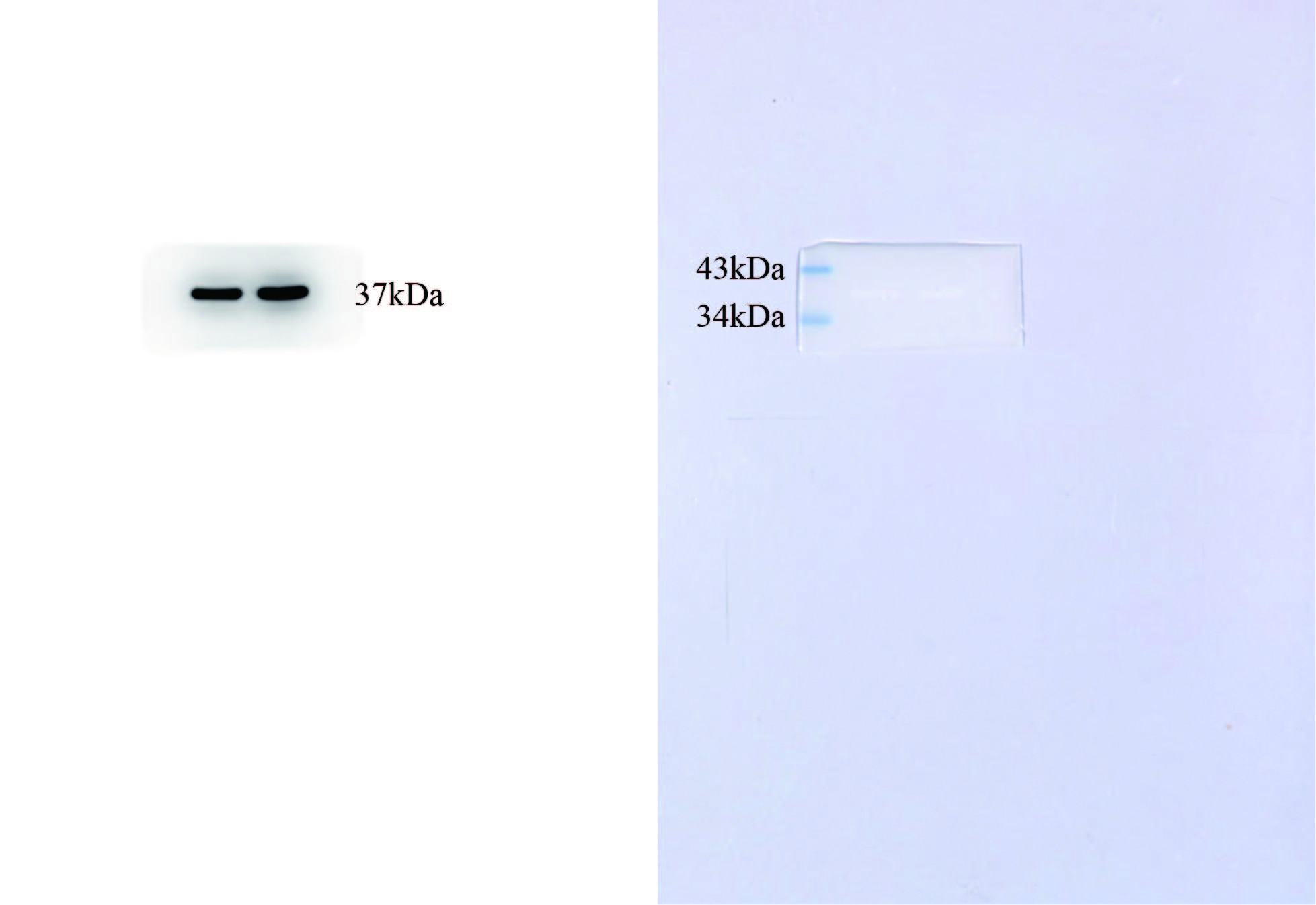

Supplement: Supplementary file 1 [file cancers-14-05434-s001.zip › FigS3-WB-SW-1990-LV-CDCA8-GAPDH-2.jpg]

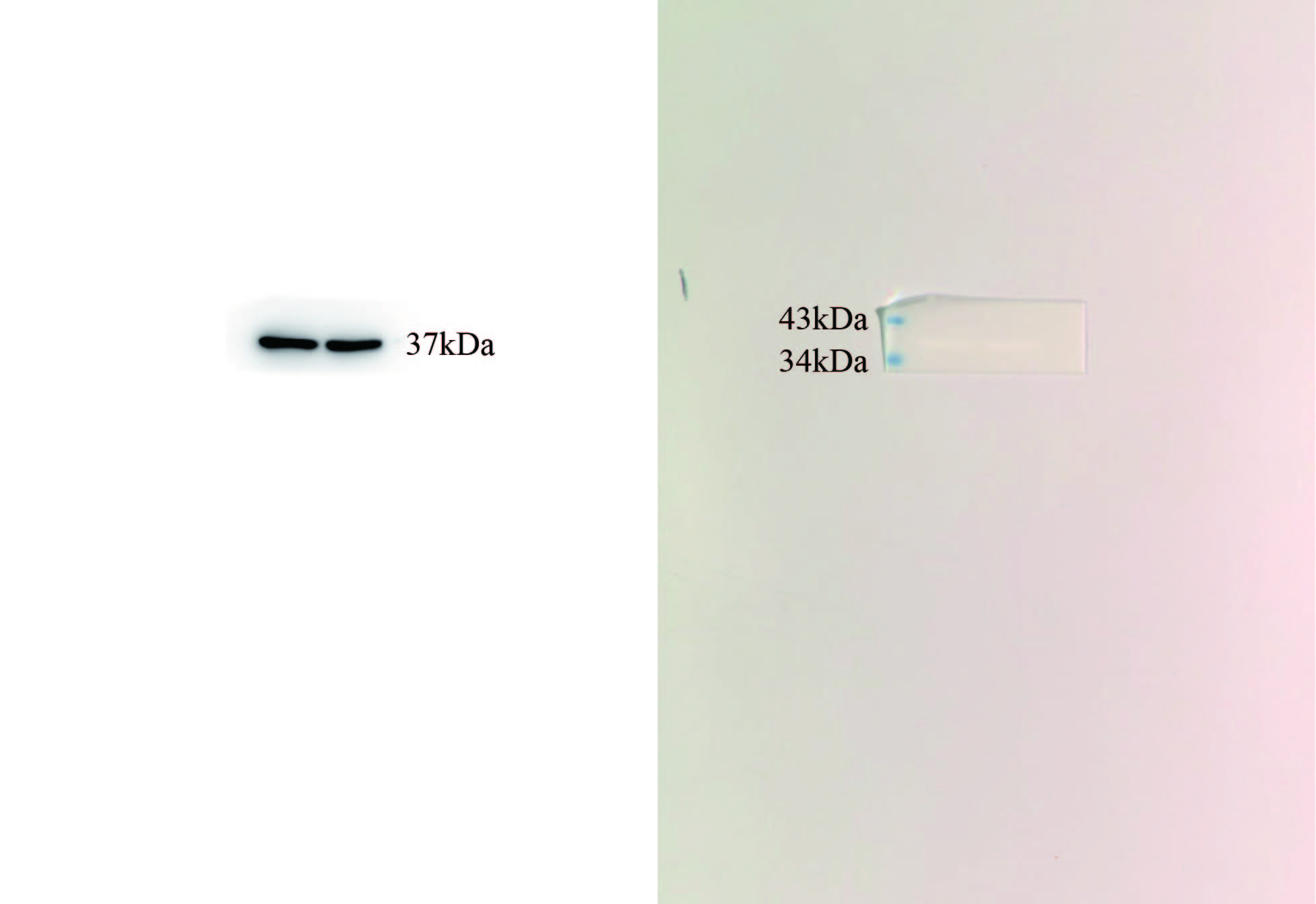

Supplement: Supplementary file 1 [file cancers-14-05434-s001.zip › FigS3-WB-SW-1990-LV-CDCA8-GAPDH-3.jpg]

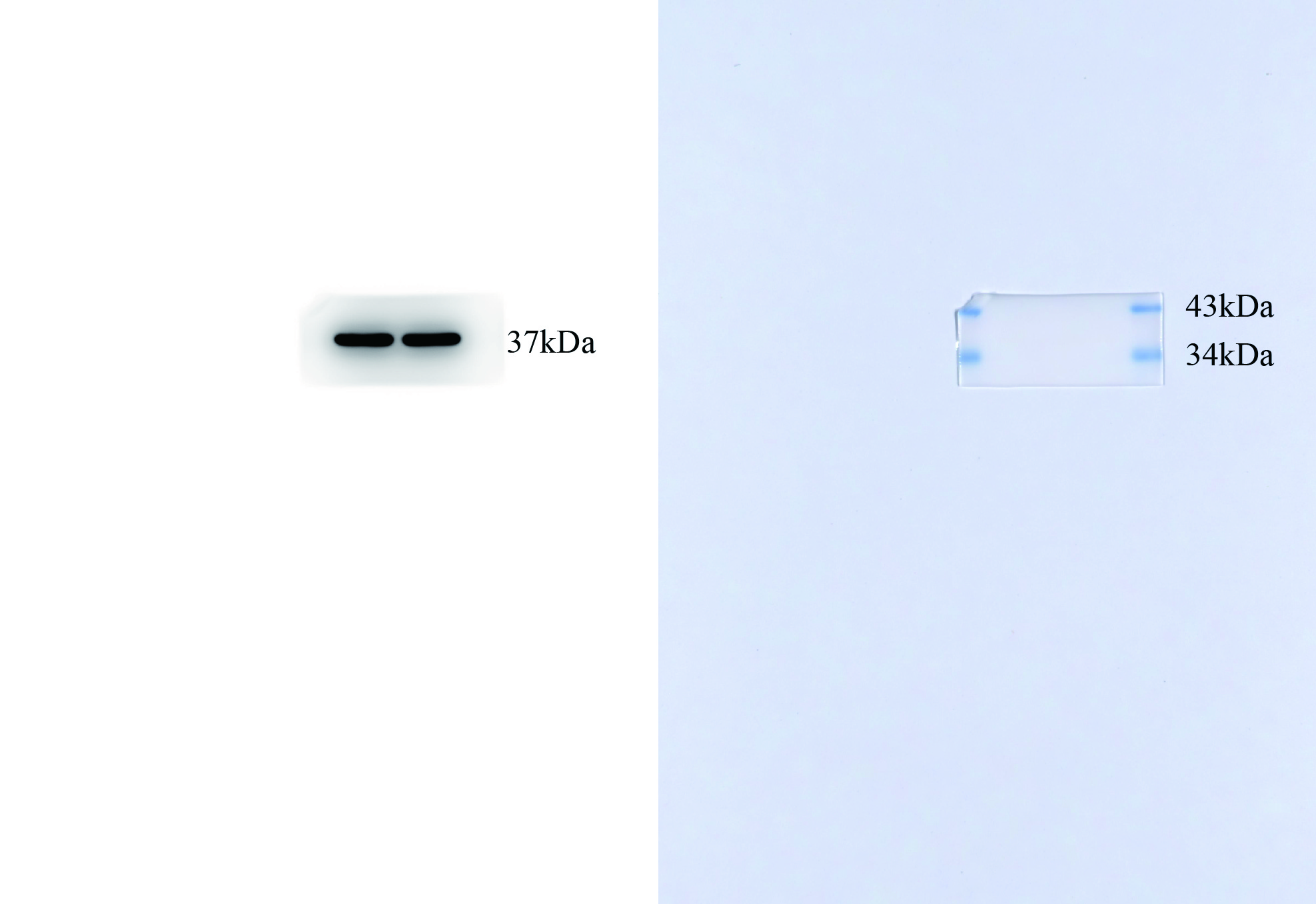

Supplement: Supplementary file 1 [file cancers-14-05434-s001.zip › FigS3-WB-SW-1990-shCD44 GAPDH-1.jpg]

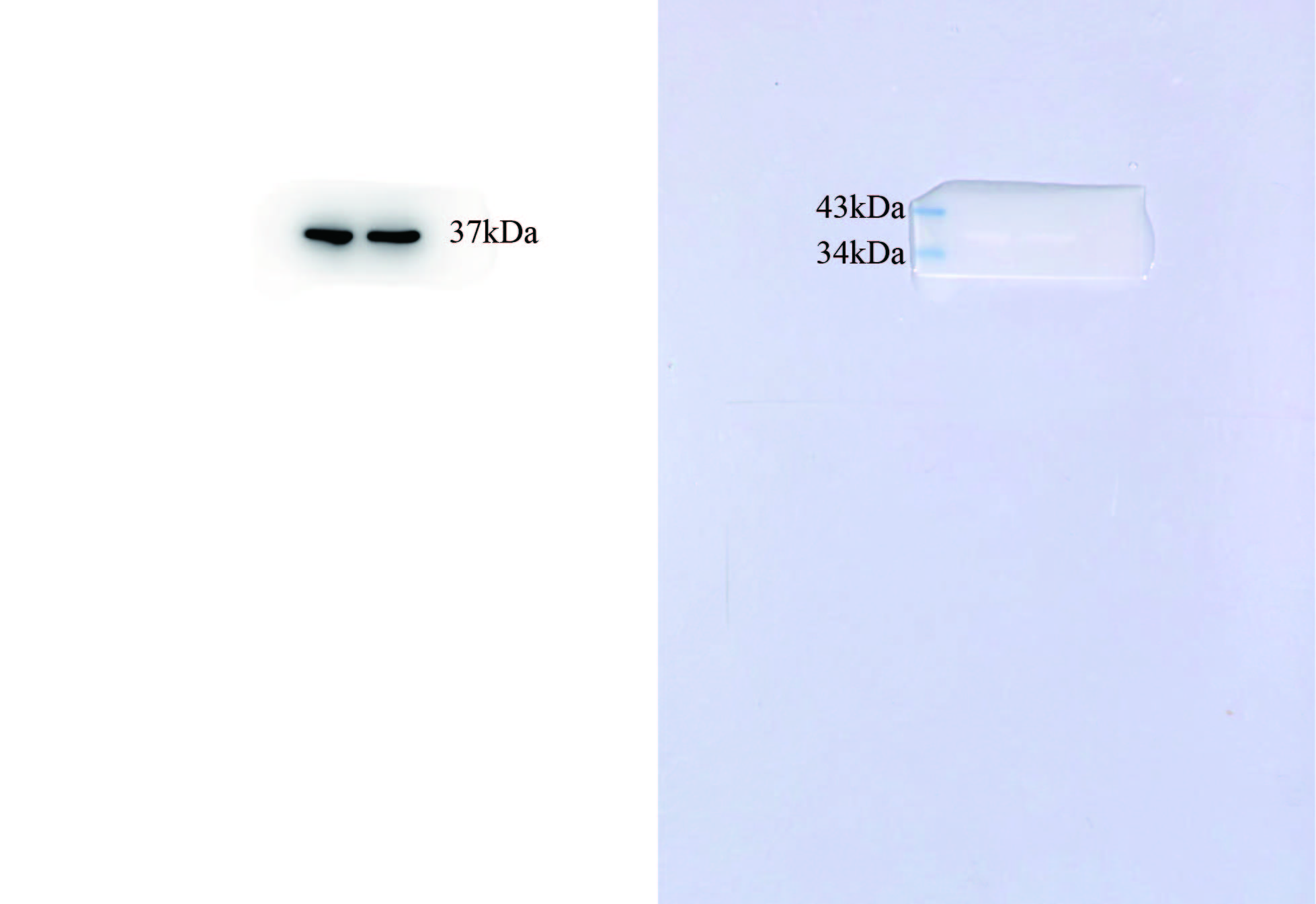

Supplement: Supplementary file 1 [file cancers-14-05434-s001.zip › FigS3-WB-SW-1990-shCD44 GAPDH-2.jpg]

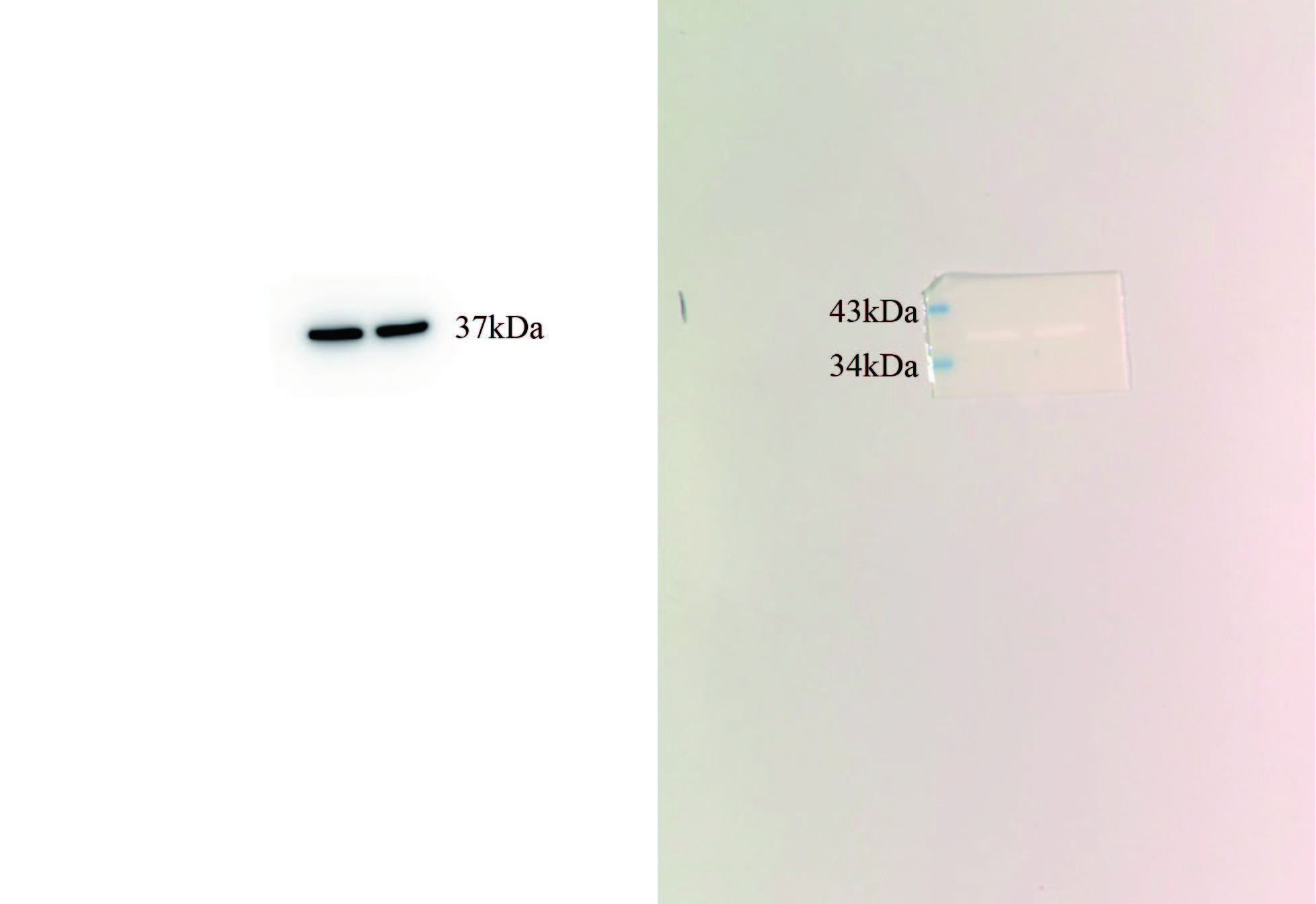

Supplement: Supplementary file 1 [file cancers-14-05434-s001.zip › FigS3-WB-SW-1990-shCD44 GAPDH-3.jpg]

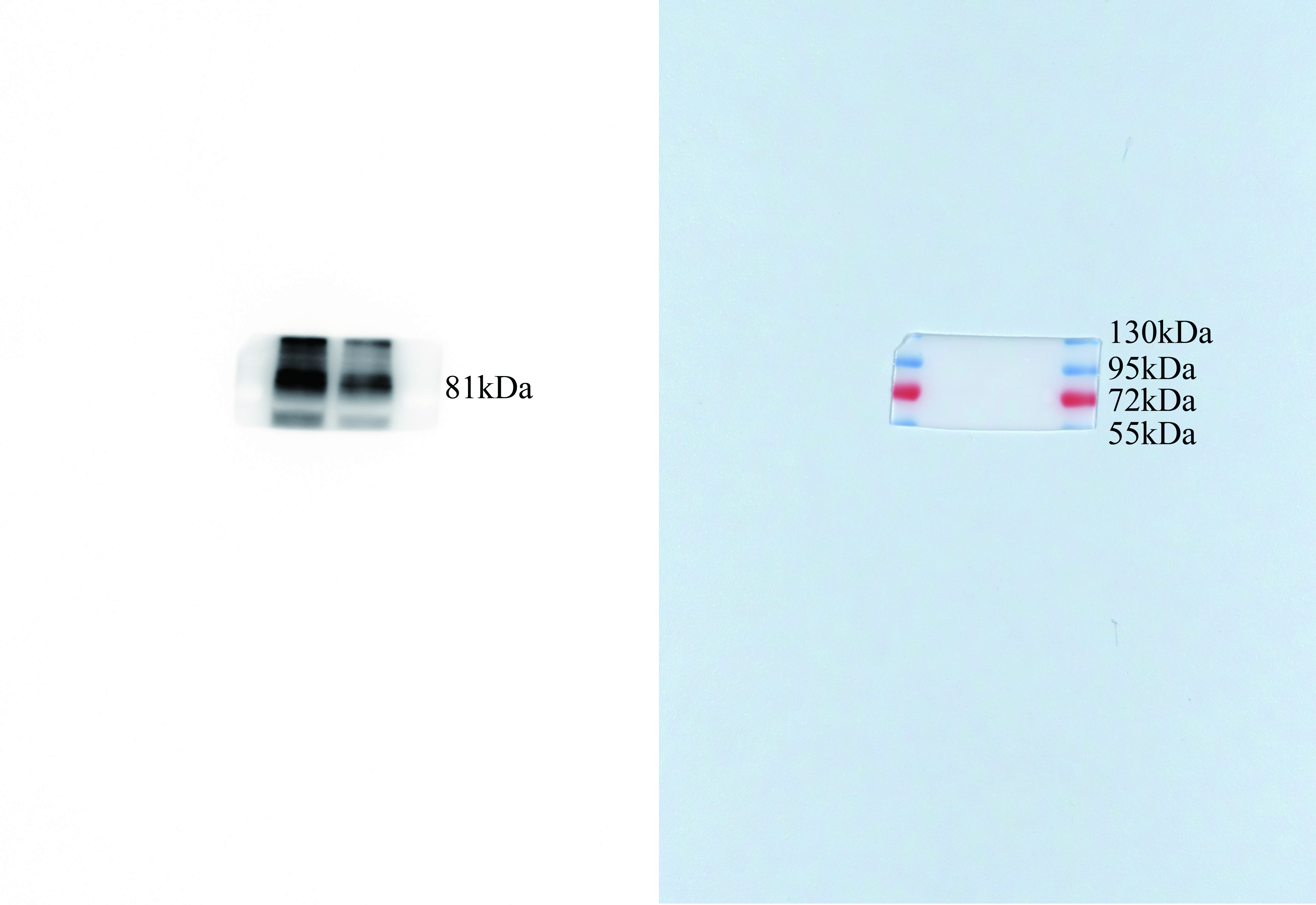

Supplement: Supplementary file 1 [file cancers-14-05434-s001.zip › FigS3-WB-SW-1990-shCD44-1.jpg]

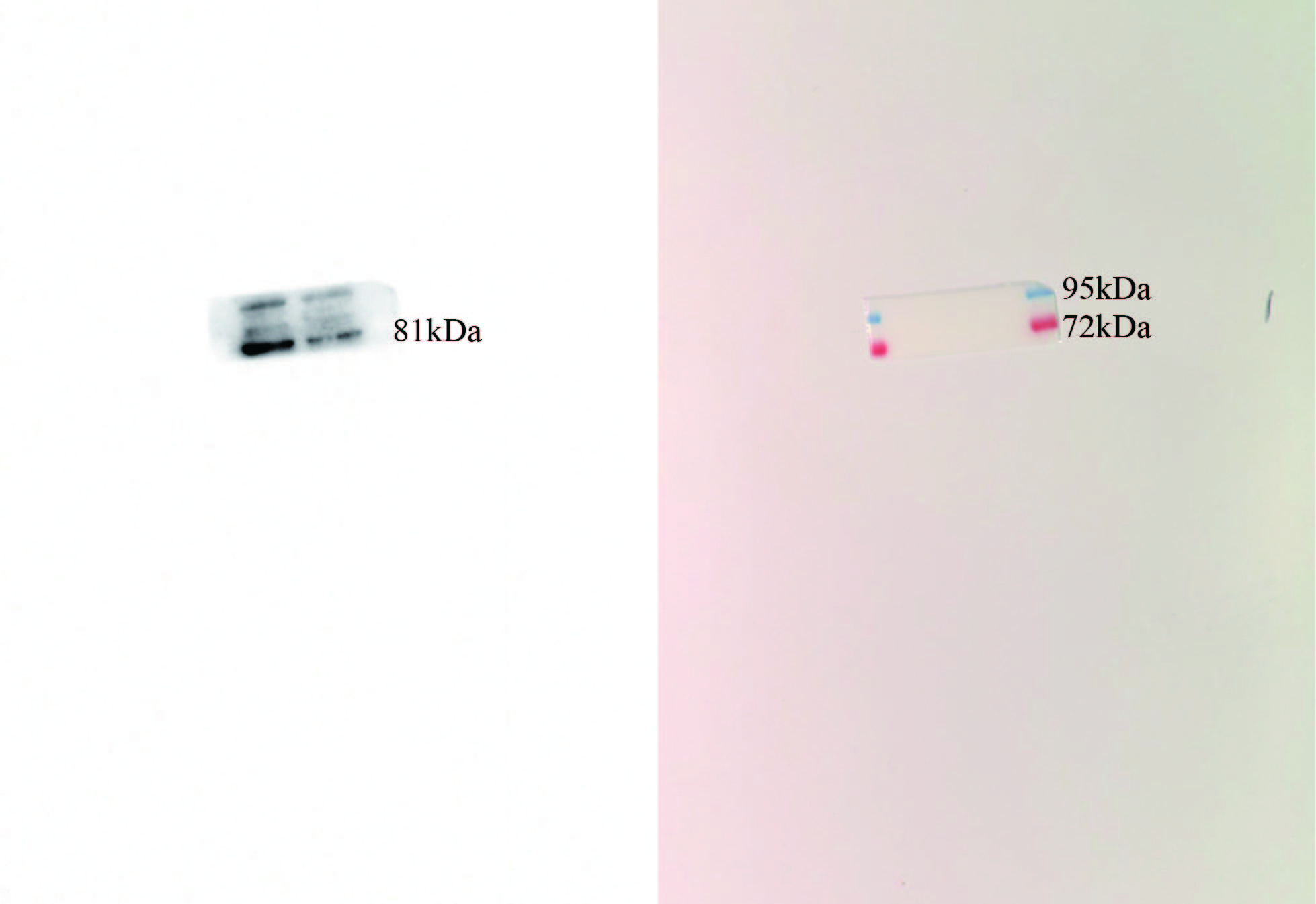

Supplement: Supplementary file 1 [file cancers-14-05434-s001.zip › FigS3-WB-SW-1990-shCD44-2.jpg]

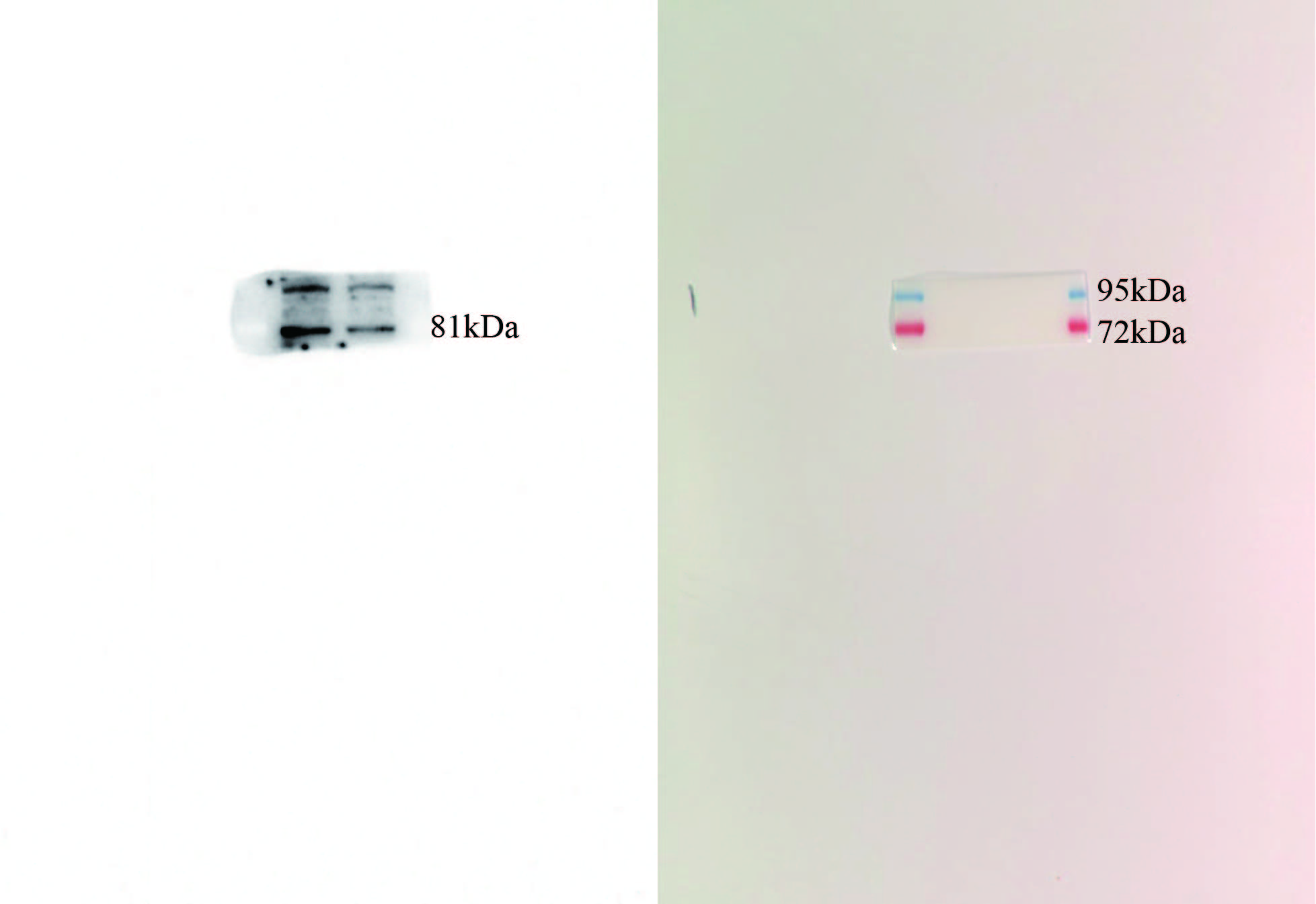

Supplement: Supplementary file 1 [file cancers-14-05434-s001.zip › FigS3-WB-SW-1990-shCD44-3.jpg]
